# A photoperiod-responsive protein compendium and conceptual proteome roadmap outline in maize grown in growth chambers with controlled conditions

**DOI:** 10.1371/journal.pone.0174003

**Published:** 2017-04-11

**Authors:** You-Zhi Li, Xian-Wei Fan, Qiang Chen, Hao Zhong

**Affiliations:** State Key Laboratory for Conservation and Utilization of Subtropical Agro-bioresources, College of Life Science and Technology, Guangxi University, Nanning, P. R. China; Henan Agricultural University, CHINA

## Abstract

Maize (*Zea mays* L.) is one of the major staple food crops of the world. However, high photoperiod sensitivity, especially for tropical germplasms, impedes attempts to improve maize agronomical traits by integration of tropical and temperate maize germplasms. Physiological and phenotypic responses of maize to photoperiod have widely been investigated based on multi-site field observations; however, proteome-based responsive mechanisms under controlled photoperiod regimes, nutrient and moisture soils are not yet well understood. In the present study, we sequenced and analyzed six proteomes of tropically-adapted and photoperiod-sensitive M9 inbred line at the vegetative 3 stage and proteomes from tropically-adapted and photoperiod-sensitive Shuang M9 (SM9) inbred line at the vegetative-tasseling stage. All plants were grown in growth chambers with controlled soil and temperature and three photoperiod regimes, a short photoperiod (SP) of 10 h light/14 h dark, a control neutral photoperiod (NP) of 12 h light/12 h dark, and a long photoperiod (LP) of 16 h light/8 h dark for a daily cycle. We identified 4,395 proteins of which 401 and 425 differentially-expressed proteins (DPs) were found in abundance in M9 leaves and in SM9 leaves as per SP/LP vs. NP, respectively. Some DPs showed responses to both SP and LP while some only responded to either SP or LP, depending on M9 or SM9. Our study showed that the photoperiodic response pathway, circadian clock rhythm, and high light density/intensity crosstalk with each other, but apparently differ from dark signaling routes. Photoperiod response involves light-responsive or dark-responsive proteins or both. The DPs positioned on the signaling routes from photoperiod changes to RNA/DNA responses involve the mago nashi homolog and glycine-rich RNA-binding proteins. Moreover, the cell-to-cell movement of ZCN14 through plasmodesmata is likely blocked under a 16-h-light LP. Here, we propose a photoperiodic model based on our findings and those from previous studies.

## Introduction

Photoperiod is a daily recurring pattern of light and dark periods [1]. The response or capacity to respond to photoperiod is termed as photoperiodism [1]. However, photoperiod sensing is also partially associated with mechanisms regulating the circadian rhythms [2–4].

Maize (*Zea mays* L.), a major food crops of the world, originated in tropics [5] but evolved into tropically-adapted photoperiod-sensitive and temperate-adapted photoperiod insensitive germplasms due to post-domestication and breeding selection [6]. However, the high sensitivity of tropical maize germplasms to photoperiods limits its planting distribution and production [5, 7]. Photoperiod-sensitive maize lines/hybrids with tropical germplasm are characterized in part by delayed flowering and/or failure of seed setting under long photoperiods (LP) [8]. Three models for the effects of photoperiod on maize flowering have been proposed in maize [6, 9, 10], which are framed by several genes-encoded proteins.

Soil conditions such as soil moisture [11] and **flooding** [12] may affect plant responses to photoperiod changes. Increasing attempts to improve maize agronomical traits by integration of tropical and temperate maize germplasm [13, 14] is greatly impeded by the higher sensitivity of tropically-adapted maize to photoperiod changes [7, 15]. Therefore, it is important to unravel the molecular mechanisms governing photoperiod sensitivity of maize at different levels. The results based on multi-site field observations and/or on uncontrolled conditions can not accurately characterize the mechanisms of maize responses to changes in photoperiod factors. Our previous experiments conducted under controlled conditions in chambers showed that photoperiod changes can cause a wide variety of phenotypic changes in maize [8]. This indicates that changes in photoperiod may affect many signaling networks in maize cells, which should be addressed at the proteome level. For example, why LP conditions lead to a failure of photoperiod-sensitive maize lines in flowering is not clear. Moreover, the routes of the photoperiodic effects, light- and dark-responses, high light intensity, and circadian clock rhythms remain undeciphered.

Proteomics provides a complementary approach to genomics technologies by *en masse* interrogation of biological phenomena at the protein level [16]. Our previous study found that tropically-adapted M9 and Shuang M9 (SM9) inbred lines were photoperiod-sensitive and did not develop growth points at the stem apex, flowering and seed setting under the influence of an LP of 16 h day light under controlled air humidity and soil conditions at a constant temperature of 28°C [8]. Therefore, we conducted the present study on the proteome profiles of M9 and SM9 inbred lines by using the isobaric tag for relative and absolute quantitation (iTRAQ) to explore the endogenous protein networks governing the photoperiodic effects.

## Materials and methods

### Maize inbred lines and growth conditions

In this study, we used two photoperiod-sensitive inbred maize lines, M9 and SM9 [8]. The previous study under controlled conditions indicated that the photoperiodic effects on maize started early at the vegetative 3 (V3) [8].

The methods for the plantation and management of maize were as described in our previous study [8]. Briefly, the used soil was arable layer topsoil from the experimental field of Hainan Institute of Tropical Agricultural Resources, Sanya, Hainan, China. The arable layer topsoil was fully mixed together with the organic fertilizer (Haide Institute of Tropical Agricultural Resources Ltd. Hainan) in a ratio of 1:3, in which the final major effective nutrient content was 502.697 mg/kg for the available phosphorus, 2030 mg/kg for the available potassium, and 580 mg/kg for the alkaline-hydrolyzable nitrogen. The soil was then potted for planting maize, 14 kg per pot (which was of a 29 cm inner diameter at the top, 22.5 cm inner diameter at the bottom and 23.5 cm in height). The potted soil was irrigated by spraying local well water to obtain a moisture level of 95% before sowing maize seeds. The maize seed-planted pots were placed in the growth chamber with a constant room temperature of 28 ± 0.5°C. Photoperiods of the chambers were set up as a short photoperiod (SP) of 10 h light/14 h dark, a control neutral photoperiod (NP) of 12 h light/12 h dark, and LP of 16 h light/8 h dark for a daily cycle, respectively. NL-S600/230GN2A plant fill light ballasts (Newlight Electronics Co., Ltd.) were used as a light source, which provided light of intensity 13.71 cd/m^2^. The luminous intensity on the surface on the uppermost leaves was 13.71 cd/m^2^. The water content of the soil in the pots was controlled within the range of 65%–70% by spraying with water.

### Leaf tissue materials

Leaf tissues from maize lines at the V3 and vegetable-tasseling (VT) stages were collected; the entire third leaf at the V3 for M9 and the uppermost leaves at VT stage for SM9 were sampled 2 h after light exposure. The sampled leaves were immediately frozen in liquid nitrogen.

### Protein preparation

For each treatment, the equal weights of leaf samples from three individual plants were mixed and powdered by grinding in liquid nitrogen. For protein extraction, the powered leaf materials were digested for 5 min in a buffer at pH 8.5 containing 7 M urea, 2 M thiourea, 4% CHAPS, 40 mM Tris-HCl, 1 mM PMSF and 2 mM EDTA. After that, the DTT was added up to a final concentration of 10 mM. The resulting suspension was sonicated for 15 min at 200 W followed by centrifugation for 15 min at 30,000 *g* at 4°C. Then, a 5-fold volume of pre-cooled acetone containing 10% (v/v) TCA was mixed with the resulting supernatant followed by incubation overnight at −20°C. The incubated mix was then centrifuged for 15 min at 30,000 *g* at 4°C. The precipitate was washed three times with pre-cooled acetone, air-dried at room temperature, and then re-dissolved in a solution composed of 7 M urea, 2 M thiourea, 4% NP-40, and 20 mM Tris-HCl at pH 8.0–8.5. The solution was sonicated for 15 min at 200 W and centrifuged for 15 min at 30,000 *g* at 4°C. DTT was added to the supernatant to obtain a 10 mM solution, which was then incubated for 1 h at 56°C to reduce disulfide linkages in the proteins. Subsequently, 55 mM IAM (final concentration) was added and incubated for 1 h in the dark at room temperature followed by adding a 5-fold volume of pre-cooled acetone and incubating for 2 h at −20°C. The samples were centrifuged for 15 min at 30,000 *g* at 4°C. The pellet was air-dried for 5 min at room temperature, dissolved in 500 μL of 0.5 M TEAB (Applied Biosystems, Milan, Italy), and treated by sonication for 15 min at 200 W. The solution after sonication was centrifuged for 15 min at 30,000 *g* at 4°C. The supernatant was transferred to a tube and then assayed for concentrations and quality of proteins.

### Peptide labeling and strong cation exchange chromatography (SCX) fractionation

Protein sample of 100 μg was mixed with Trypsin Gold (Promega, Madison, WI, USA) at a ratio of 30 (protein):1(trypsin) and digested for 16 h at 37°C. The trypsin-hydrolysate peptide mixture was dried by vacuum centrifugation and reconstituted in 0.5 M TEAB for peptide labeling with the 8-plex iTRAQ reagent (Applied Biosystems) according to the manufacturer’s instructions (http://www.absciex.com.cn/Documents/Downloads/Literature/mass-spectrometry-4375249C) but with minor modifications.

Briefly, 1 U of the 8-plex iTRAQ reagent was thawed and reconstituted in 24 μL of isopropanol. The peptides were labeled with the isobaric tags and incubated for 2 h at room temperature. The labeled peptide mix was pooled, dried by vacuum centrifugation, and reconstituted in 4 mL buffer A at pH 2.7 containing 25 mM NaH_2_PO_4_ and 25% ACN. The mix was loaded onto a 4.6 × 250 mm Ultremex SCX column containing 5-μm particles (Phenomenex) equipped in an LC-20AB high-performance liquid chromatography (HPLC) pump system (Shimadzu, Kyoto, Japan). Gradient elution during HLPC was conducted for 10 min with buffer A, for 27 min with 5–60% buffer B (25 mM NaH_2_PO_4_, 1 M KCl in 25% ACN, pH 2.7), and then for 1 min with each concentration of 60 to 100% gradient buffer B, respectively, where a flow rate of elution was controlled at 1 mL/min. The last elution was performed with 100% buffer B. The eluant for each elution was collected every 1 min and monitored for contents of peptides by measuring the absorbance at a wavelength of 214 nm, The peptides collected in 20 fractions were desalted separately with a Strata-X C18 column (Phenomenex), and then vacuum-dried.

### HPLC coupled to Electrospray Ionization (ESI) Mass Spectrometry (MS)

The twenty vacuum-dried fractions were re-suspended in buffer A containing 5% ACN and 0.1% FA and centrifuged for 10 min at 20,000 *g*. The final concentration of the peptide in the supernatant was adjusted to 0.5 μg/μL. A 10-μL aliquot of supernatant was loaded by using an autosampler onto a 2-cm C18 trap column in an LC-20AD nanoHPLC (Shimadzu, Kyoto, Japan). The peptides were loaded onto a 10-cm analytical C18 column that was packed in-house and had an inner diameter of 75 μm, where loading was for 4 min at 8 μL/min. Elution was set at 300 nL/min starting from 2 to 35% buffer B containing 95% ACN and 0.1% FA then followed by elution for 5 min with 60% buffer B, for 2 min with 80% buffer B, for 4 min with 80% buffer B, and for 1 min with 5% buffer B, respectively. The whole process took about 35 min.

Data acquisition of peptide MS was performed on a TripleTOF 5600 System (AB SCIEX, Concord, ON, Canada) equipped with a Nanospray III source (AB SCIEX, Concord, ON, Canada) and a pulled quartz tip emitter (New Objectives, Woburn, MA, USA). The parameters for data acquisition were: 2.5 kV for ion spray voltage, 30 psi for the curtain gas, 15 psi for the nebulizer gas, and 150°C for an interface heater temperature. The mass spectrometer was operated for TOF MS scans under a reflection mode at a resolution ratio of ≥30,000. For IDA, when MS accumulated and reached 250, and only top 2^+^ to 5^+^ charge-state ions of >120 cps were then scanned. Total cycle time was 3.3 s. The Q2 transmission window was at an efficiency of 100% at 100 Da, and then run at a pulsing frequency of 11 kHz for each scanning and detected by suing a TDC detector of a 40-GHz detecting frequency. Four time-bins were summed and translated into the available data. A 35 ± 5 eV of sweeping collision energy setting was applied to all precursor ions for collision-induced dissociation. Dynamic exclusion was set for 1/2 of the peak width (15 s).

For each protein sample, the HLPC-MS analysis was technically repeated three times.

### Peptide analysis, protein identification and quantitation

Raw data files acquired from the Orbitrap were converted into MGF files using Proteome Discoverer 1.2 (PD 1.2, Thermo) [5600 msconverter], and blasted against the database containing *Z*. *mays* sequences (136770 sequences) to identify proteins as the 2^+^ and 3^+^ charge states of the peptides by using the Mascot 2.3.02 search engine (Matrix Science, London, UK). In Mascot 2.3.02, an automatic decoy database search was performed by choosing the decoy checkbox to both generate random peptide sequences and test raw spectra of the sequences against the real database. Additionally, only peptides with significance scores of ≥ 20 at the 99% confidence interval were counted as identified to reduce the probability of false identification. Each protein was identified by at least one unique peptide.

Each protein was quantified based on the intensity of reporter groups from at least two unique peptides, weighted and normalized by the median ratio in Mascot 2.3.02. Quantification of each protein was represented by the mean of signal intensity of reporter groups from three technical repeats of HPLC-MS analysis. The mean of the intensity of reporter groups for each protein was transformed by logarithm with 2 as the base, which was used for identifying differential proteins (DPs) in abundance between samples as a cut-off of a fold of > 1.2 with a *p*-value of < 0.05.

### Functional annotation and categorization of proteins

The proteins were annotated using the Gene Ontology (GO) categorization by using protein2go and go2protein programs against the non-redundant protein database (NR; NCBI; http://www.geneontology.org.). The Kyoto Encyclopedia of Genes and Genomes (KEGG) database (http://www.genome.jp/kegg/) and the Cluster of Orthologous Groups (COG) of proteins database (http://www.ncbi.nlm.nih.gov/COG/) were used to classify and group the identified proteins.

## Results

### Proteins identified in M9 and SM9 in response to different photoperiods

**Fig 1:**
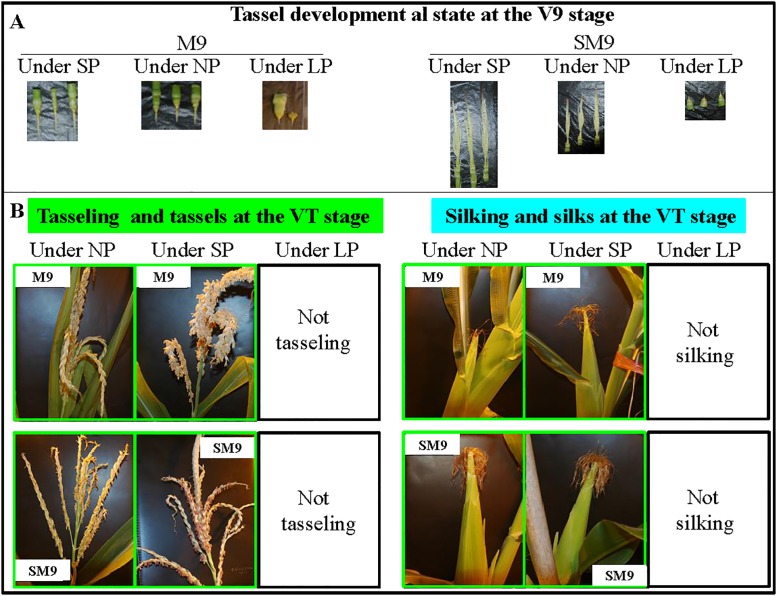
Tassel development al state at V9 stage (A) and, tasseling and silking of tropically-adapted and photoperiod-sensitive maize inbred lines M9 and SM9 (B) at VT stage under different photoperiods. LP, long photoperiod of 16 h light/8 h dark for a daily cycle; NP, neutral photoperiod of 12 h day light/12 h dark; SM9, Shuang M9; SP, short photoperiod of 10 h light/14 h dark; V3, vegetable 9 stage; VT, vegetable-tasseling stage.

**Fig 2:**
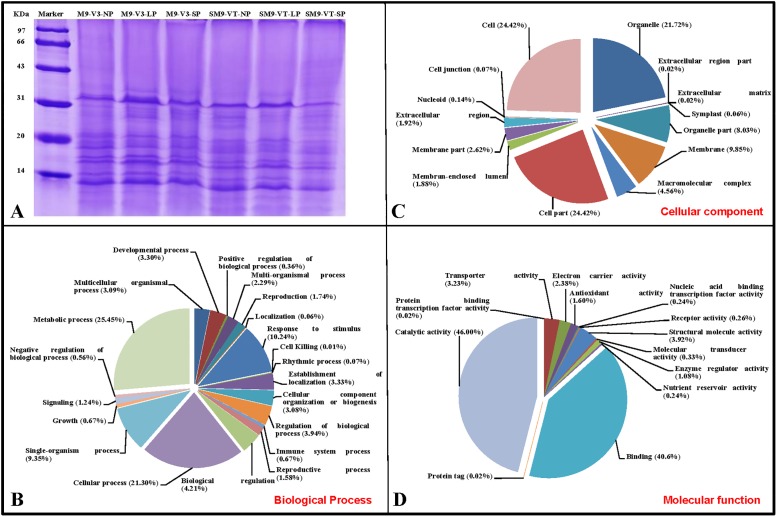
Extracted proteins, and GO-based categorization of biological processes and activities the identified proteins. Proteins from M9 leaves at V3 stage and from SM9 leaves at VT stage under LP, NP and SP (A). GO categorization (B, C, and D). GO, Gene Ontology; LP, long photoperiod of 16 h light/8 h dark for a daily cycle; NP, neutral photoperiod of 12 h day light/12 h dark; SM9, Shuang M9; SP, short photoperiod of 10 h light/14 h dark; V3, vegetable 3 stage; VT, vegetable-tasseling stage.

The two tropically-adapted and photoperiod-sensitive maize inbred lines, M9 and SM9, were characterized by the arrested development of the tassels at the V9 stage under an LP of 16 h light (Fig 1A), and failed to tassel and silk at the VT stage under an LP (Fig 1B). Because the qualities of the proteins extracted from M9 line at the VT stage and SM9 line at the V3 stage were poor, we sequenced six proteomes from leaf tissues of the M9 line at the V3 stage and the SM9 line at the VT stage grown under SP, NP, and LP (Fig 2A), respectively. The supplementary S1–S4 Figs respectively show the statistical information on the identified proteins, the distribution of protein mass in the protein profile, the distribution of the peptide segment length, and the percentage of the peptide of different lengths in the peptide repertoires. The number of peptides contained in the identified proteins and the matching error distribution of the peptides are shown in S5 and S6 Figs respectively. The distribution of abundance and differential abundance fold of proteins are shown in S7 Fig.

Consequently, a total of 4,395 proteins were identified (S1 Table; S1 Fig). The amino acid sequences of the identified proteins were listed in S2 Table.

### Prevalent categories of proteins based on GO

Regarding biological process by the Gene Ontology (GO) categorization of the entire proteome, we found two prevalent categories: metabolic process with 25.45% of and cellular process with 21.30% of the total proteins were found (Fig 2B).

Regarding the cellular component, there were the three most prevalent categories: the cell and cell part each comprised of 24.4% of the proteins while the organelle contained 21.72% of the proteins (Fig 2C). Regarding the molecular function, two dominant categories, catalytic activity (46.00% of the proteins) and binding (40.6% of the protein) were revealed (Fig 2D).

**Fig 3:**
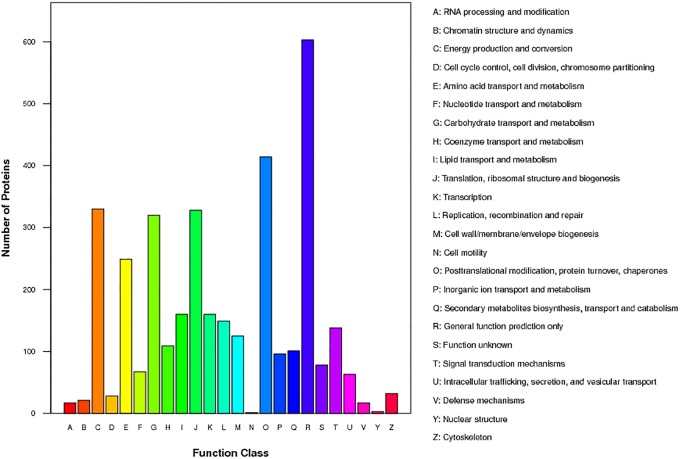
COG-based classification of functions and/or metabolism the identified proteins. The identified proteins were from M9 leaves at V3 stage and from SM9 leaves at the VT stage under LP, NP and SP. COG, Cluster of Orthologous Groups; LP, long photoperiod of 16 h light/8 h dark for a daily cycle; NP, neutral photoperiod of 12 h day light/12 h dark; SM9, Shuang M9; SP, short photoperiod of 10 h light/14 h dark; V3, vegetable 3 stage; VT, vegetable-tasseling stage.

Functional classification of the protein repertoires by the Cluster of Orthologous Groups (COG) indicated six largest functional groups: general function prediction; posttranslational modification, protein turnover, chaperons; energy production and conversion; translation, ribosomal structure and biogenesis; carbohydrate transport and metabolism; and amino acid transport and metabolism; and two smallest functional groups of nuclear structure and cell motility (Fig 3).

**Fig 4:**
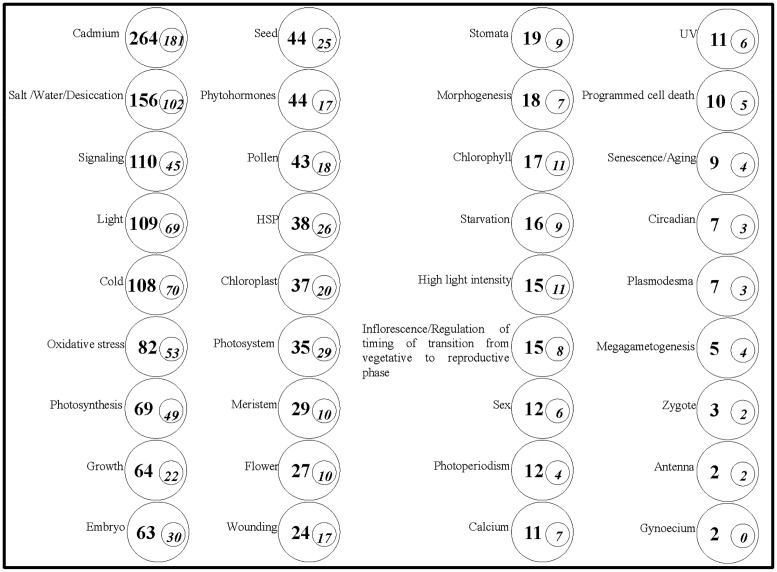
The number of proteins of interest and DPs in the protein repertoires from maize inbred lines M9 and SM9 under different photoperiods. The statistics were based on manual searching from GO categorization instead of simple annotation description of proteins in terms of the key items such as “photoperiodism”, “flower”, “cold”, and “salt/water/desiccation”. Some proteins involved multiple biological processes; therefore, the statistical figures may overlap. The boldface figures in the circles indicate the number of proteins under the items. The italics figures in the small circles represent the number of DPs. DPs, differential proteins in abundance; GO, Gene Ontology.

A manual search of GO categorization in the protein repertoires using the keywords the biotic and abiotic stresses, photoperiodism and circadian rhythm, growth and development, and flowering, indicated the major groups of the proteins associated with biological processes of cadmium (264 proteins); salt/water/desiccation (156 proteins); signaling (110 proteins); light (109 proteins); cold (108 proteins); oxidative stress (82 proteins); photosynthesis (69 proteins); growth (64 proteins); embryo (63 proteins); seed and hormones (44 proteins each) (Fig 4). Some of these proteins were found to be associated with multiple biological processes (S3 Table).

### DPs responding to photoperiod changes

We compared the relative abundance of the proteins in the same maize inbred line under different photoperiod conditions in LP vs. NP, and SP vs. NP (S1 Table). In the M9 leaves at the V3 stage, there were 401 DPs (9.1% of total protein number), in which 37 were co-regulated by LP and SP (S4 Table), 223 and 141 were only regulated by LP (S5 Table) and SP (S6 Table), respectively.

In the SM9 leaves at the VT stage, there were 425 DPs (9.7% of total proteins). In these proteins, 38 were co-regulated by LP and SP (S7 Table), whereas 254 and 133 were only regulated by LP (S8 Table) and SP (S9 Table), respectively.

### The photoperiod responsive and signaling DPs of interest

As shown in Fig 4, larger groups of DPs were those associated with cadmium stress (181 DPs), salt/water/desiccation stress (102 DPs), cold stress (70 DPs), oxidative stress (53 DPs), and wounding stress (17 DPs) (Fig 4).

Of the identified five classes of the photoperiod-related DPs, there were 69 light-responsive, 26 heat shock proteins (HSPs), 11 high light intensity-responsive, 6 UV-responsive, 4 photoperiodism processes-related, 3 circadian-responsive, and 2 antenna-related proteins (Fig 4).

There were 30 embryo-related, 25 seed-related, 18 pollen-related, 17 hormone-related, 10 flower-related, eight inflorescences/regulation of timing of the transition from vegetative to reproductive phase, 6 sex-related, and 4 megagametogenesis-related proteins (Fig 4).

Forty-five DPs were related to the signaling processes. The expression abundance of all these DPs was shown in S10 Table.

## Discussion

In the present study, we sequenced only six proteomes from three photoperiods of which three proteomes were from the M9 inbred line at the V3 stage and three from SM9 inbred line at the VT stage respectively. GO categorization can show more information on biological processes and intracellular location. Therefore, our analysis of the proteome-based responsive mechanisms under controlled photoperiod regimes, nutrient and moisture soils was based on GO categorization of some of the DPs.

The process of flowering is closely related to sex differentiation [17, 18]. We found that sex differentiation related DPs were mago nashi homologs; expansions B1, B3, B10, and B11; and pollen allergen Lol p 1. In these DPs, the maize pollen allergens function in pollen germination in the stigma [19] not in vegetative or female floral tissues [20]. The mago nashi protein functions in splicing and nuclear export of mRNAs [21]. The M9 and SM9 inbred lines usually flower at the VT stage under LP of 12 h light, but fail to grow out both tassels and silks at the VT stage under LP of 16 h light [8]. Interestingly, no DPs related to gynoecium development were found in either M9 or SM9 line (Fig 4; S10 Table). These results suggest that these DPs are closely involved in sex differentiation under photoperiodic effects. Moreover, failure in development and growth of the female silks and later silking under LP conditions are likely associated with the expression regulation of male differentiation-associated proteins because the empirical observation in the field is that tasseling is always before silking at a predictable time during maize growth.

The determination for flowering is also associated with regulation of transition from vegetative to reproductive phase (RTVR) [22–25]. Photoperiod pathway is one of the multiple genetic pathways involving multiple genes that control the floral transition in plants [22]. These pathways are coordinated at different levels [25]. We found 8 DPs related to RTVR (S10 Table). These results suggest that overly sensitive variations in the RTVR-related in response to photoperiod will not ensure a sharp transition from vegetative to floral stage under LP.

Maize inflorescence stems from the differentiation of primary shoot apical meristem (SAM) [26, 27]. In *Arabidopsis*, SERRATE is required for the development of early juvenile leaves, but it suppresses inflorescence development [28] by coordinately regulating SAM activity via a microRNA gene-silencing pathway [29]. Guanine nucleotide-binding proteins (known as G proteins) play important roles in the transmission of hormones and red and blue light-induced signals from membrane receptors to different effectors [30]. In SAM-related DPs (S10 Table), the expression of SERRATE RNA effectors were down-regulated in M9 under SP but did not significantly change in either M9 or SM9 under LP. The expression of the guanine nucleotide-binding protein subunit beta-like protein A was up-regulated in M9 under SP and down-regulated in SM9 under LP (S10 Table). Combined together, these findings clearly suggest that under LP the expression of SERRATE RNA effector molecules suppress late inflorescence development. Moreover, the suppressed expression of guanine nucleotide-binding protein subunit beta-like protein A not only impairs red and blue light signaling but also interferes with the flowering-required balance of phytohormones in maize.

Reportedly, the expression of catalase 1 persisted at a low level in a UV light-independent way during maize development [31]. Moreover, only catalase 3 expression was under the influence of a circadian rhythm in maize leaves [32]. In this study, a photoperiodism-related DP, catalase isozyme 1 (S10 Table) was found to respond to the abscisic acid stimulus signaling pathway (S3 Table) and was down-regulated under LP. These results suggest that catalase isozyme 1 is photoperiod-responsive, and its expression might be partially dependent on the intensity and the type of light. ZCN14 is the most favored candidate for possessing *FLOWERING LOCUS T* (*FT*) function [33], which is transported by plasmodesmata from the leaves into SAM to regulate flowering [34]. ZCN14/HEADING DATE 3B was up-regulated in the M9 line under LP and down-regulated in the SM9 line (S10 Table), suggesting that its expression varies with photoperiods depending on the inbred lines and tissues of maize. Photoperiodism should also be associated with plant responses to red, far-red light/blue, and UV-B light. These light species-responsive processes involved DPs are located in the inner cell wall, plasma membrane, chloroplast envelope, thylakoid, and nucleus (S10 Table). Some of these DPs were common players for responses to different light species (S3 Table). Among these DPs was that diphosphate kinase 2 was down-regulated (S10 Table), which responds to the auxin signaling pathway (S3 Table).

Mechanisms of circadian rhythm and photoperiodism are correlated but not exactly similar in plants [2]. The circadian rhythm-related DPs were distributed in ribosome, peroxisome, plastoglobule, vacuole, and chloroplast envelope, chloroplast, cell wall, plasma membrane, and nucleolus (S3 Table). Of these DPs, glycine-rich RNA-binding proteins were probably the linkages between circadian rhythm and photoperiodism by governing the export of photoperiodic mRNAs from the nucleus by abscisic acid signaling pathway (S3 Table).

We found three proteins that were sensitive to the absence of light or darkness. These proteins included the electron transfer flavoprotein-ubiquinone oxidoreductase located in the mitochondria; protein translocase subunits SECA1 and SECA located in chloroplast envelope, chloroplast stroma, and plasma membrane; and a glutamate dehydrogenase located in the mitochondria (S3 Table). These proteins are obviously different from light-responsive DPs, suggesting that dark signaling seems to be independent of the other light signal transmission routes. Our results showed that glutamate dehydrogenase was significantly up-regulated under SP but down-regulated under LP in M9 at the V3 stage. However, its expression was not significantly altered in SM9 at the VT stage with photoperiods (S3 Table). Therefore, the suppressed expression of glutamate dehydrogenase under LP probably elicits a unique photoperiod response, which interferes with the normal flowering process in both M9 and SM9 lines. These results clearly indicate that both light- and dark-responsive proteins in some maize lines have a combined effect on the photoperiodic response.

There were 11 DPs that sense high light intensity and are located in the inner cell wall/plasma membrane, chloroplast thylakoid membrane, and plastoglobule, cytosol, nucleus, plastid and mitochondrion (S3 and S10 Tables). These DPs differed from those that sense red, far-red, blue and UV-B light (S3 and S10 Tables), suggesting that the transmission of high-light density signals is clearly different from signaling pathways transmitting light species.

In the DPs related to the flowering processes (S3 Table), the 3'(2'),5'-bisphosphate nucleotidase was located in the chloroplast. This nucleotidase was found to be involved in the jasmonic acid- and abscisic acid-mediated signaling processes but negatively controlled signal transduction (S3 Table). Glutamate-cysteine ligase B was expressed in the chloroplast and responds to jasmonic acid stimuli (S3 Table). The poly(rC)-binding protein and alpha-galactosidase are known to positively regulate flower development, which was found to be up-regulated in both M9 and SM9 under LP but down-regulated in these two maize lines under SP. The expression of alpha-galactosidase was not altered in M9 with photoperiodic changes but down-regulated under SP and up-regulated under LP in SM9 (S10 Table). The actin-related protein 4, which involves the processes of long-day photoperiodism, flowering, and pollen sperm cell differentiation (S3 Table), was up-regulated only in SM9 under LP (S10 Table). Glucose-6-phosphate isomerase, which is known to positively regulate flower development, glycolysis, and gluconeogenesis (S3 Table), was down-regulated in both M9 and SM9 under LP (S10 Table). The copper transport protein ATOX1 involves DNA methylation and gene silencing (S3 Table) and was up-regulated in both M9 and SM9 under LP (S10 Table), suggesting that its role in enhancing the expression of some flowering-promoting genes, such as ATOX1 gene, is likely suppressed by methylation-induced gene silencing under LP. The findings of these DPs can also be suggestive of a signaling route for failure in flowering under LP.

The intercellular and supracellular communications in plants are accomplished by channels of plasmodesmata, which are associated with biological information vectors/signals such as phytohormones, reactive oxygen species (ROS), environmental stimuli [35], and photoperiod and light intensity [34]. The locations of plasmodesmata-related DPs were ascribed to the Golgi apparatus and the extracellular region, plasmodesmata, cell wall, envelope and stroma of chloroplasts, nucleolus, mitochondrion, cytosol, plasma membrane, vacuole, and cytoplasmic microtubule (S3 Table). The alpha-1,4-glucan-protein synthase and actin-7 with positive roles in transportation of plasmodesmata were down-regulated in both M9 and SM9 under LP (S10 Table), suggesting that the transport of the *FT*-like protein ZCN14 from the leaves into SAM through plasmodesmata was probably blocked under LP.

Long day and/or high light intensity can stimulate overproduction of ROS [36**–**38]. High levels of ROS can inhibit flowering [39], which mainly occurs in the chloroplast and mitochondrion of plants [40–42]. HSPs are very sensitive to extracellular stimuli [43]. The ROS-responding DPs found in this study included several HSPs and antioxidant peroxidase (S10 Table). The seed setting involves megagametogenesis and the development of the zygotes and embryos. Interestingly, many abiotic stresses-responsive DPs were observed in our study (Fig 4), which justify the photoperiod changes affecting stress tolerance in plants observed in previous studies [36, 37, 44]. DPs related to these processes included antioxidant enzyme proteins: L-ascorbate peroxidase 1 and superoxide dismutase [Mn] 3.4 (S10 Table). These DPs are distributed in the plant-type cell wall, cytosol, membrane/plasma membranes, vacuole, chloroplast, and mitochondrion (S10 Table). Therefore, the likely excessive ROS produced under LP damages various flowering processes by affecting megagametogenesis, zygotes and embryos.

All the processes discussed above are associated with one or more signaling pathways of phytohormones (S3 Table), suggesting that all photoperiodic effects on maize can be attributed to changes in the coordinated regulation of these hormones.

## Conclusions

**Fig 5:**
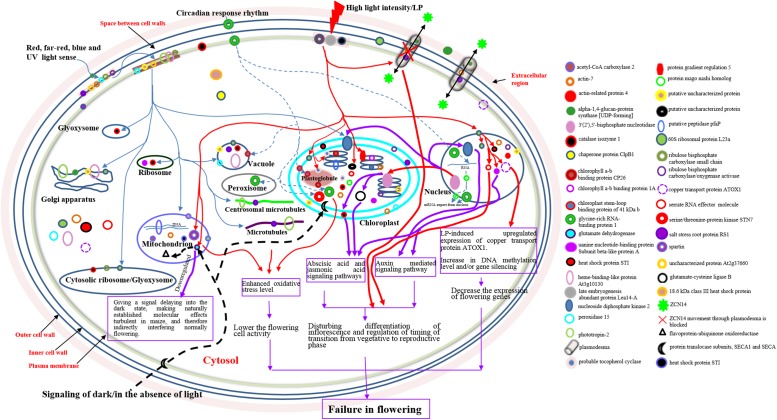
Proposed schematic hypothesis of maize response to photoperiodic changes. This schema is partly based on GO categorization of the identified DPs. The blue lines with arrowheads represent the sensing and signaling of red, far-red, blue and UV light. The dotted blue lines with arrowheads indicate the responses to circadian rhythm. The red lines with arrowheads mark responses to high light intensity and/or LP, relatively upregulated expression of the proteins, and the responding consequences under LP. The solid purple lines with arrowheads and long purple boxes mark the target proteins and routes, downregulated expression of the proteins under LP, or the responding consequences under LP. The thick dotted black line with arrowhead shows the target proteins and signaling of darkness/in the absence of light. *FT*-like ZCN14 movement is marked by black lines with double arrowheads. DPs, differential proteins in abundance; *FT*, *FLOWERING LOCUS T*; GO, Gene Ontology; LP, long photoperiod of 16 h light/8 h dark for a daily cycle.

Based on our findings and evidence from previous studies, we propose a model that outlines and shows protein roadmaps and echoing routes in responses of maize to photoperiod changes (Fig 5). Our model emphasizes: (1) the biological processes of photoperiodic flowering signaling, photoperiod response, circadian rhythm, and high light density response could crosstalk with each other thorough a group of light-sensing proteins that may be clustered in the cell wall and/or plasma membrane; (2) signals of high light density likely occur under LP transmit; (3) the cell-to-cell movement of *FT*-like ZCN14 from the leaves into SAM through plasmodesmata is blocked under LP; (4) signaling of darkness is mediated by glutamate dehydrogenase, apparently independent of the other light signal transmission routes; and (5) changes in expression of the mago nashi homolog and the glycine-rich RNA-binding protein with photoperiods make an impact on splicing and nuclear export of mRNAs, and/or for RNA/DNA secondary structure unwinding; therefore, controlling the response of RNA/DNA to photoperiod changes.

## Supporting information

S1 Table The annotation of finally identified and annotated proteins. (XLS)

S2 Table The amino acid sequences of the identified proteins. (XLS)

S3 Table Information on genes of interest. (XLS)

S4 Table DPs co-regulated in M9 by short and long photoperiods. (XLS)

S5 Table DPs specific to long photoperiod in M9. (XLS)

S6 Table DPs specific to short photoperiod in M9. (XLS)

S7 Table DPs co-regulated in SM9 by short and long photoperiods. (XLS)

S8 Table DPs specific to long photoperiod in SM9. (XLS)

S9 Table DPs specific to short photoperiod in SM9. (XLS)

S10 Table Expression of interesting proteins in maize inbred lines M9 and SM9 under different photoperiods. (DOC)

S1 Fig The basic statistics information of protein identification. (TIFF)

S2 Fig The distribution of protein mass in the protein profile. (TIFF)

S3 Fig The peptide segment length distribution. (TIFF)

S4 Fig The percentage of peptide of different lengths in the peptide repertoires. (TIFF)

S5 Fig The number of peptides contained in identified proteins. (TIFF)

S6 Fig Matching error distribution of the peptides. (TIFF)

S7 Fig The distribution of abundance, and differential expression fold of proteins. (TIFF)
