# Supplementary material for: A photoperiod-responsive protein compendium and conceptual proteome roadmap outline in maize grown in growth chambers with controlled conditions
Source: PLoS One. 2017 Apr 11;12(4):e0174003. doi: 10.1371/journal.pone.0174003 (PMC5388471; doi:10.1371/journal.pone.0174003)
Supplement: S10 Table — (DOC) [file pone.0174003.s010.doc]

**S10 Table** Expression of interesting proteins in maize inbred lines M9 and SM9 under different photoperiods

Up-regulation and down-regulation were marked in red and in green, respectively. LP, long photoperiod; NP, neutral photoperiod; SP, short photoperiod;

| **Uniprot_Tremble Accession** | **Uniprot_Swissprot or Uniprot _Tremble Description** | | **KEGG pathway(ko_id)** | | **Ratio in M9** | | | | | | | | | |  | | | **Ratio in SM9** | | | | | | | | | | | | |  | | |
| --- | --- | --- | --- | --- | --- | --- | --- | --- | --- | --- | --- | --- | --- | --- | --- | --- | --- | --- | --- | --- | --- | --- | --- | --- | --- | --- | --- | --- | --- | --- | --- | --- | --- |
|  |  |  |  |  | **SP vs. NP** | |  | | | **LP vs. NP** | |  | **LP vs. SP** | | |  | | | **SP vs. NP** | |  | | **LP vs. NP** | |  | | **LP vs. SP** | | | | |  | |
| **UV** | |  |  | |  | |  | | |  | |  |  | | |  | | |  | |  | |  | |  | |  | | | | |  | |
| B4G0H7 | | Heme-binding-like protein At3g10130, chloroplastic | - | | 0.371 | |  | | | 0.84 | |  | 2.563 | | |  | | | 1 | |  | | 1.166 | |  | | 1.397 | | | | |  | |
| B6TXN0 | | DNA-damage-repair/toleration protein DRT102 | - | | 1.514 | |  | | | 1.11 | |  | 0.842 | | |  | | | 1 | |  | | 1.135 | |  | | 1.132 | | | | |  | |
| B4FTT2 | | Putative uncharacterized protein | K11493|regulator of chromosome condensation!  K10615| ubiquitin-protein ligase HERC4 [EC:6.3.2.19] | | 1.739 | |  | | | 1.89 | |  | 1.151 | | |  | | | 0.8 | |  | | 1.296 | |  | | 1.588 | | | | |  | |
| B6T5B9 | | 60S ribosomal protein L10 | K02866 | | 1.133 | |  | | | 2.11 | |  | 1.547 | | |  | | | 1.2 | |  | | 1.324 | |  | | 1.193 | | | | |  | |
| B4G1Z4 | | Metacaspase-9 | - | | 0.821 | |  | | | 1.44 | |  | 1.471 | | |  | | | 1 | |  | | 1.033 | |  | | 1.002 | | | | |  | |
| B6SZ65 | | Anthocyanidin 3-O-glucosyltransferase 2 | K13496|UDP-glucosyl transferase 73C [EC:2.4.1.-] | | 1.139 | |  | | | 0.65 | |  | 0.57 | | |  | | | 0.8 | |  | | 1.062 | |  | | 0.818 | | | | |  | |
| **Photoperiodism** | | | | |  | |  | | |  | |  |  | | |  | | |  | |  | |  | |  | |  | | | | |  | |
| C0PES7 | | 3'(2'),5'-bisphosphate nucleotidase | K15422|[EC:3.1.3.7 3.1.3.57] | | 1.033 | |  | | | 1.02 | |  | 0.888 | | |  | | | 1.4 | |  | | 1.631 | |  | | 1.184 | | | | |  | |
| A8WES5 | | ZCN14/Protein HEADING DATE 3B | - | | 1.116 | |  | | | 1.16 | |  | 1.075 | | |  | | | 0.7 | |  | | 3.363 | |  | | 4.911 | | | | |  | |
| B6UHU1 | | Catalase isozyme 1 | K03781| [EC:1.11.1.6] | | 1.535 | |  | | | 1.19 | |  | 0.639 | | |  | | | 0.9 | |  | | 0.788 | |  | | 0.895 | | | | |  | |
| B7ZZT1 | | Actin-related protein 4 | K11652 | | 1.577 | |  | | | 1.28 | |  | 0.797 | | |  | | | 0.8 | |  | | 1.368 | |  | | 1.762 | | | | |  | |
| **Circadian** | |  |  | |  | |  | | |  | |  |  | | |  | | |  | |  | |  | |  | |  | | | | |  | |
| B4FEH8 | | Chloroplast stem-loop binding protein of 41 kDa b, chloroplastic | K01710|dTDP-glucose 4,6-dehydratase [EC:4.2.1.46] | | 1.165 | |  | | | 0.95 | |  | 0.851 | | |  | | | 0.6 | |  | | 0.975 | |  | | 1.66 | | | | |  | |
| B4FVB8 | | Serine/threonine-protein kinase STN7, chloroplastic | K08282|non-specific serine/threonine protein kinase [EC:2.7.11.1]!K00924| [EC:2.7.1.-]!  K03097|casein kinase II subunit alpha [EC:2.7.11.1] | | 2.335 | |  | | | 1.07 | |  | 0.441 | | |  | | | 1 | |  | | 0.729 | |  | | 0.68 | | | | |  | |
| B8A3G8 | | Glycine-rich RNA-binding protein 1 (Fragment) | K13195|cold-inducible RNA-binding protein!  K02965|small subunit ribosomal protein S19!  K11294|nucleolin!  K12741|heterogeneous nuclear ribonucleoprotein A1/A3 | | 0.623 | |  | | | 0.87 | |  | 1.341 | | |  | | | 1.2 | |  | | 0.915 | |  | | 0.777 | | | | |  | |
| **High light intensity** | | |  | |  | |  | | |  | |  |  | | |  | | |  | |  | |  | |  | |  | | | | |  | |
| C0HHY8 | | Probable tocopherol cyclase, chloroplastic | K09834 | | 1.238 | |  | | | 1.33 | |  | 1.008 | | |  | | | 0.7 | |  | | 0.666 | |  | | 0.927 | | | | |  | |
| C0P732 | | Heat shock protein STI | K09553|stress-induced-phosphoprotein 1 | | 0.347 | |  | | | 1 | |  | 2.617 | | |  | | | 0.8 | |  | | 1.234 | |  | | 1.51 | | | | |  | |
| B4FZV0 | | Putative uncharacterized protein | - | | 0.809 | |  | | | 0.79 | |  | 0.83 | | |  | | | 0.8 | |  | | 1.383 | |  | | 1.761 | | | | |  | |
| B6U0M5 | | Protein PROTON GRADIENT REGULATION 5, chloroplastic | - | | 0.647 | |  | | | 0.67 | |  | 1.025 | | |  | | | 1.3 | |  | | 0.607 | |  | | 0.448 | | | | |  | |
| B4FQT3 | | Heat shock protein STI | K09553|stress-induced-phosphoprotein 1 | | 0.524 | |  | | | 1.36 | |  | 2.965 | | |  | | | 1.1 | |  | | 0.924 | |  | | 0.969 | | | | |  | |
| O81229 | | 60S ribosomal protein L23a | K02893 | | 0.385 | |  | | | 1.38 | |  | 4.001 | | |  | | | 2.2 | |  | | 1.625 | |  | | 0.843 | | | | |  | |
| B4FCF6 | | Late embryogenesis abundant protein Lea14-A | - | | 2.801 | |  | | | 1.18 | |  | 0.43 | | |  | | | 0.6 | |  | | 1.709 | |  | | 2.691 | | | | |  | |
| C0PDC7 | | Chaperone protein ClpB1 | K03695|ATP-dependent Clp protease ATP-binding subunit ClpB | | 0.784 | |  | | | 0.95 | |  | 1.704 | | |  | | | 0.4 | |  | | 0.655 | |  | | 1.301 | | | | |  | |
| B4F9E8 | | 18.6 kDa class III heat shock protein | K13993|HSP20 family protein | | 0.745 | |  | | | 1.5 | |  | 1.856 | | |  | | | 0.7 | |  | | 1.522 | |  | | 1.838 | | | | |  | |
| C4J4W3 | | Heat shock protein STI | K09553|stress-induced-phosphoprotein 1 | | 0.389 | |  | | | 1.1 | |  | 2.419 | | |  | | | 1.2 | |  | | 1.403 | |  | | 1.317 | | | | |  | |
| B4F8T3 | | Spartin | - | | 1.414 | |  | | | 0.97 | |  | 0.758 | | |  | | | 1.1 | |  | | 0.798 | |  | | 0.826 | | | | |  | |
| **Response to absence of light/dark** | | | | |  | |  | | |  | |  |  | | |  | | |  | |  | |  | |  | |  | | | | |  | |
| B8A3K4 | | Electron transfer flavoprotein-ubiquinone oxidoreductase | K00311|sbi:SORBI_01g017140| [EC:1.5.5.1] | | 1.217 | |  | | | 1.088 | |  | 0.941 | | |  | | | 0.711 | |  | | 0.843 | |  | | 0.998 | | | | |  | |
| C5XIW8 | | Protein translocase subunit SECA1 | K03070|sbi:SORBI_03g013090 | | --- | |  | | | --- | |  | --- | | |  | | | --- | |  | | --- | |  | | --- | | | | |  | |
| C5XIW8 | | Protein translocase subunit SecA | K03070|sbi:SORBI_03g013090 | | 1.266 | |  | | | 0.724 | |  | 0.563 | | |  | | | 0.941 | |  | | 1.106 | |  | | 1.195 | | | | |  | |
| B4F9J5 | | Glutamate dehydrogenase | K00261| [EC:1.4.1.3] | | 1.431 | |  | | | 0.655 | |  | 0.38 | | |  | | | 0.944 | |  | | 0.957 | |  | | 0.91 | | | | |  | |
| **Light** | |  |  | |  | |  | | |  | |  |  | | |  | | |  | |  | |  | |  | |  | | | | |  | |
| B4F8T3 | | Spartin | - | | 1.414 | |  | | | 0.97 | |  | 0.758 | | |  | | | 1.1 | |  | | 0.798 | |  | | 0.826 | | | | |  | |
| B4FCF6 | | Late embryogenesis abundant protein Lea14-A | - | | 2.801 | |  | | | 1.18 | |  | 0.43 | | |  | | | 0.6 | |  | | 1.709 | |  | | 2.691 | | | | |  | |
| B4G0H7 | | Heme-binding-like protein At3g10130, chloroplastic | - | | 0.371 | |  | | | 0.84 | |  | 2.563 | | |  | | | 1 | |  | | 1.166 | |  | | 1.397 | | | | |  | |
| C0P732 | | Heat shock protein STI | K09553|stress-induced-phosphoprotein 1 | | 0.347 | |  | | | 1 | |  | 2.617 | | |  | | | 0.8 | |  | | 1.234 | |  | | 1.51 | | | | |  | |
| C4J4W3 | | Heat shock protein STI | K09553|stress-induced-phosphoprotein 1 | | 0.389 | |  | | | 1.1 | |  | 2.419 | | |  | | | 1.2 | |  | | 1.403 | |  | | 1.317 | | | | |  | |
| B4FQT3 | | Heat shock protein STI | K09553|stress-induced-phosphoprotein 1 | | 0.524 | |  | | | 1.36 | |  | 2.965 | | |  | | | 1.1 | |  | | 0.924 | |  | | 0.969 | | | | |  | |
| B6TKL9 | | Chlorophyll a-b binding protein, chloroplastic | K08908|light-harvesting complex I chlorophyll a/b binding protein 2 | | 1.002 | |  | | | 0.45 | |  | 0.446 | | |  | | | 1.4 | |  | | 1.008 | |  | | 0.763 | | | | |  | |
| C0P520 | | Chlorophyll a-b binding protein, chloroplastic | K08908|light-harvesting complex I chlorophyll a/b binding protein 2 | | 1.202 | |  | | | 0.45 | |  | 0.374 | | |  | | | 1.3 | |  | | 0.689 | |  | | 0.531 | | | | |  | |
| O81608 | | Chlorophyll a-b binding protein, chloroplastic | K08913|light-harvesting complex II chlorophyll a/b binding protein 2 | | 0.785 | |  | | | 0.77 | |  | 0.935 | | |  | | | 1 | |  | | 0.998 | |  | | 1.034 | | | | |  | |
| B4FV94 | | Chlorophyll a-b binding protein P4, chloroplastic | K08910|light-harvesting complex I chlorophyll a/b binding protein 4 | | 1.089 | |  | | | 0.73 | |  | 0.59 | | |  | | | 1.3 | |  | | 0.631 | |  | | 0.489 | | | | |  | |
| Q41746 | | Chlorophyll a-b binding protein CP26, chloroplastic | K08916|light-harvesting complex II chlorophyll a/b binding protein 5 | | 0.987 | |  | | | 0.71 | |  | 0.694 | | |  | | | 1.1 | |  | | 0.538 | |  | | 0.478 | | | | |  | |
| B4FXB0 | | Chlorophyll a-b binding protein CP24 10B, chloroplastic | K08917|light-harvesting complex II chlorophyll a/b binding protein 6 | | 0.906 | |  | | | 0.54 | |  | 0.609 | | |  | | | 0.9 | |  | | 0.976 | |  | | 1.087 | | | | |  | |
| B6TRW9 | | Chlorophyll a-b binding protein CP24 10B, chloroplastic | K08917|light-harvesting complex II chlorophyll a/b binding protein 6 | | 0.741 | |  | | | 0.62 | |  | 0.807 | | |  | | | 1.2 | |  | | 0.644 | |  | | 0.527 | | | | |  | |
| B4FNR1 | | Chlorophyll a-b binding protein 1, chloroplastic | K08912|light-harvesting complex II chlorophyll a/b binding protein 1 | | 0.72 | |  | | | 0.6 | |  | 0.772 | | |  | | | 1 | |  | | 0.649 | |  | | 0.668 | | | | |  | |
| B4F9E8 | | 18.6 kDa class III heat shock protein | K13993|HSP20 family protein | | 0.745 | |  | | | 1.5 | |  | 1.856 | | |  | | | 0.7 | |  | | 1.522 | |  | | 1.838 | | | | |  | |
| B6T7T3 | | MAIZE OHP2 | - | | 0.645 | |  | | | 0.71 | |  | 1.097 | | |  | | | 0.8 | |  | | 0.775 | |  | | 0.917 | | | | |  | |
| B4FV78 | | Acyl carrier protein, chloroplastic | K03955|NADH dehydrogenase (ubiquinone) 1 alpha/beta subcomplex 1 [EC:1.6.5.3 1.6.99.3] | | 0.816 | |  | | | 1 | |  | 1.209 | | |  | | | 1 | |  | | 1.081 | |  | | 1.09 | | | | |  | |
| B8A3B5 | | Ferric reduction oxidase 6 | K13447|respiratory burst oxidase [EC:1.6.3.- 1.11.1.-] | | 0.861 | |  | | | 0.84 | |  | 0.963 | | |  | | | 1.5 | |  | | 1.701 | |  | | 1.121 | | | | |  | |
| O81229 | | 60S ribosomal protein L23a | K02893 | | 0.385 | |  | | | 1.38 | |  | 4.001 | | |  | | | 2.2 | |  | | 1.625 | |  | | 0.843 | | | | |  | |
| C0PDG8 | | Putative peptidase pfaP | K04773|protease IV [EC:3.4.21.-] | | 1.027 | |  | | | 0.84 | |  | 0.959 | | |  | | | 0.9 | |  | | 0.715 | |  | | 0.76 | | | | |  | |
| B4G1T3 | | Bifunctional chitinase/lysozyme (Fragment) | K01183|chitinase [EC:3.2.1.14] | | 0.689 | |  | | | 0.53 | |  | 0.904 | | |  | | | 1.1 | |  | | 0.27 | |  | | 0.186 | | | | |  | |
| B6TVA3 | | Bifunctional chitinase/lysozyme (Fragment) | K01183|chitinase [EC:3.2.1.14] | | 0.937 | |  | | | 0.67 | |  | 0.967 | | |  | | | 0.7 | |  | | 0.483 | |  | | 0.781 | | | | |  | |
| Q38JE3 | | Outer membrane lipoprotein blc | K03098 | | 1.528 | |  | | | 2.49 | |  | 1.602 | | |  | | | 0.6 | |  | | 0.839 | |  | | 1.386 | | | | |  | |
| C5XIY9 | | Dihydrolipoyl dehydrogenase 1, mitochondrial | K00382|dihydrolipoamide dehydrogenase [EC:1.8.1.4] | | 1.492 | |  | | | 1.11 | |  | 0.823 | | |  | | | 0.9 | |  | | 1.104 | |  | | 1.335 | | | | |  | |
| C4J5G3 | | Dihydrolipoyl dehydrogenase 1, mitochondrial | K00382| [EC:1.8.1.4] | | 1.374 | |  | | | 1.06 | |  | 0.783 | | |  | | | 0.8 | |  | | 0.779 | |  | | 1.094 | | | | |  | |
| B7ZYT6 | | Ribulose bisphosphate carboxylase small chain, chloroplastic | K01602|[EC:4.1.1.39] | | 1.021 | |  | | | 0.8 | |  | 0.874 | | |  | | | 0.9 | |  | | 0.543 | |  | | 0.682 | | | | |  | |
| B4FSJ3 | | Ribulose bisphosphate carboxylase small chain, chloroplastic | K01602|[EC:4.1.1.39] | | 1.084 | |  | | | 0.72 | |  | 0.659 | | |  | | | 1.2 | |  | | 1.224 | |  | | 1.12 | | | | |  | |
| B4FVB8 | | Serine/threonine-protein kinase STN7, chloroplastic | K08282|non-specific serine/threonine protein kinase [EC:2.7.11.1]!  K00924| [EC:2.7.1.-]!  K03097|casein kinase II subunit alpha [EC:2.7.11.1] | | 2.335 | |  | | | 1.07 | |  | 0.441 | | |  | | | 1 | |  | | 0.729 | |  | | 0.68 | | | | |  | |
| C0PDC7 | | Chaperone protein ClpB1 | K03695|ATP-dependent Clp protease ATP-binding subunit ClpB | | 0.784 | |  | | | 0.95 | |  | 1.704 | | |  | | | 0.4 | |  | | 0.655 | |  | | 1.301 | | | | |  | |
| B6TJC5 | | Developmentally regulated G-protein 2 | K06944 | | 1.809 | |  | | | 1.03 | |  | 0.608 | | |  | | | 0.8 | |  | | 0.864 | |  | | 1.052 | | | | |  | |
| B4FHT1 | | Annexin D7 | - | | 1.529 | |  | | | 1.16 | |  | 0.744 | | |  | | | 1.2 | |  | | 2.162 | |  | | 2.035 | | | | |  | |
| Q43863 | | Annexin D7 | - | | 1.354 | |  | | | 1.4 | |  | 0.83 | | |  | | | 0.9 | |  | | 1.361 | |  | | 1.175 | | | | |  | |
| B4FZV0 | | MAIZE Putative uncharacterized protein | - | | 0.809 | |  | | | 0.79 | |  | 0.83 | | |  | | | 0.8 | |  | | 1.383 | |  | | 1.761 | | | | |  | |
| B4FUH1 | | NADH dehydrogenase [ubiquinone] iron-sulfur protein 8-B, mitochondrial | K03941|[EC:1.6.5.3 1.6.99.3] | | 2.253 | |  | | | 1.07 | |  | 0.466 | | |  | | | 0.8 | |  | | 0.952 | |  | | 1.178 | | | | |  | |
| E9KJH4 | | Cytochrome b559 subunit alpha | K02707|photosystem II cytochrome b559 subunit alpha | | 1.409 | |  | | | 0.75 | |  | 0.545 | | |  | | | 1.3 | |  | | 0.625 | |  | | 0.503 | | | | |  | |
| B4FQ80 | | Chlorophyll a-b binding protein, chloroplastic | K08912|light-harvesting complex II chlorophyll a/b binding protein 1 | | 0.558 | |  | | | 0.49 | |  | 0.886 | | |  | | | 0.9 | |  | | 0.917 | |  | | 1.085 | | | | |  | |
| B4FUA1 | | Chlorophyll a-b binding protein M9, chloroplastic | K08912|light-harvesting complex II chlorophyll a/b binding protein 1 | | 0.572 | |  | | | 0.54 | |  | 0.957 | | |  | | | 0.9 | |  | | 0.791 | |  | | 0.838 | | | | |  | |
| C0P9F5 | | Chlorophyll a-b binding protein 1B-21, chloroplastic | K08907|light-harvesting complex I chlorophyll a/b binding protein 1 | | 1.177 | |  | | | 0.57 | |  | 0.481 | | |  | | | 1.3 | |  | | 0.534 | |  | | 0.528 | | | | |  | |
| B4FZL4 | | Chlorophyll a-b binding protein 1B-21, chloroplastic | K08907|light-harvesting complex I chlorophyll a/b binding protein 1 | | 0.767 | |  | | | 0.68 | |  | 0.868 | | |  | | | 0.9 | |  | | 0.286 | |  | | 0.358 | | | | |  | |
| B6SZ37 | | Chlorophyll a-b binding protein 1A, chloroplastic (Fragments) | K08911|light-harvesting complex I chlorophyll a/b binding protein 5 | | 0.74 | |  | | | 0.59 | |  | 0.801 | | |  | | | 1 | |  | | 0.532 | |  | | 0.538 | | | | |  | |
| B7ZYW4 | | Glutamate-1-semialdehyde 2,1-aminomutase, chloroplastic | K01845|[EC:5.4.3.8] | | 1.352 | |  | | | 1.2 | |  | 0.857 | | |  | | | 0.8 | |  | | 1.296 | |  | | 1.738 | | | | |  | |
| C0P5X1 | | Ribulose bisphosphate carboxylase/oxygenase activase, chloroplastic | - | | 0.904 | |  | | | 0.84 | |  | 0.89 | | |  | | | 0.8 | |  | | 1.299 | |  | | 1.617 | | | | |  | |
| C0P5X6 | | Ribulose bisphosphate carboxylase/oxygenase activase, chloroplastic | K03064|26S proteasome regulatory subunit T4 | | 0.734 | |  | | | 0.9 | |  | 1.047 | | |  | | | 0.3 | |  | | 0.854 | |  | | 3.115 | | | | |  | |
| B1PEY4 | | Superoxide dismutase [Cu-Zn], chloroplastic | K04565| [EC:1.15.1.1] | | 0.649 | |  | | | 1.02 | |  | 1.544 | | |  | | | 1.2 | |  | | 1.134 | |  | | 1.005 | | | | |  | |
| B6SP61 | | Ferredoxin-1, chloroplastic | K02639 | | 0.639 | |  | | | 0.61 | |  | 1.029 | | |  | | | 1.6 | |  | | 1.761 | |  | | 1.089 | | | | |  | |
| C8XU20 | | NAD(P)H-quinone oxidoreductase subunit J, chloroplastic | K05581|[EC:1.6.5.3] | | 1.524 | |  | | | 0.56 | |  | 0.385 | | |  | | | 0.9 | |  | | 0.565 | |  | | 0.67 | | | | |  | |
| C5XLG2 | | NAD(P)H-quinone oxidoreductase subunit K, chloroplastic | K05582| K [EC:1.6.5.3] | | 1.185 | |  | | | 0.62 | |  | 0.583 | | |  | | | 1 | |  | | 0.779 | |  | | 0.807 | | | | |  | |
| G3EFI2 | | NAD(P)H-quinone oxidoreductase subunit I, chloroplastic | K05580| [EC:1.6.5.3] | | 1.266 | |  | | | 0.62 | |  | 0.473 | | |  | | | 1 | |  | | 0.65 | |  | | 0.673 | | | | |  | |
| C5IHC9 | | Pyruvate, phosphate dikinase 2 (Fragment) | K01006| [EC:2.7.9.1] | | 0.98 | |  | | | 0.7 | |  | 0.769 | | |  | | | 1.1 | |  | | 0.707 | |  | | 0.687 | | | | |  | |
| B7ZYP6 | | Pyruvate, phosphate dikinase 1, chloroplastic | K01006| [EC:2.7.9.1] | | 1.089 | |  | | | 0.7 | |  | 0.635 | | |  | | | 1.2 | |  | | 0.835 | |  | | 0.714 | | | | |  | |
| C5IHE6 | | Pyruvate, phosphate dikinase 1, chloroplastic | K01006| [EC:2.7.9.1] | | 0.75 | |  | | | 0.81 | |  | 1.142 | | |  | | | 1 | |  | | 0.78 | |  | | 0.812 | | | | |  | |
| C0P4V6 | | Nitrate reductase [NADH] (Fragment) | K00360| [EC:1.7.1.1]!  K00387|sulfite oxidase [EC:1.8.3.1] | | 1.809 | |  | | | 0.97 | |  | 0.565 | | |  | | | 0.9 | |  | | 0.442 | |  | | 0.48 | | | | |  | |
| B4F8L7 | | Glyceraldehyde-3-phosphate dehydrogenase B, chloroplastic (Fragment) | K05298|[EC:1.2.1.13] | | 0.796 | |  | | | 0.76 | |  | 0.938 | | |  | | | 1.1 | |  | | 0.696 | |  | | 0.635 | | | | |  | |
| B4FYG3 | | Succinate-semialdehyde dehydrogenase, mitochondrial | K00135| [EC:1.2.1.16] | | 1.857 | |  | | | 1 | |  | 0.58 | | |  | | | 0.9 | |  | | 1.602 | |  | | 1.992 | | | | |  | |
| C8XU84 | | NAD(P)H-quinone oxidoreductase subunit H, chloroplastic | K05579|[EC:1.6.5.3] | | 1.437 | |  | | | 0.73 | |  | 0.477 | | |  | | | 1.1 | |  | | 0.991 | |  | | 0.821 | | | | |  | |
| Q7DNB2 | | 30S ribosomal protein S15, chloroplastic | K05579|NAD(P)H-quinone oxidoreductase subunit H [EC:1.6.5.3] | | 0.393 | |  | | | 1.04 | |  | 2.587 | | |  | | | 1.5 | |  | | 1.102 | |  | | 0.814 | | | | |  | |
| C0HHY8 | | Probable tocopherol cyclase, chloroplastic | K09834|tocopherol cyclase | | 1.238 | |  | | | 1.33 | |  | 1.008 | | |  | | | 0.7 | |  | | 0.666 | |  | | 0.927 | | | | |  | |
| B6U0M5 | | Protein PROTON GRADIENT REGULATION 5, chloroplastic | - | | 0.647 | |  | | | 0.67 | |  | 1.025 | | |  | | | 1.3 | |  | | 0.607 | |  | | 0.448 | | | | |  | |
| B4G0R1 | | Nucleoside diphosphate kinase 2, chloroplastic | K00940|[EC:2.7.4.6] | | 0.89 | |  | | | 0.92 | |  | 0.911 | | |  | | | 0.8 | |  | | 0.835 | |  | | 0.947 | | | | |  | |
| C4J9M7 | | 2-Cys peroxiredoxin BAS1, chloroplastic | K03386|peroxiredoxin (alkyl hydroperoxide reductase subunit C) [EC:1.11.1.15] | | 0.831 | |  | | | 0.64 | |  | 0.738 | | |  | | | 1 | |  | | 1.766 | |  | | 1.74 | | | | |  | |
| B4FUT1 | | Peroxidase 15 | K00430|[EC:1.11.1.7] | | 0.824 | |  | | | 1.48 | |  | 1.806 | | |  | | | 0.8 | |  | | 1.107 | |  | | 1.444 | | | | |  | |
| B4FRH8 | | Actin-7 | K10355  K05692 | | 0.926 | |  | | | 1.12 | |  | 1.218 | | |  | | | 0.8 | |  | | 0.349 | |  | | 0.433 | | | | |  | |
| B6SS20 | | Phototropin-2 | K08282|non-specific serine/threonine protein kinase [EC:2.7.11.1] | | 0.786 | |  | | | 0.93 | |  | 1.311 | | |  | | | 1.1 | |  | | 1.62 | |  | | 1.775 | | | | |  | |
| B4FRI7 | | Peptidyl-prolyl cis-trans isomerase CYP20-3, chloroplastic | K03768|peptidyl-prolyl cis-trans isomerase B (cyclophilin B) [EC:5.2.1.8] | | 0.645 | |  | | | 1.3 | |  | 1.945 | | |  | | | 0.8 | |  | | 0.794 | |  | | 0.901 | | | | |  | |
| B4FH62 | | Uncharacterized protein At2g37660, chloroplastic | - | | 0.539 | |  | | | 0.86 | |  | 1.556 | | |  | | | 0.8 | |  | | 0.944 | |  | | 1.178 | | | | |  | |
| C5X468 | | Ferredoxin-dependent glutamate synthase, chloroplastic | K00284| [EC:1.4.7.1] | | 1.115 | |  | | | 0.75 | |  | 0.671 | | |  | | | 1.5 | |  | | 1.139 | |  | | 0.746 | | | | |  | |
| B4FKV6 | | Peroxidase 15 | K00430|[EC:1.11.1.7] | | 0.585 | |  | | | 1.58 | |  | 2.769 | | |  | | | 0.6 | |  | | 1.341 | |  | | 1.982 | | | | |  | |
| B6TXY3 | | Quinone oxidoreductase-like protein At1g23740, chloroplastic | K07119  K00344|NADPH2:quinone reductase [EC:1.6.5.5] | | 0.803 | |  | | | 0.88 | |  | 1.02 | | |  | | | 0.9 | |  | | 1.038 | |  | | 1.177 | | | | |  | |
| B4F9J5 | | Glutamate dehydrogenase | K00261|(NAD(P)+) [EC:1.4.1.3] | | 1.431 | |  | | | 0.66 | |  | 0.38 | | |  | | | 0.9 | |  | | 0.957 | |  | | 0.91 | | | | |  | |
| **Antenna** | |  |  | |  | |  | | |  | |  |  | | |  | | |  | |  | |  | |  | |  | | | | |  | |
| Q41746 | | Chlorophyll a-b binding protein CP26, chloroplastic | K08916|light-harvesting complex II chlorophyll a/b binding protein 5 | | 0.987 | |  | | | 0.71 | |  | 0.694 | | |  | | | 1.1 | |  | | 0.538 | |  | | 0.478 | | | | |  | |
| B6SZ37 | | Chlorophyll a-b binding protein 1A, chloroplastic (Fragments) | K08911|light-harvesting complex I chlorophyll a/b binding protein 5 | | 0.74 | |  | | | 0.59 | |  | 0.801 | | |  | | | 1 | |  | | 0.532 | |  | | 0.538 | | | | |  | |
| **Flower** | |  |  | |  | |  | | |  | |  |  | | |  | | |  | |  | |  | |  | |  | | | | |  | |
| B6SPL7 | | Copper transport protein ATOX1 | K07213 | | 0.887 | |  | | | 1.43 | |  | 1.622 | | |  | | | 1.1 | |  | | 1.294 | |  | | 1.19 | | | | |  | |
| Q8W4W3 | | Glutamate--cysteine ligase B, chloroplastic | K01919|[EC:6.3.2.2] | | 1.586 | |  | | | 0.9 | |  | 0.53 | | |  | | | 1.1 | |  | | 1.362 | |  | | 1.087 | | | | |  | |
| B4G1X1 | | Alpha-galactosidase | K07407|[EC:3.2.1.22] | | 1.002 | |  | | | 0.75 | |  | 0.785 | | |  | | | 0.8 | |  | | 1.191 | |  | | 1.392 | | | | |  | |
| C3UZ63 | | Heat shock protein 81-1 | K04079|molecular chaperone HtpG | | 1.188 | |  | | | 1.04 | |  | 0.973 | | |  | | | 0.9 | |  | | 0.633 | |  | | 0.835 | | | | |  | |
| C0PES7 | | 3'(2'),5'-bisphosphate nucleotidase | K15422|inositol polyphosphate 1-phosphatase [EC:3.1.3.7 3.1.3.57] | | 1.033 | |  | | | 1.02 | |  | 0.888 | | |  | | | 1.4 | |  | | 1.631 | |  | | 1.184 | | | | |  | |
| C5X3T6 | | Heat shock protein 81-3 | K04079|molecular chaperone HtpG | | 2.086 | |  | | | 1.19 | |  | 0.525 | | |  | | | 0.9 | |  | | 0.834 | |  | | 1.043 | | | | |  | |
| B8A170 | | Poly(rC)-binding protein 3 | K13162 | | 0.501 | |  | | | 1.16 | |  | 2.319 | | |  | | | 0.9 | |  | | 1.231 | |  | | 1.605 | | | | |  | |
| C0PAU7 | | Glucose-6-phosphate isomerase | K01810|[EC:5.3.1.9] | | 1.482 | |  | | | 0.94 | |  | 0.699 | | |  | | | 1.3 | |  | | 0.696 | |  | | 0.741 | | | | |  | |
| B7ZXN1 | | Ferritin-1, chloroplastic | K00522|ferritin heavy chain [EC:1.16.3.1] | | 1.801 | |  | | | 0.78 | |  | 0.495 | | |  | | | 1.1 | |  | | 0.855 | |  | | 0.807 | | | | |  | |
| B7ZZT1 | | Actin-related protein 4 | K11652|actin-like protein 6B | | 1.577 | |  | | | 1.28 | |  | 0.797 | | |  | | | 0.8 | |  | | 1.368 | |  | | 1.762 | | | | |  | |
| **Inflorescence/ regulation of timing of transition from vegetative to reproductive phase** | | | | | | | | | | | | | | | | | | | | | | | | | | | | |  | | | | |
| B6TNC0 | | Ankyrin repeat domain-containing protein 2 | | K06694|26S proteasome non-ATPase regulatory subunit 10!  K12489|Arf-GAP with coiled-coil, ANK repeat and PH domain-containing protein | 0.343 | |  | | | 1.16 | |  | 4.291 | | |  | | | 1.2 | |  | | 0.577 | |  | | | 0.641 | | | |  | |
| C5YXI1 | | Uncharacterized protein in xynA 3'region (Fragment) | | - | 0.802 | |  | | | 1.05 | |  | 0.886 | | |  | | | 1.2 | |  | | 0.624 | |  | | | 0.464 | | | |  | |
| B4FP39 | | ATP-dependent Clp protease proteolytic subunit 4, chloroplastic | | K01358|[EC:3.4.21.92] | 0.93 | |  | | | 0.8 | |  | 1.016 | | |  | | | 1 | |  | | 1.345 | |  | | | 1.312 | | | |  | |
| C5WU06 | | FACT complex subunit SPT16 | | K01262|Xaa-Pro aminopeptidase [EC:3.4.11.9] | 1.463 | |  | | | 1.45 | |  | 0.975 | | |  | | | 0.8 | |  | | 0.958 | |  | | | 1.146 | | | |  | |
| C0PJP0 | | Cullin-associated NEDD8-dissociated protein 1 | | - | 2.467 | |  | | | 1.14 | |  | 0.557 | | |  | | | 0.8 | |  | | 1.019 | |  | | | 1.115 | | | |  | |
| A8WES5 | | Protein HEADING DATE 3B | | - | 1.116 | |  | | | 1.16 | |  | 1.075 | | |  | | | 0.7 | |  | | 3.363 | |  | | | 4.911 | | | |  | |
| B8A3G8 | | Glycine-rich RNA-binding protein 1 (Fragment) | | K13195|cold-inducible RNA-binding protein!  K02965|small subunit ribosomal protein S19!  K11294|nucleolin!  K12741|heterogeneous nuclear ribonucleoprotein A1/A3 | 0.623 | |  | | | 0.87 | |  | 1.341 | | |  | | | 1.2 | |  | | 0.915 | |  | | | 0.777 | | | |  | |
| B6TP60 | | Glycine-rich protein 2 | | K09250|cellular nucleic acid-binding protein | 1.264 | |  | | | 1.15 | |  | 0.925 | | |  | | | 1.4 | |  | | 1.211 | |  | | | 1.067 | | | |  | |
| **Primary shoot apical meristem specification** | | | | | | | | |  | | |  |  | | |  | | |  | |  | |  | |  | | |  | | | |  | |
| B7ZXU2 | | Serrate RNA effector molecule | | - | 0.517 | |  | | | 1.76 | |  | 3.35 | | |  | | | 1.1 | |  | | 1.081 | |  | | | 0.998 | | | |  | |
| Q08367 | | Acetyl-CoA carboxylase 2 | | K11262|acetyl-CoA carboxylase / biotin carboxylase [EC:6.4.1.2 6.3.4.14] | 1.555 | |  | | | 0.96 | |  | 0.64 | | |  | | | 1.2 | |  | | 1.581 | |  | | | 1.319 | | | |  | |
| B6SJ21 | | Guanine nucleotide-binding protein subunit beta-like protein A | | K14753|guanine nucleotide-binding protein subunit beta-2-like 1 protein | 1.817 | |  | | | 1.48 | |  | 0.8 | | |  | | | 0.8 | |  | | 0.781 | |  | | | 0.94 | | | |  | |
| **Meristem** | |  | |  |  | |  | | |  | |  |  | | |  | | |  | |  | |  | |  | | |  | | | |  | |
| C5WU06 | | FACT complex subunit SPT16 | | K01262|Xaa-Pro aminopeptidase [EC:3.4.11.9] | 1.463 | |  | | | 1.45 | |  | 0.975 | | |  | | | 0.8 | |  | | 0.958 | |  | | | 1.146 | | | |  | |
| C0PJP0 | | Cullin-associated NEDD8-dissociated protein 1 | | - | 2.467 | |  | | | 1.14 | |  | 0.557 | | |  | | | 0.8 | |  | | 1.019 | |  | | | 1.115 | | | |  | |
| B6TNC0 | | Ankyrin repeat domain-containing protein 2 | | K06694|26S proteasome non-ATPase regulatory subunit 10!  K12489|Arf-GAP with coiled-coil, ANK repeat and PH domain-containing protein | 0.343 | |  | | | 1.16 | |  | 4.291 | | |  | | | 1.2 | |  | | 0.577 | |  | | | 0.641 | | | |  | |
| B6TP60 | | Glycine-rich protein 2 | | K09250|cellular nucleic acid-binding protein | 1.264 | |  | | | 1.15 | |  | 0.925 | | |  | | | 1.4 | |  | | 1.211 | |  | | | 1.067 | | | |  | |
| C5YXI1 | | Uncharacterized protein in xynA 3'region (Fragment) | | - | 0.802 | |  | | | 1.05 | |  | 0.886 | | |  | | | 1.2 | |  | | 0.624 | |  | | | 0.464 | | | |  | |
| C0PMY0 | | Cell division protein FtsP | | - | 0.924 | |  | | | 1.02 | |  | 1.04 | | |  | | | 0.9 | |  | | 1.184 | |  | | | 1.21 | | | |  | |
| B8A3G8 | | Glycine-rich RNA-binding protein 1 (Fragment) | | K13195|cold-inducible RNA-binding protein!  K02965|small subunit ribosomal protein S19!  K11294|nucleolin!  K12741|heterogeneous nuclear ribonucleoprotein A1/A3 | 0.623 | |  | | | 0.87 | |  | 1.341 | | |  | | | 1.2 | |  | | 0.915 | |  | | | 0.777 | | | |  | |
| **Megagametogenesis** | | | | | | | | | | | | | | | | | | | | | | | | | | | | |  | | | | |
| B4FIY0 | | Probable serine protease EDA2 | | K01285|lysosomal Pro-X carboxypeptidase [EC:3.4.16.2] | 0.862 | |  | | | 0.82 | |  | 0.96 | | |  | | | 0.7 | |  | | 0.76 | |  | | | 1.056 | | | |  | |
| B8A0F2 | | Probable serine protease EDA2 | | K01285|lysosomal Pro-X carboxypeptidase [EC:3.4.16.2] | 1.252 | |  | | | 0.84 | |  | 0.657 | | |  | | | 1.2 | |  | | 1.882 | |  | | | 1.571 | | | |  | |
| C0PLN9 | | D-3-phosphoglycerate dehydrogenase, chloroplastic | | K00058| [EC:1.1.1.95] | 2.558 | |  | | | 1.09 | |  | 0.494 | | |  | | | 0.9 | |  | | 1.243 | |  | | | 1.06 | | | |  | |
| C4J316 | | D-3-phosphoglycerate dehydrogenase, chloroplastic | | K00058| [EC:1.1.1.95] | 1.898 | |  | | | 1.5 | |  | 0.931 | | |  | | | 0.7 | |  | | 0.907 | |  | | | 1.32 | | | |  | |
| **Zygote** | |  | |  |  | |  | | |  | |  |  | | |  | | |  | |  | |  | |  | | |  | | | |  | |
| Q5EUD7 | | Protein disulfide isomerase-like 2-2 | | K01829| [EC:5.3.4.1]  K09584| [EC:5.3.4.1] | 1.114 | |  | | | 1.54 | |  | 1.232 | | |  | | | 1 | |  | | 1.526 | |  | | | 1.644 | | | |  | |
| C4J5B2 | | Oxysterol-binding protein-related protein 3A | | - | 1.337 | |  | | | 0.96 | |  | 0.686 | | |  | | | 1 | |  | | 0.882 | |  | | | 0.793 | | | |  | |
| **Pollen** | |  | |  |  | |  | | |  | |  |  | | |  | | |  | |  | |  | |  | | |  | | | |  | |
| C5YLJ8 | | Protein mago nashi homolog | | K12877 | 1.298 | |  | | | 1.17 | |  | 0.957 | | |  | | | 1 | |  | | 1.6 | |  | | | 1.288 | | | |  | |
| B7ZZT1 | | Actin-related protein 4 | | K11652 | 1.577 | |  | | | 1.28 | |  | 0.797 | | |  | | | 0.8 | |  | | 1.368 | |  | | | 1.762 | | | |  | |
| B6TKK2 | | Probable carboxylesterase 18 | | K14493|  gibberellin receptor GID1 [EC:3.-.-.-] | 0.988 | |  | | | 0.92 | |  | 1.065 | | |  | | | 1 | |  | | 0.471 | |  | | | 0.504 | | | |  | |
| B6TXN5 | | Probable carboxylesterase 18 | | K14493|gibberellin receptor GID1 [EC:3.-.-.-] | 1.641 | |  | | | 1.35 | |  | 0.812 | | |  | | | --- | |  | | --- | |  | | | 0.345 | | | |  | |
| C0PDA6 | | Fumarate hydratase 1, mitochondrial | | K01679| [EC:4.2.1.2] | 1.335 | |  | | | 1.07 | |  | 0.78 | | |  | | | 0.8 | |  | | 1.225 | |  | | | 1.359 | | | |  | |
| B4FBF4 | | Serine hydroxymethyl transferase 1 | | K00600| [EC:2.1.2.1] | 1.771 | |  | | | 1.19 | |  | 0.603 | | |  | | | 1.2 | |  | | 0.871 | |  | | | 0.711 | | | |  | |
| C0P869 | | Dihydroxy-acid dehydratase | | K01687| [EC:4.2.1.9] | 1.549 | |  | | | 1.3 | |  | 0.805 | | |  | | | 0.8 | |  | | 1.381 | |  | | | 1.425 | | | |  | |
| B4FR08 | | Cysteine synthase, chloroplastic/chromoplastic | | K01738| [EC:2.5.1.47] | 0.83 | |  | | | 1.13 | |  | 1.387 | | |  | | | 0.8 | |  | | 0.92 | |  | | | 1.13 | | | |  | |
| B8A367 | | Cysteine synthase, chloroplastic/chromoplastic | | K01738| [EC:2.5.1.47] | 0.826 | |  | | | 1.12 | |  | 1.13 | | |  | | | 1 | |  | | 0.646 | |  | | | 0.693 | | | |  | |
| C0PKG2 | | UDP-sugar pyrophosphorylase | | K12447| [EC:2.7.7.64] | 0.852 | |  | | | 0.94 | |  | 0.859 | | |  | | | 0.9 | |  | | 1.127 | |  | | | 1.312 | | | |  | |
| Q9ZR86 | | Probable nucleoredoxin 1-1 | | - | 1.506 | |  | | | 0.92 | |  | 0.579 | | |  | | | 0.9 | |  | | 0.909 | |  | | | 1.002 | | | |  | |
| B4FAD9 | | UTP--glucose-1-phosphate uridylyltransferase | | K00963| [EC:2.7.7.9] | 1.663 | |  | | | 0.92 | |  | 0.575 | | |  | | | 1 | |  | | 1.466 | |  | | | 1.58 | | | |  | |
| B4FBJ7 | | 26S protease regulatory subunit 6A homolog | | K03065 | 1.655 | |  | | | 1.51 | |  | 0.946 | | |  | | | 1 | |  | | 1.337 | |  | | | 1.327 | | | |  | |
| C0PHC0 | | V-type proton ATPase catalytic subunit A (Fragment) | | K02145| [EC:3.6.3.14] | 1.32 | |  | | | 1.08 | |  | 0.806 | | |  | | | 1.1 | |  | | 1.666 | |  | | | 1.48 | | | |  | |
| C0PHC0 | | V-type proton ATPase catalytic subunit A (Fragment) | | K02145|subunit A [EC:3.6.3.14] | 1.909 | |  | | | 1.02 | |  | 0.578 | | |  | | | 0.9 | |  | | 1.245 | |  | | | 1.35 | | | |  | |
| B4FGL3 | | Probable ATP synthase 24 kDa subunit, mitochondrial | | - | 1.151 | |  | | | 1.08 | |  | 0.896 | | |  | | | 1 | |  | | 1.462 | |  | | | 1.297 | | | |  | |
| B6TDF8 | | Glyceraldehyde-3-phosphate dehydrogenase (Fragment) | | K00134| [EC:1.2.1.12] | 1.529 | |  | | | 1.41 | |  | 0.906 | | |  | | | 0.9 | |  | | 1.347 | |  | | | 1.525 | | | |  | |
| F2CS21 | | Calmodulin-1 | | K02183 | 0.396 | |  | | | 1.2 | |  | 2.892 | | |  | | | 1 | |  | | 1.305 | |  | | | 1.154 | | | |  | |
| **Sex** | |  | |  |  | |  | | |  | |  |  | | |  | | |  | |  | |  | |  | | |  | | | |  | |
| C5YLJ8 | | Protein mago nashi homolog | | K12877 mago nashi | 1.298 | |  | | | 1.17 | |  | 0.957 | | |  | | | 1 | |  | | 1.6 | |  | | | 1.288 | | | |  | |
| B6TUA7 | | Pollen allergen Lol p 1 | | - | 20.835 | |  | | | 92.2 | |  | 6.676 | | |  | | | 0.2 | |  | | 2.07 | |  | | | 9.604 | | | |  | |
| 1Q94KS8 | | Expansin-B3 | | - | 2.89 | |  | | | 2.62 | |  | 0.894 | | |  | | | 1.3 | |  | | 1.516 | |  | | | 1.16 | | | |  | |
| C0PAE9 | | Expansin-B11 | | - | 5.399 | |  | | | 6.27 | |  | 1.144 | | |  | | | 0.3 | |  | | 1.202 | |  | | | 3.527 | | | |  | |
| B4FIV7 | | Expansin-B10 | | - | 10.088 | |  | | | 10.9 | |  | 1.067 | | |  | | | 0.2 | |  | | 1.836 | |  | | | 7.423 | | | |  | |
| B4FXT7 | | Expansin-B1 | | - | 7.802 | |  | | | 11.2 | |  | 1.407 | | |  | | | 0.3 | |  | | 1.744 | |  | | | 7.041 | | | |  | |
| **Seed** | |  | |  |  | |  | | |  | |  |  | | |  | | |  | |  | |  | |  | | |  | | | |  | |
| C5YLJ8 | | Protein mago nashi homolog | | K12877 | 1.298 | |  | | | 1.17 | |  | 0.957 | | |  | | | 1 | |  | | 1.6 | |  | | | 1.288 | | | |  | |
| C5XQU8 | | Protein ABCI7, chloroplastic | | K09015|Fe-S cluster assembly protein SufD | 0.76 | |  | | | 0.9 | |  | 1.053 | | |  | | | 0.8 | |  | | 0.815 | |  | | | 1.099 | | | |  | |
| B4FS63 | | Probable 26S proteasome non-ATPase regulatory subunit 7 | | K03038 | 1.777 | |  | | | 1.25 | |  | 0.69 | | |  | | | 0.8 | |  | | 1.429 | |  | | | 1.788 | | | |  | |
| B4FAF5 | | Outer envelope protein 80, chloroplastic | | K07277 | 0.712 | |  | | | 0.69 | |  | 0.985 | | |  | | | 1.1 | |  | | 1.041 | |  | | | 0.949 | | | |  | |
| B4FQI3 | | NADH dehydrogenase [ubiquinone] 1 alpha subcomplex subunit 13-B | | K11353 [EC:1.6.5.3 1.6.99.3] | 1.183 | |  | | | 0.95 | |  | 0.502 | | |  | | | 0.8 | |  | | 0.846 | |  | | | 1.035 | | | |  | |
| B7ZY61 | | DNA damage-binding protein 1a | | K10610 | 2.77 | |  | | | 0.93 | |  | 0.322 | | |  | | | 1 | |  | | 1.493 | |  | | | 1.668 | | | |  | |
| B4FWY6 | | Tyrosine--tRNA ligase | | K01866| [EC:6.1.1.1] | 1.3 | |  | | | 0.85 | |  | 0.75 | | |  | | | 1.1 | |  | | 1.35 | |  | | | 1.235 | | | |  | |
| B6TP60 | | Glycine-rich protein 2 | | K09250|cellular nucleic acid-binding protein | 1.264 | |  | | | 1.15 | |  | 0.925 | | |  | | | 1.4 | |  | | 1.211 | |  | | | 1.067 | | | |  | |
| C5YCZ2 | | Elongation factor G, chloroplastic | | K02355 | 1.183 | |  | | | 0.85 | |  | 0.638 | | |  | | | 1 | |  | | 1.024 | |  | | | 0.933 | | | |  | |
| B4FQM2 | | Pyrophosphate--fructose 6-phosphate 1-phosphotransferase subunit beta | | K00895 [EC:2.7.1.90] | 3.061 | |  | | | 1.88 | |  | 0.604 | | |  | | | 1.1 | |  | | 2.023 | |  | | | 1.839 | | | |  | |
| B4FS18 | | L-ascorbate peroxidase 1, cytosolic | | K00434| [EC:1.11.1.11] | 1.354 | |  | | | 3.06 | |  | 2.304 | | |  | | | 0.8 | |  | | 0.933 | |  | | | 1.227 | | | |  | |
| B6TM55 | | L-ascorbate peroxidase 1, cytosolic | | K00434| [EC:1.11.1.11] | 0.862 | |  | | | 0.69 | |  | 0.731 | | |  | | | 0.9 | |  | | 0.944 | |  | | | 1.123 | | | |  | |
| B4FN58 | | Protein-L-isoaspartate O-methyltransferase | | K00573 [EC:2.1.1.77] | 1.307 | |  | | | 2.09 | |  | 1.702 | | |  | | | 1.1 | |  | | 1.715 | |  | | | 1.519 | | | |  | |
| C0HG50 | | Proline--tRNA ligase | | K01881| [EC:6.1.1.15] | 0.842 | |  | | | 0.78 | |  | 0.943 | | |  | | | 0.5 | |  | | 0.614 | |  | | | 1.252 | | | |  | |
| B4F9H6 | | Superoxide dismutase [Mn] 3.4, mitochondrial | | K04564|[EC:1.15.1.1] | 1.445 | |  | | | 0.87 | |  | 0.583 | | |  | | | 1 | |  | | 2.062 | |  | | | 2.083 | | | |  | |
| Q8L5H0 | | Sucrose synthase 3 | | K00695| [EC:2.4.1.13] | 2.07 | |  | | | 0.91 | |  | 0.404 | | |  | | | 1.1 | |  | | 1.435 | |  | | | 1.387 | | | |  | |
| B8A2P6 | | Tudor domain-containing protein 1 | | K15979 | 1.177 | |  | | | 1.02 | |  | 0.927 | | |  | | | 0.8 | |  | | 0.645 | |  | | | 0.86 | | | |  | |
| B8A2P6 | | Tudor domain-containing protein 1 | | K15979 | 1.564 | |  | | | 1.22 | |  | 0.705 | | |  | | | 0.8 | |  | | 0.538 | |  | | | 1.005 | | | |  | |
| B6TDJ2 | | Bifunctional aspartate aminotransferase and glutamate/aspartate-prephenate aminotransferase | | K15849|[EC:2.6.1.1 2.6.1.78 2.6.1.79] | 1.707 | |  | | | 1.19 | |  | 0.683 | | |  | | | 1.1 | |  | | 0.966 | |  | | | 0.881 | | | |  | |
| C0P3Z4 | | Subtilisin-like protease | | - | 1.004 | |  | | | 1.66 | |  | 1.712 | | |  | | | 1.1 | |  | | 1.169 | |  | | | 1.268 | | | |  | |
| B4G0E0 | | Acyl-coenzyme A oxidase 4, peroxisomal | | K00232|[EC:1.3.3.6] | 1.5 | |  | | | 0.81 | |  | 0.53 | | |  | | | 1.2 | |  | | 0.971 | |  | | | 0.869 | | | |  | |
| C5Z483 | | Reticuline oxidase-like protein | | - | 1.361 | |  | | | 1.07 | |  | 0.77 | | |  | | | 0.8 | |  | | 0.304 | |  | | | 0.411 | | | |  | |
| C0PHW4 | | Putative uncharacterized protein | | - | 1.589 | |  | | | 1.2 | |  | 0.767 | | |  | | | 1.2 | |  | | 1 | |  | | | 0.898 | | | |  | |
| B6T5D7 | | Reticuline oxidase | | - | 6.676 | |  | | | 5.64 | |  | 1.414 | | |  | | | 0.5 | |  | | 0.979 | |  | | | 1.556 | | | |  | |
| B4F8B9 | | Alcohol dehydrogenase class-3 | | K00121|S-(hydroxymethyl)glutathione dehydrogenase [EC:1.1.1.284 1.1.1.1] | 1.848 | |  | | | 1.19 | |  | 0.589 | | |  | | | 0.9 | |  | | 1.377 | |  | | | 1.418 | | | |  | |
| **Embryo** | |  | |  |  | |  | | |  | |  |  | | |  | | |  | |  | |  | |  | | |  | | | |  | |
| C5YLJ8 | | Protein mago nashi homolog | | K12877 | 1.298 | |  | | | 1.167 | |  | 0.957 | | |  | | | 1 | |  | | 1.6 | |  | | | 1.288 | | | |  | |
| C5XQU8 | | Protein ABCI7, chloroplastic | | K09015|Fe-S cluster assembly protein SufD | 0.76 | |  | | | 0.9 | |  | 1.053 | | |  | | | 0.8 | |  | | 0.815 | |  | | | 1.099 | | | |  | |
| B4FS63 | | Probable 26S proteasome non-ATPase regulatory subunit 7 | | K03038 | 1.777 | |  | | | 1.246 | |  | 0.69 | | |  | | | 0.8 | |  | | 1.429 | |  | | | 1.788 | | | |  | |
| B4FAF5 | | Outer envelope protein 80, chloroplastic | | K07277 | 0.712 | |  | | | 0.693 | |  | 0.985 | | |  | | | 1.1 | |  | | 1.041 | |  | | | 0.949 | | | |  | |
| B4FQI3 | | NADH dehydrogenase [ubiquinone] 1 alpha subcomplex subunit 13-B | | K11353 [EC:1.6.5.3 1.6.99.3] | 1.183 | |  | | | 0.948 | |  | 0.502 | | |  | | | 0.8 | |  | | 0.846 | |  | | | 1.035 | | | |  | |
| Q9FQB7 | | Glutathione S-transferase U22 | | K00799 [EC:2.5.1.18] | 4.327 | |  | | | 1.168 | |  | 0.345 | | |  | | | 4.6 | |  | | 2.232 | |  | | | 0.367 | | | |  | |
| B4FRE3 | | Ankyrin repeat domain-containing protein 2 | | K06694|26S proteasome non-ATPase regulatory subunit 10 | 0.286 | |  | | | 1.162 | |  | 4.085 | | |  | | | 1.1 | |  | | 1.578 | |  | | | 1.464 | | | |  | |
| B7ZY61 | | DNA damage-binding protein 1a | | K10610 | 2.77 | |  | | | 0.931 | |  | 0.322 | | |  | | | 1 | |  | | 1.493 | |  | | | 1.668 | | | |  | |
| B4FWY6 | | Tyrosine--tRNA ligase | | K01866 [EC:6.1.1.1] | 1.3 | |  | | | 0.851 | |  | 0.75 | | |  | | | 1.1 | |  | | 1.35 | |  | | | 1.235 | | | |  | |
| B6SIS5 | | 60S ribosomal protein L10a-3 | | K02865 | 1.949 | |  | | | 1.694 | |  | 1.152 | | |  | | | 1.3 | |  | | 1.303 | |  | | | 0.891 | | | |  | |
| B4FQM2 | | Pyrophosphate--fructose 6-phosphate 1-phosphotransferase subunit beta | | K00895 [EC:2.7.1.90] | 3.061 | |  | | | 1.878 | |  | 0.604 | | |  | | | 1.1 | |  | | 2.023 | |  | | | 1.839 | | | |  | |
| B6U577 | | Glutathione S-transferase U9 | | K00799 [EC:2.5.1.18] | 1.38 | |  | | | 1.483 | |  | 1.317 | | |  | | | 9.4 | |  | | 3.39 | |  | | | 0.473 | | | |  | |
| B4FTS6 | | Endochitinase A | | K01183 [EC:3.2.1.14] | 5.624 | |  | | | 2.756 | |  | 0.437 | | |  | | | 0.9 | |  | | 3.797 | |  | | | 4.62 | | | |  | |
| B4FS18 | | L-ascorbate peroxidase 1, cytosolic | | K00434|[EC:1.11.1.11] | 1.354 | |  | | | 3.062 | |  | 2.304 | | |  | | | 0.8 | |  | | 0.933 | |  | | | 1.227 | | | |  | |
| B6TM55 | | L-ascorbate peroxidase 1, cytosolic | | K00434| [EC:1.11.1.11] | 0.862 | |  | | | 0.687 | |  | 0.731 | | |  | | | 0.9 | |  | | 0.944 | |  | | | 1.123 | | | |  | |
| C0HG50 | | Proline--tRNA ligase | | K01881| [EC:6.1.1.15] | 0.842 | |  | | | 0.78 | |  | 0.943 | | |  | | | 0.5 | |  | | 0.614 | |  | | | 1.252 | | | |  | |
| B4FLS7 | | Ribosome-recycling factor, chloroplastic | | K02838 | 0.178 | |  | | | 0.86 | |  | 4.955 | | |  | | | 1.1 | |  | | 0.954 | |  | | | 0.886 | | | |  | |
| C0PLX3 | | Ribosome-recycling factor, chloroplastic | | K02838 | 0.18 | |  | | | 0.976 | |  | 4.67 | | |  | | | 0.9 | |  | | 0.851 | |  | | | 0.795 | | | |  | |
| B4F9H6 | | Superoxide dismutase [Mn] 3.4, mitochondrial | | K04564|[EC:1.15.1.1] | 1.445 | |  | | | 0.869 | |  | 0.583 | | |  | | | 1 | |  | | 2.062 | |  | | | 2.083 | | | |  | |
| B4FIY7 | | NEDD8-conjugating enzyme Ubc12 | | K10579|ubiquitin-conjugating enzyme E2 M [EC:6.3.2.19] | 1.135 | |  | | | 1.27 | |  | 1.246 | | |  | | | 1.1 | |  | | 1.221 | |  | | | 1.235 | | | |  | |
| B7ZZZ2 | | RuBisCO large subunit-binding protein subunit alpha, chloroplastic (Fragment) | | K04077|chaperonin GroEL | 0.953 | |  | | | 0.888 | |  | 0.876 | | |  | | | 0.9 | |  | | 1.174 | |  | | | 1.446 | | | |  | |
| Q2QU06 | | RuBisCO large subunit-binding protein subunit alpha, chloroplastic (Fragment) | | K04077|chaperonin GroEL | 0.9 | |  | | | 0.662 | |  | 0.724 | | |  | | | 0.8 | |  | | 1.045 | |  | | | 1.372 | | | |  | |
| C5YW53 | | RuBisCO large subunit-binding protein subunit alpha, chloroplastic (Fragment) | | K04077|chaperonin GroEL | 0.971 | |  | | | 0.916 | |  | 0.966 | | |  | | | 0.9 | |  | | 2.101 | |  | | | 2.272 | | | |  | |
| B6TT00 | | Chitinase 6 | | K01183| [EC:3.2.1.14] | 1.726 | |  | | | 1.315 | |  | 0.67 | | |  | | | 0.8 | |  | | 0.509 | |  | | | 0.689 | | | |  | |
| B4FBJ7 | | 26S protease regulatory subunit 6A homolog | | K03065 | 1.655 | |  | | | 1.508 | |  | 0.946 | | |  | | | 1 | |  | | 1.337 | |  | | | 1.327 | | | |  | |
| B6TDJ2 | | Bifunctional aspartate aminotransferase and glutamate/aspartate-prephenate aminotransferase | | K15849| [EC:2.6.1.1 2.6.1.78 2.6.1.79] | 1.707 | |  | | | 1.189 | |  | 0.683 | | |  | | | 1.1 | |  | | 0.966 | |  | | | 0.881 | | | |  | |
| B4G0E0 | | Acyl-coenzyme A oxidase 4, peroxisomal | | K00232| [EC:1.3.3.6] | 1.5 | |  | | | 0.808 | |  | 0.53 | | |  | | | 1.2 | |  | | 0.971 | |  | | | 0.869 | | | |  | |
| C5Z483 | | Reticuline oxidase-like protein | | - | 1.361 | |  | | | 1.065 | |  | 0.77 | | |  | | | 0.8 | |  | | 0.304 | |  | | | 0.411 | | | |  | |
| C0PHW4 | | Putative uncharacterized protein | | - | 1.589 | |  | | | 1.202 | |  | 0.767 | | |  | | | 1.2 | |  | | 1 | |  | | | 0.898 | | | |  | |
| B6T5D7 | | Reticuline oxidase | | - | 6.676 | |  | | | 5.642 | |  | 1.414 | | |  | | | 0.5 | |  | | 0.979 | |  | | | 1.556 | | | |  | |
| **Hormones** | |  | |  |  | |  | | |  | |  |  | | |  | | |  | |  | |  | |  | | |  | | | |  | |
| Q9M588 | | Prohibitin | | - | 1.223 | |  | | | 0.887 | |  | 0.721 | | |  | | | 0.824 | |  | | 0.942 | |  | | | 1.113 | | | |  | |
| B4FQB3 | | IAA-amino acid hydrolase ILR1-like 1 | | K14664| [EC:3.5.1.-] | 1.392 | |  | | | 1.007 | |  | 0.726 | | |  | | | 0.828 | |  | | 0.757 | |  | | | 1.089 | | | |  | |
| B4FWD0 | | Minor allergen Alt a 7 | | K03809|Trp repressor binding protein | 1.278 | |  | | | 1.468 | |  | 1.098 | | |  | | | 0.713 | |  | | 0.818 | |  | | | 1.231 | | | |  | |
| C5WY36 | | Putative uncharacterized protein Sb01g005650 | | K11817|indole-3-acetaldehyde oxidase [EC:1.2.3.7]  K09842|abscisic-aldehyde oxidase [EC:1.2.3.14] | 1.47 | |  | | | 0.832 | |  | 0.486 | | |  | | | 1.196 | |  | | 1.101 | |  | | | 0.865 | | | |  | |
| C0PJP0 | | Cullin-associated NEDD8-dissociated protein 1 | | - | 2.467 | |  | | | 1.142 | |  | 0.557 | | |  | | | 0.845 | |  | | 1.019 | |  | | | 1.115 | | | |  | |
| C5YP89 | | Auxin-binding protein 5 (Fragment) | | - | 1.234 | |  | | | 1.052 | |  | 0.758 | | |  | | | 0.86 | |  | | 1.159 | |  | | | 1.455 | | | |  | |
| B4F9B5 | | Cysteine proteinase 2 | | K01366|cathepsin H [EC:3.4.22.16] | 1.662 | |  | | | 1.444 | |  | 0.959 | | |  | | | 1.172 | |  | | 1.377 | |  | | | 1.407 | | | |  | |
| B6TEB6 | | Thaumatin-like protein 1 | | - | 1.559 | |  | | | 1.78 | |  | 1.097 | | |  | | | 1.228 | |  | | 1.464 | |  | | | 1.45 | | | |  | |
| Q9FQB2 | | Probable glutathione S-transferase GSTU6 | | K00799| [EC:2.5.1.18] | 0.81 | |  | | | 0.693 | |  | 0.912 | | |  | | | 0.672 | |  | | 1.41 | |  | | | 2.097 | | | |  | |
| B4FQV3 | | Protein SGT1 homolog | | K12795|suppressor of G2 allele of SKP1 | 0.541 | |  | | | 0.765 | |  | 1.392 | | |  | | | 0.942 | |  | | 0.575 | |  | | | 0.621 | | | |  | |
| B4FIY7 | | NEDD8-conjugating enzyme Ubc12 | | K10579|ubiquitin-conjugating enzyme E2 M [EC:6.3.2.19] | 1.135 | |  | | | 1.27 | |  | 1.246 | | |  | | | 1.119 | |  | | 1.221 | |  | | | 1.235 | | | |  | |
| D0EP01 | | Protein SGT1 homolog | | K12795|suppressor of G2 allele of SKP1 | 0.517 | |  | | | 1.275 | |  | 2.428 | | |  | | | 0.861 | |  | | 0.679 | |  | | | 0.802 | | | |  | |
| B6TK27 | | Probable glutathione S-transferase GSTU6 | | K00799| [EC:2.5.1.18] | 2.873 | |  | | | 0.713 | |  | 0.228 | | |  | | | 1.393 | |  | | 1.454 | |  | | | 1.283 | | | |  | |
| C0P4Q4 | | Protochlorophyllide reductase B, chloroplastic OS | | K00218| [EC:1.3.1.33] | 1.298 | |  | | | 1.22 | |  | 0.98 | | |  | | | 0.825 | |  | | 0.736 | |  | | | 0.972 | | | |  | |
| B4FNZ5 | | 5'-methylthioadenosine/S-adenosylhomocysteine nucleosidase 1 | | K01244| [EC:3.2.2.16] | 1.446 | |  | | | 1.147 | |  | 0.703 | | |  | | | 0.875 | |  | | 0.795 | |  | | | 1.043 | | | |  | |
| B8A349 | | Serine/threonine-protein phosphatase 2A 65 kDa regulatory subunit A beta isoform | | K03456 | 1.976 | |  | | | 1.412 | |  | 0.694 | | |  | | | 1.162 | |  | | 0.969 | |  | | | 0.816 | | | |  | |
| B4F8H9 | | Probable 1-aminocyclopropane-1-carboxylate deaminase | | K01505| [EC:3.5.99.7] | 1.428 | |  | | | 1.041 | |  | 0.622 | | |  | | | 0.82 | |  | | 0.655 | |  | | | 0.876 | | | |  | |
| **Programmed cell death** | | | | |  | |  | | |  | |  |  | | |  | | |  | |  | |  | |  | | |  | | | |  | |
| B4G1Z4 | | Metacaspase-9 | | - | 0.821 | |  | | | 1.44 | |  | 1.471 | | |  | | | 1 | |  | | 1.033 | |  | | | 1.002 | | | |  | |
| B6TIC7 | | Cysteine proteinase 2 (Fragment) | | K15272|solute carrier family 35 (UDP-sugar transporter), member A1/2/3!K01376| [EC:3.4.22.-]  K01365|mtr:MTR_3g116080|cathepsin L [EC:3.4.22.15] | 0.855 | |  | | | 0.87 | |  | 1.002 | | |  | | | 0.9 | |  | | 0.846 | |  | | | 1.004 | | | |  | |
| C5XJZ6 | | Hexokinase-6 | | K00844| [EC:2.7.1.1] | 2.568 | |  | | | 1.03 | |  | 0.427 | | |  | | | 1 | |  | | 0.788 | |  | | | 0.903 | | | |  | |
| B6U4K6 | | Hexokinase-6 | | K00844| [EC:2.7.1.1] | 1.955 | |  | | | 1.03 | |  | 0.592 | | |  | | | 1.2 | |  | | 1.045 | |  | | | 0.956 | | | |  | |
| C0PIU9 | | Hexokinase-5 | | K00844| [EC:2.7.1.1] | 2.502 | |  | | | 1.59 | |  | 0.483 | | |  | | | 0.9 | |  | | 0.57 | |  | | | 0.651 | | | |  | |
| **Senescence/Aging** | | | |  |  | |  | | |  | |  |  | | |  | | |  | |  | |  | |  | | |  | | | |  | |
| B4F9B5 | | Cysteine proteinase 2 O | | K01366|cathepsin H [EC:3.4.22.16] | 1.662 | |  | | | 1.44 | |  | 0.959 | | |  | | | 1.2 | |  | | 1.377 | |  | | | 1.407 | | | |  | |
| B4FN58 | | Protein-L-isoaspartate O-methyltransferase | | K00573| [EC:2.1.1.77] | 1.307 | |  | | | 2.09 | |  | 1.702 | | |  | | | 1.1 | |  | | 1.715 | |  | | | 1.519 | | | |  | |
| B6SHX8 | | Copper transport protein ATOX1 | | K07213 | 0.434 | |  | | | 1.78 | |  | 3.793 | | |  | | | 0.9 | |  | | 0.692 | |  | | | 0.948 | | | |  | |
| C5Z0R1 | | Sulfite reductase [ferredoxin], chloroplastic | | K00392| [EC:1.8.7.1] | 1.521 | |  | | | 0.74 | |  | 0.477 | | |  | | | 1.1 | |  | | 0.752 | |  | | | 0.702 | | | |  | |
| **Morphogenesis** | | | | | | | | | | | | | | | | | | | | | | | | | | | | |  | | | | |
| B4FA02 | | Thylakoid lumenal 17.9 kDa protein, chloroplastic | | - | 0.676 | |  | | | 0.76 | |  | 1.113 | | |  | | | 1.5 | |  | | 0.805 | |  | | | 0.564 | | | |  | |
| B4FS63 | | Probable 26S proteasome non-ATPase regulatory subunit 7 | | K03038 | 1.777 | |  | | | 1.25 | |  | 0.69 | | |  | | | 0.8 | |  | | 1.429 | |  | | | 1.788 | | | |  | |
| B7ZY61 | | DNA damage-binding protein 1a | | K10610 | 2.77 | |  | | | 0.93 | |  | 0.322 | | |  | | | 1 | |  | | 1.493 | |  | | | 1.668 | | | |  | |
| C4JA45 | | 60S ribosomal protein L5-1 | | K02932 | 1.104 | |  | | | 1.43 | |  | 1.592 | | |  | | | 1.4 | |  | | 1.43 | |  | | | 1.059 | | | |  | |
| B6THG9 | | 60S ribosomal protein L5-1 | | K02932 | 1.098 | |  | | | 0.87 | |  | 0.944 | | |  | | | 1.4 | |  | | 1.634 | |  | | | 1.288 | | | |  | |
| B4G1X1 | | Alpha-galactosidase | | K07407 [EC:3.2.1.22] | 1.002 | |  | | | 0.75 | |  | 0.785 | | |  | | | 0.8 | |  | | 1.191 | |  | | | 1.392 | | | |  | |
| F2CS21 | | Calmodulin-1 | | K02183 | 0.396 | |  | | | 1.2 | |  | 2.892 | | |  | | | 1 | |  | | 1.305 | |  | | | 1.154 | | | |  | |
| **Growth** | |  | |  |  | |  | | |  | |  |  | | |  | | |  | |  | |  | |  | | |  | | | |  | |
| B4FL17 | | Translationally-controlled tumor protein homolog | | - | 1.584 | |  | | | 1.02 | |  | 0.643 | | |  | | | 1.2 | |  | | 1.451 | |  | | | 1.299 | | | |  | |
| B6TJC7 | | Membrane steroid-binding protein 2 | | - | 0.669 | |  | | | 1.23 | |  | 1.537 | | |  | | | 0.9 | |  | | 1.229 | |  | | | 1.583 | | | |  | |
| B6TPI3 | | Thioredoxin reductase NTRA | | K00384|thioredoxin reductase (NADPH) [EC:1.8.1.9] | 0.622 | |  | | | 0.76 | |  | 1.37 | | |  | | | 0.8 | |  | | 0.806 | |  | | | 0.625 | | | |  | |
| Q9M588 | | Prohibitin | | - | 1.223 | |  | | | 0.89 | |  | 0.721 | | |  | | | 0.8 | |  | | 0.942 | |  | | | 1.113 | | | |  | |
| B4FUT1 | | Peroxidase 15 | | K00430|[EC:1.11.1.7] | 0.824 | |  | | | 1.48 | |  | 1.806 | | |  | | | 0.8 | |  | | 1.107 | |  | | | 1.444 | | | |  | |
| G1FMQ3 | | Auxin-binding protein 1 | | - | 1.804 | |  | | | 2.02 | |  | 1.034 | | |  | | | 0.8 | |  | | 1.031 | |  | | | 1.313 | | | |  | |
| Q9FQB2 | | Probable glutathione S-transferase GSTU6 | | K00799|[EC:2.5.1.18] | 0.81 | |  | | | 0.69 | |  | 0.912 | | |  | | | 0.7 | |  | | 1.41 | |  | | | 2.097 | | | |  | |
| C0PES7 | | 3'(2'),5'-bisphosphate nucleotidase | | K15422|[EC:3.1.3.7 3.1.3.57] | 1.033 | |  | | | 1.02 | |  | 0.888 | | |  | | | 1.4 | |  | | 1.631 | |  | | | 1.184 | | | |  | |
| O04014 | | 40S ribosomal protein S6 | | K02991 | 0.958 | |  | | | 1.5 | |  | 1.715 | | |  | | | 1.2 | |  | | 1.09 | |  | | | 0.925 | | | |  | |
| B4FUC4 | | Salt stress root protein RS1 | | - | 0.306 | |  | | | 0.94 | |  | 3.095 | | |  | | | 1.1 | |  | | 1.994 | |  | | | 1.604 | | | |  | |
| B6TK27 | | Probable glutathione S-transferase GSTU6 | | K00799| [EC:2.5.1.18] | 2.873 | |  | | | 0.71 | |  | 0.228 | | |  | | | 1.4 | |  | | 1.454 | |  | | | 1.283 | | | |  | |
| B6TXN5 | | Probable carboxylesterase 18 | | K14493|gibberellin receptor GID1 [EC:3.-.-.-] | 1.641 | |  | | | 1.35 | |  | 0.812 | | |  | | | --- | |  | | --- | |  | | | 0.345 | | | |  | |
| A7XZC6 | | Phosphoethanolamine N-methyltransferase 2 | | K05929|phosphoethanolamine N-methyltransferase [EC:2.1.1.103] | 2.168 | |  | | | 0.92 | |  | 0.579 | | |  | | | 1 | |  | | 0.368 | |  | | | 0.388 | | | |  | |
| B4FRR4 | | Membrane steroid-binding protein 1 | | - | 1.403 | |  | | | 1.16 | |  | 0.919 | | |  | | | 1.2 | |  | | 1.594 | |  | | | 1.68 | | | |  | |
| B4FCD7 | | Probable glycerophosphoryl diester phosphodiesterase 1 | | K01126|[EC:3.1.4.46] | 2.29 | |  | | | 1.31 | |  | 0.567 | | |  | | | 1.1 | |  | | 1.265 | |  | | | 1.393 | | | |  | |
| Q9ZR86 | | Probable nucleoredoxin 1-1 | | - | 1.506 | |  | | | 0.92 | |  | 0.579 | | |  | | | 0.9 | |  | | 0.909 | |  | | | 1.002 | | | |  | |
| B4FKV6 | | Peroxidase 15 | | K00430|[EC:1.11.1.7] | 0.585 | |  | | | 1.58 | |  | 2.769 | | |  | | | 0.6 | |  | | 1.341 | |  | | | 1.982 | | | |  | |
| B6TKK2 | | Probable carboxylesterase 18 | | K14493|gibberellin receptor GID1 [EC:3.-.-.-] | 0.988 | |  | | | 0.92 | |  | 1.065 | | |  | | | 1 | |  | | 0.471 | |  | | | 0.504 | | | |  | |
| C0PGU8 | | Probable glycerophosphoryl diester phosphodiesterase 1 | | K01126|[EC:3.1.4.46] | 1.014 | |  | | | 0.86 | |  | 0.887 | | |  | | | 1.1 | |  | | 1.564 | |  | | | 1.483 | | | |  | |
| B4FBC9 | | Patellin-3 | | - | 1.178 | |  | | | 2.05 | |  | 1.318 | | |  | | | 1.2 | |  | | 0.943 | |  | | | 1.048 | | | |  | |
| B6U0V6 | | Endoplasmin homolog | | K09487|heat shock protein 90kDa beta | 1.402 | |  | | | 0.71 | |  | 0.579 | | |  | | | 0.8 | |  | | 0.452 | |  | | | 0.58 | | | |  | |
| C0P4T2 | | Patellin-5 | | - | 1.621 | |  | | | 1.63 | |  | 0.992 | | |  | | | 1 | |  | | 1.728 | |  | | | 1.762 | | | |  | |
| **Calcium** | |  | |  |  | |  | | |  | |  |  | | |  | | |  | |  | |  | |  | | |  | | | |  | |
| B4FUA8 | | Calreticulin-3 | | K08057 | 0.702 | |  | | | 0.416 | |  | 0.584 | | |  | | | 0.8 | |  | | 0.289 | |  | | | 0.385 | | | |  | |
| F2CS21 | | Calmodulin-1 | | K02183 | 0.396 | |  | | | 1.204 | |  | 2.892 | | |  | | | 1 | |  | | 1.305 | |  | | | 1.154 | | | |  | |
| C0P3S3 | | Calreticulin-3 | | K08057 | 1.455 | |  | | | 1.379 | |  | 0.932 | | |  | | | 1.2 | |  | | 0.414 | |  | | | 0.351 | | | |  | |
| C0PPF5 | | Calcium sensing receptor, chloroplastic OS | | - | 0.465 | |  | | | 0.776 | |  | 1.606 | | |  | | | 0.7 | |  | | 0.788 | |  | | | 1.06 | | | |  | |
| C3UZ63 | | Heat shock protein 81-1 | | K04079|molecular chaperone HtpG | 1.188 | |  | | | 1.039 | |  | 0.973 | | |  | | | 0.9 | |  | | 0.633 | |  | | | 0.835 | | | |  | |
| C5X3T6 | | Heat shock protein 81-3 | | K04079|molecular chaperone HtpG | 2.086 | |  | | | 1.188 | |  | 0.525 | | |  | | | 0.9 | |  | | 0.834 | |  | | | 1.043 | | | |  | |
| B4FUC4 | | Salt stress root protein RS1 | | - | 0.306 | |  | | | 0.941 | |  | 3.095 | | |  | | | 1.1 | |  | | 1.994 | |  | | | 1.604 | | | |  | |
| **Wounding** | |  | |  |  | |  | | |  | |  |  | | |  | | |  | |  | |  | |  | | |  | | | |  | |
| B6U016 | | Senescence-associated protein DIN1 | | - | 0.66 | |  | | | 0.673 | |  | 0.982 | | |  | | | 1 | |  | | 1.061 | |  | | | 0.978 | | | |  | |
| B4FCF6 | | Late embryogenesis abundant protein Lea14-A | | - | 2.801 | |  | | | 1.175 | |  | 0.43 | | |  | | | 0.6 | |  | | 1.709 | |  | | | 2.691 | | | |  | |
| B8A1V8 | | 4-coumarate--CoA ligase-like 4 | | K10526| [EC:6.2.1.-] | 2.065 | |  | | | 1.34 | |  | 0.929 | | |  | | | 1 | |  | | 1.032 | |  | | | 0.857 | | | |  | |
| B4G1T3 | | Bifunctional chitinase/lysozyme (Fragment) | | K01183| [EC:3.2.1.14] | 0.689 | |  | | | 0.533 | |  | 0.904 | | |  | | | 1.1 | |  | | 0.27 | |  | | | 0.186 | | | |  | |
| B6TVA3 | | Bifunctional chitinase/lysozyme (Fragment) | | K01183| [EC:3.2.1.14] | 0.937 | |  | | | 0.674 | |  | 0.967 | | |  | | | 0.7 | |  | | 0.483 | |  | | | 0.781 | | | |  | |
| B6U463 | | Subtilisin-chymotrypsin inhibitor-2B (Fragment) | | - | 1.945 | |  | | | 1.831 | |  | 0.85 | | |  | | | 1.5 | |  | | 1.11 | |  | | | 0.688 | | | |  | |
| Q42420 | | Subtilisin-chymotrypsin inhibitor WSCI | | - | 0.574 | |  | | | 1.697 | |  | 2.776 | | |  | | | 1.3 | |  | | 0.968 | |  | | | 0.852 | | | |  | |
| B6TMF0 | | Cystatin-1 | | - | 0.726 | |  | | | 1.236 | |  | 1.626 | | |  | | | 0.8 | |  | | 1.291 | |  | | | 1.689 | | | |  | |
| B6TT18 | | Allene oxide synthase 1, chloroplastic | | K01723|hydroperoxide dehydratase [EC:4.2.1.92] | 2.208 | |  | | | 0.789 | |  | 0.592 | | |  | | | 1.3 | |  | | 0.815 | |  | | | 0.666 | | | |  | |
| Q0GQW9 | | Allene oxide synthase 2 | | K01723|hydroperoxide dehydratase [EC:4.2.1.92] | 1.524 | |  | | | 1.32 | |  | 0.9 | | |  | | | 0.9 | |  | | 0.854 | |  | | | 1.054 | | | |  | |
| B7ZY42 | | Allene oxide synthase 2 | | K01723|hydroperoxide dehydratase [EC:4.2.1.92] | 3.465 | |  | | | 2.057 | |  | 0.59 | | |  | | | 1 | |  | | 1.756 | |  | | | 1.872 | | | |  | |
| B4FQB3 | | IAA-amino acid hydrolase ILR1-like 1 | | K14664|IAA-amino acid hydrolase [EC:3.5.1.-] | 1.392 | |  | | | 1.007 | |  | 0.726 | | |  | | | 0.8 | |  | | 0.757 | |  | | | 1.089 | | | |  | |
| Q49HD7 | | 12-oxophytodienoate reductase 7 | | K05894| [EC:1.3.1.42] | 1.455 | |  | | | 1.111 | |  | 0.752 | | |  | | | 1.1 | |  | | 1.267 | |  | | | 1.2 | | | |  | |
| C0P848 | | Formate dehydrogenase 1, mitochondrial | | K00122| [EC:1.2.1.2] | 1.859 | |  | | | 1.023 | |  | 0.515 | | |  | | | 1 | |  | | 1.296 | |  | | | 1.232 | | | |  | |
| Q9ZTQ4 | | Beta-fructofuranosidase, insoluble isoenzyme 7 | | K01193| [EC:3.2.1.26] | 0.49 | |  | | | 0.989 | |  | 1.725 | | |  | | | 1.3 | |  | | 1.207 | |  | | | 0.946 | | | |  | |
| B7ZX90 | | Beta-fructofuranosidase, insoluble isoenzyme 4 | | K01193| [EC:3.2.1.26] | 0.709 | |  | | | 0.946 | |  | 1.331 | | |  | | | 1.1 | |  | | 1.171 | |  | | | 1.083 | | | |  | |
| B7ZZM5 | | Beta-fructofuranosidase, cell wall isozyme | | K01193| [EC:3.2.1.26] | 0.921 | |  | | | 0.466 | |  | 0.557 | | |  | | | 1.3 | |  | | 0.605 | |  | | | 0.557 | | | |  | |
| **Salt/Water/Desiccation** | | | |  |  | |  | | |  | |  |  | | |  | | |  | |  | |  | |  | | |  | | | |  | |
| Q1KSB0 | | Putative aconitate hydratase, cytoplasmic | | K01681|[EC:4.2.1.3] | 1.5 | |  | | | 1.34 | |  | 0.784 | | |  | | | 0.8 | |  | | 1.027 | |  | | | 1.248 | | | |  | |
| B4G0L9 | | Putative uncharacterized protein | | K00134|glyceraldehyde 3-phosphate dehydrogenase [EC:1.2.1.12] | 2.079 | |  | | | 2.08 | |  | 0.85 | | |  | | | 0.7 | |  | | 0.969 | |  | | | 1.111 | | | |  | |
| B4FK49 | | Nucleoside diphosphate kinase 1 | | K00940|[EC:2.7.4.6] | 1.302 | |  | | | 0.9 | |  | 0.775 | | |  | | | 0.8 | |  | | 1.064 | |  | | | 1.351 | | | |  | |
| Q9M588 | | Prohibitin | | - | 1.223 | |  | | | 0.89 | |  | 0.721 | | |  | | | 0.8 | |  | | 0.942 | |  | | | 1.113 | | | |  | |
| Q43863 | | Annexin D7 | | - | 1.354 | |  | | | 1.4 | |  | 0.83 | | |  | | | 0.9 | |  | | 1.361 | |  | | | 1.175 | | | |  | |
| C4J9M7 | | 2-Cys peroxiredoxin BAS1, chloroplastic | | K03386|[EC:1.11.1.15] | 0.831 | |  | | | 0.64 | |  | 0.738 | | |  | | | 1 | |  | | 1.766 | |  | | | 1.74 | | | |  | |
| B4FNW1 | | Triosephosphate isomerase, cytosolic | | K01803|[EC:5.3.1.1] | 1.937 | |  | | | 1.45 | |  | 0.762 | | |  | | | 0.9 | |  | | 1.636 | |  | | | 1.692 | | | |  | |
| Q8W0Q2 | | Delta-1-pyrroline-5-carboxylate dehydrogenase 12A1, mitochondrial | | K00294|[EC:1.5.1.12] | 3.129 | |  | | | 0.98 | |  | 0.298 | | |  | | | 1.5 | |  | | 1.897 | |  | | | 1.162 | | | |  | |
| B4FUI0 | | NADH dehydrogenase [ubiquinone] 1 alpha subcomplex subunit 9, mitochondrial | | K03953|1|0.0|837|zma:100282690|NADH dehydrogenase (ubiquinone) 1 alpha subcomplex 9 [EC:1.6.5.3 1.6.99.3] | 2.073 | |  | | | 1.17 | |  | 0.599 | | |  | | | 0.9 | |  | | 1.144 | |  | | | 1.24 | | | |  | |
| I1I4L3 | | Cyanate hydratase | | K01725|[EC:4.2.1.104] | 0.763 | |  | | | 0.79 | |  | 0.951 | | |  | | | 0.7 | |  | | 1.127 | |  | | | 1.429 | | | |  | |
| C0PHD8 | | Aldehyde dehydrogenase family 7 member A1 | | K14085|[EC:1.2.1.31 1.2.1.8 1.2.1.3] | 2.796 | |  | | | 1.19 | |  | 0.371 | | |  | | | 1 | |  | | 1.86 | |  | | | 1.991 | | | |  | |
| B4FLQ8 | | 26S proteasome non-ATPase regulatory subunit 14 | | K03030 | 1.12 | |  | | | 1.14 | |  | 1.131 | | |  | | | 0.9 | |  | | 1.143 | |  | | | 1.279 | | | |  | |
| B4FL49 | | Transcription factor BTF3 homolog 4 | | K01527|nascent polypeptide-associated complex subunit beta | 0.76 | |  | | | 1.42 | |  | 2.053 | | |  | | | 1.5 | |  | | 0.985 | |  | | | 0.728 | | | |  | |
| B4FS99 | | ATP synthase subunit d, mitochondrial | | K02138|F-type H+-transporting ATPase subunit d [EC:3.6.3.14] | 0.759 | |  | | | 1.13 | |  | 1.449 | | |  | | | 1.1 | |  | | 1.79 | |  | | | 1.449 | | | |  | |
| B4FUT1 | | Peroxidase 15 | | K00430|[EC:1.11.1.7] | 0.824 | |  | | | 1.48 | |  | 1.806 | | |  | | | 0.8 | |  | | 1.107 | |  | | | 1.444 | | | |  | |
| B6T391 | | Lichenase-2 (Fragment) | | - | 0.45 | |  | | | 0.84 | |  | 1.829 | | |  | | | 0.7 | |  | | 0.833 | |  | | | 1.152 | | | |  | |
| B4G194 | | 40S ribosomal protein SA | | K02998 | 1.692 | |  | | | 1.22 | |  | 0.687 | | |  | | | 1.1 | |  | | 1.017 | |  | | | 1.037 | | | |  | |
| Q9XGW8 | | V-type proton ATPase subunit E | | K02150|[EC:3.6.3.14] | 0.966 | |  | | | 0.83 | |  | 0.834 | | |  | | | 0.9 | |  | | 1.044 | |  | | | 1.194 | | | |  | |
| B4G177 | | Transketolase, chloroplastic | | K00615|[EC:2.2.1.1] | 1.021 | |  | | | 1.17 | |  | 1.098 | | |  | | | 0.9 | |  | | 1.032 | |  | | | 1.219 | | | |  | |
| B4FNM4 | | 60S acidic ribosomal protein P0 | | K02941 | 1.17 | |  | | | 1.43 | |  | 1.173 | | |  | | | 1.2 | |  | | 2.625 | |  | | | 2.538 | | | |  | |
| B4FTK9 | | Endo-1,3;1,4-beta-D-glucanase | | K01061|carboxymethylenebutenolidase [EC:3.1.1.45] | 1.026 | |  | | | 0.58 | |  | 0.652 | | |  | | | 1.1 | |  | | 0.476 | |  | | | 0.363 | | | |  | |
| C4J094 | | Superoxide dismutase [Cu-Zn] 2 | | K04565|[EC:1.15.1.1] | 0.809 | |  | | | 1.73 | |  | 2.006 | | |  | | | 1.4 | |  | | 1.409 | |  | | | 1.21 | | | |  | |
| C0PDA6 | | Fumarate hydratase 1, mitochondrial | | K01679|[EC:4.2.1.2] | 1.335 | |  | | | 1.07 | |  | 0.78 | | |  | | | 0.8 | |  | | 1.225 | |  | | | 1.359 | | | |  | |
| B4FS18 | | L-ascorbate peroxidase 1, cytosolic | | K00434|[EC:1.11.1.11] | 1.354 | |  | | | 3.06 | |  | 2.304 | | |  | | | 0.8 | |  | | 0.933 | |  | | | 1.227 | | | |  | |
| B4FAL9 | | Fructose-bisphosphate aldolase, cytoplasmic isozyme | | K01623|[EC:4.1.2.13] | 1.746 | |  | | | 1.72 | |  | 1.011 | | |  | | | 0.8 | |  | | 1.047 | |  | | | 1.26 | | | |  | |
| B4FSZ7 | | Probable mitochondrial-processing peptidase subunit alpha-2 | | K01412|[EC:3.4.24.64] | 1.336 | |  | | | 0.97 | |  | 0.708 | | |  | | | 0.8 | |  | | 1.086 | |  | | | 1.46 | | | |  | |
| C0P758 | | Malic enzyme | | K00028|malate dehydrogenase (decarboxylating) [EC:1.1.1.39] | 1.933 | |  | | | 1.11 | |  | 0.565 | | |  | | | 1.1 | |  | | 0.899 | |  | | | 0.802 | | | |  | |
| C0PFV1 | | Plasma membrane ATPase 1 | | K01535|H+-transporting ATPase [EC:3.6.3.6] | 1.156 | |  | | | 1.03 | |  | 0.777 | | |  | | | 1.2 | |  | | 0.572 | |  | | | 0.528 | | | |  | |
| B4FZU8 | | Malate dehydrogenase, mitochondrial | | K00026|[EC:1.1.1.37] | 0.804 | |  | | | 1.46 | |  | 1.824 | | |  | | | 0.9 | |  | | 0.798 | |  | | | 1.058 | | | |  | |
| B4FRI7 | | Peptidyl-prolyl cis-trans isomerase CYP20-3, chloroplastic | | K03768|peptidyl-prolyl cis-trans isomerase B (cyclophilin B) [EC:5.2.1.8] | 0.645 | |  | | | 1.3 | |  | 1.945 | | |  | | | 0.8 | |  | | 0.794 | |  | | | 0.901 | | | |  | |
| C3UZ63 | | Heat shock protein 81-1 | | K04079|molecular chaperone HtpG | 1.188 | |  | | | 1.04 | |  | 0.973 | | |  | | | 0.9 | |  | | 0.633 | |  | | | 0.835 | | | |  | |
| B4FUA7 | | Aldehyde dehydrogenase family 3 member I1, chloroplastic | | K00128|[EC:1.2.1.3] | 1.405 | |  | | | 0.93 | |  | 0.631 | | |  | | | 0.9 | |  | | 0.883 | |  | | | 1.057 | | | |  | |
| B6SK54 | | Superoxide dismutase [Cu-Zn] 4AP | | K04565|[EC:1.15.1.1] | 1.075 | |  | | | 1.97 | |  | 1.602 | | |  | | | 1.3 | |  | | 2.171 | |  | | | 1.747 | | | |  | |
| B4FWP0 | | Fructose-bisphosphate aldolase, cytoplasmic isozyme | | K01623|[EC:4.1.2.13] | 2.043 | |  | | | 1.56 | |  | 0.677 | | |  | | | 0.9 | |  | | 1.237 | |  | | | 1.496 | | | |  | |
| B4FUE7 | | S-adenosylmethionine synthase 1 | | K00789|[EC:2.5.1.6] | 1.229 | |  | | | 1.53 | |  | 1.202 | | |  | | | 1.1 | |  | | 0.402 | |  | | | 0.378 | | | |  | |
| B6TNS8 | | Lichenase-2 (Fragment) | | - | 0.967 | |  | | | 1.27 | |  | 1.255 | | |  | | | 0.8 | |  | | 0.579 | |  | | | 0.776 | | | |  | |
| B6TM40 | | 40S ribosomal protein S3-3 | | K02985 | 1.988 | |  | | | 1.32 | |  | 0.685 | | |  | | | 1.1 | |  | | 1.055 | |  | | | 0.999 | | | |  | |
| B6TX10 | | 6-phosphogluconate dehydrogenase, decarboxylating | | K00033|[EC:1.1.1.44] | 1.282 | |  | | | 0.96 | |  | 0.636 | | |  | | | 1.1 | |  | | 0.477 | |  | | | 0.526 | | | |  | |
| Q2XXK4 | | Zeamatin | | - | 1.842 | |  | | | 0.94 | |  | 0.54 | | |  | | | 1.1 | |  | | 6.013 | |  | | | 5.37 | | | |  | |
| C0PB60 | | Gamma carbonic anhydrase 2, mitochondrial | | - | 1.06 | |  | | | 1.35 | |  | 1.32 | | |  | | | 0.7 | |  | | 1.033 | |  | | | 1.538 | | | |  | |
| B4FX40 | | Cysteine proteinase 1 | | K01376|cathepsin F [EC:3.4.22.41] | 0.588 | |  | | | 1.14 | |  | 1.414 | | |  | | | 1.2 | |  | | 0.57 | |  | | | 0.553 | | | |  | |
| C5Z0R1 | | Sulfite reductase [ferredoxin], chloroplastic | | K00392|[EC:1.8.7.1] | 1.521 | |  | | | 0.74 | |  | 0.477 | | |  | | | 1.1 | |  | | 0.752 | |  | | | 0.702 | | | |  | |
| Q6RW09 | | Allene oxide cyclase 4, chloroplastic | | K10525|[EC:5.3.99.6] | 2.037 | |  | | | 1.72 | |  | 0.844 | | |  | | | 1.1 | |  | | 0.938 | |  | | | 0.857 | | | |  | |
| B6TVA3 | | Bifunctional chitinase/lysozyme (Fragment) | | K01183|chitinase [EC:3.2.1.14] | 0.937 | |  | | | 0.67 | |  | 0.967 | | |  | | | 0.7 | |  | | 0.483 | |  | | | 0.781 | | | |  | |
| B4FHH4 | | Triosephosphate isomerase, cytosolic | | K01803|[EC:5.3.1.1] | 1.103 | |  | | | 1.01 | |  | 0.894 | | |  | | | 1 | |  | | 1.933 | |  | | | 1.992 | | | |  | |
| C0PHC0 | | V-type proton ATPase catalytic subunit A (Fragment) | | K02145|[EC:3.6.3.14] | 1.32 | |  | | | 1.08 | |  | 0.806 | | |  | | | 1.1 | |  | | 1.666 | |  | | | 1.48 | | | |  | |
| B6TMG9 | | Gamma carbonic anhydrase-like 2, mitochondrial | | - | 1.104 | |  | | | 1.08 | |  | 1.092 | | |  | | | 0.8 | |  | | 1.083 | |  | | | 1.227 | | | |  | |
| B4FAD9 | | UTP--glucose-1-phosphate uridylyltransferase | | K00963|[EC:2.7.7.9] | 1.663 | |  | | | 0.92 | |  | 0.575 | | |  | | | 1 | |  | | 1.466 | |  | | | 1.58 | | | |  | |
| B4FPA0 | | Endo-1,3;1,4-beta-D-glucanase | | K01061|carboxymethylenebutenolidase [EC:3.1.1.45] | 1.484 | |  | | | 0.78 | |  | 0.549 | | |  | | | 0.9 | |  | | 0.94 | |  | | | 0.96 | | | |  | |
| C0HER4 | | Putative aconitate hydratase, cytoplasmic | | K01681|[EC:4.2.1.3] | 1.116 | |  | | | 0.55 | |  | 0.4 | | |  | | | 0.8 | |  | | 0.962 | |  | | | 1.172 | | | |  | |
| B4FML9 | |  | | K00161|[EC:1.2.4.1] | 1.951 | |  | | | 0.83 | |  | 0.418 | | |  | | | 1 | |  | | 1.858 | |  | | | 1.962 | | | |  | |
| C5X3T6 | | Heat shock protein 81-3 | | K04079|1|0.0|1178|sbi:SORBI_07g028270|molecular chaperone HtpG | 2.086 | |  | | | 1.19 | |  | 0.525 | | |  | | | 0.9 | |  | | 0.834 | |  | | | 1.043 | | | |  | |
| Q9ZQY2 | | Pyruvate dehydrogenase E1 beta subunit isoform 2 | | K00162|[EC:1.2.4.1] | 1.241 | |  | | | 1.1 | |  | 0.802 | | |  | | | 0.8 | |  | | 1.208 | |  | | | 1.589 | | | |  | |
| B4FRR1 | | Soluble inorganic pyrophosphatase 1, chloroplastic | | K01507|[EC:3.6.1.1] | 0.208 | |  | | | 1.1 | |  | 5.285 | | |  | | | 0.8 | |  | | 1.591 | |  | | | 1.956 | | | |  | |
| B4FUC4 | | Salt stress root protein RS1 | | - | 0.306 | |  | | | 0.94 | |  | 3.095 | | |  | | | 1.1 | |  | | 1.994 | |  | | | 1.604 | | | |  | |
| B4FHT1 | | Annexin D7 | | - | 1.529 | |  | | | 1.16 | |  | 0.744 | | |  | | | 1.2 | |  | | 2.162 | |  | | | 2.035 | | | |  | |
| B6SHX8 | | Copper transport protein ATOX1 | | K07213 | 0.434 | |  | | | 1.78 | |  | 3.793 | | |  | | | 0.9 | |  | | 0.692 | |  | | | 0.948 | | | |  | |
| C0P7U9 | | DEAD-box ATP-dependent RNA helicase 9 | | K12823|[EC:3.6.4.13] | 0.583 | |  | | | 1.51 | |  | 2.554 | | |  | | | 1.5 | |  | | 1.06 | |  | | | 0.704 | | | |  | |
| B4FRR1 | | Soluble inorganic pyrophosphatase 1, chloroplastic | | K01507|inorganic pyrophosphatase [EC:3.6.1.1] | 0.338 | |  | | | 0.83 | |  | 2.725 | | |  | | | 1 | |  | | 1.441 | |  | | | 1.418 | | | |  | |
| B4FR89 | | Phosphomannomutase | | K01840|[EC:5.4.2.8] | 1.879 | |  | | | 1.05 | |  | 0.515 | | |  | | | 1 | |  | | 0.886 | |  | | | 0.887 | | | |  | |
| B4FT28 | | Glutamine synthetase, chloroplastic | | K01915|[EC:6.3.1.2] | 0.481 | |  | | | 0.73 | |  | 1.451 | | |  | | | 1.1 | |  | | 0.775 | |  | | | 0.757 | | | |  | |
| C0P5Y3 | | 5-methyltetrahydropteroyltriglutamate--homocysteine methyltransferase | | K00549|[EC:2.1.1.14] | 1.787 | |  | | | 1.09 | |  | 0.576 | | |  | | | 1.2 | |  | | 0.955 | |  | | | 0.713 | | | |  | |
| C0PHC0 | | V-type proton ATPase catalytic subunit A (Fragment) | | K02145|V-type H+-transporting ATPase subunit A [EC:3.6.3.14] | 1.909 | |  | | | 1.02 | |  | 0.578 | | |  | | | 0.9 | |  | | 1.245 | |  | | | 1.35 | | | |  | |
| C0PD27 | | Isocitrate dehydrogenase [NADP] | | K00031|isocitrate dehydrogenase [EC:1.1.1.42] | 2.354 | |  | | | 1.45 | |  | 0.545 | | |  | | | 1.2 | |  | | 1.103 | |  | | | 0.94 | | | |  | |
| C0HHC4 | | Nucleoside diphosphate kinase 1 | | K00940|[EC:2.7.4.6] | 0.802 | |  | | | 0.74 | |  | 0.978 | | |  | | | 0.7 | |  | | 1.137 | |  | | | 1.83 | | | |  | |
| B4FVD5 | | V-type proton ATPase subunit E | | K02150|V-type H+-transporting ATPase subunit E [EC:3.6.3.14] | 1.08 | |  | | | 1.01 | |  | 0.911 | | |  | | | 0.9 | |  | | 0.709 | |  | | | 0.713 | | | |  | |
| B4F9H6 | | Superoxide dismutase [Mn] 3.4, mitochondrial | | K04564|superoxide dismutase, Fe-Mn family [EC:1.15.1.1] | 1.445 | |  | | | 0.87 | |  | 0.583 | | |  | | | 1 | |  | | 2.062 | |  | | | 2.083 | | | |  | |
| B4G1T3 | | Bifunctional chitinase/lysozyme (Fragment) | | K01183|[EC:3.2.1.14] | 0.689 | |  | | | 0.53 | |  | 0.904 | | |  | | | 1.1 | |  | | 0.27 | |  | | | 0.186 | | | |  | |
| Q2XXA8 | | Glucan endo-1,3-beta-glucosidase, acidic isoform | | - | 0.835 | |  | | | 0.67 | |  | 1.14 | | |  | | | 1 | |  | | 0.134 | |  | | | 0.289 | | | |  | |
| B6TF86 | | Hydroxyacylglutathione hydrolase 3, mitochondrial | | K01069|[EC:3.1.2.6] | 0.958 | |  | | | 1.08 | |  | 1.262 | | |  | | | 0.5 | |  | | 0.753 | |  | | | 1.264 | | | |  | |
| C5XN52 | | Pathogenesis-related protein (Fragment) | | - | 2.176 | |  | | | 2.14 | |  | 0.93 | | |  | | | 1.7 | |  | | 3.068 | |  | | | 2.224 | | | |  | |
| B4FRJ3 | | Proteasome subunit beta type-6 | | K02738|20S proteasome subunit beta 1 [EC:3.4.25.1] | 1.266 | |  | | | 1.16 | |  | 0.905 | | |  | | | 0.8 | |  | | 1.2 | |  | | | 1.381 | | | |  | |
| B4FN58 | | Protein-L-isoaspartate O-methyltransferase | | K00573|[EC:2.1.1.77] | 1.307 | |  | | | 2.09 | |  | 1.702 | | |  | | | 1.1 | |  | | 1.715 | |  | | | 1.519 | | | |  | |
| C0HI97 | | ATP synthase subunit delta', mitochondrial | | K02134|F-type H+-transporting ATPase subunit delta [EC:3.6.3.14] | 1.613 | |  | | | 1.37 | |  | 0.806 | | |  | | | 1.1 | |  | | 0.99 | |  | | | 1.04 | | | |  | |
| E1AFV5 | | Glucan endo-1,3-beta-glucosidase GII | | - | 3.144 | |  | | | 2.28 | |  | 0.666 | | |  | | | 0.6 | |  | | 1.674 | |  | | | 2.56 | | | |  | |
| B4FKV6 | | Peroxidase 15 | | K00430|[EC:1.11.1.7] | 0.585 | |  | | | 1.58 | |  | 2.769 | | |  | | | 0.6 | |  | | 1.341 | |  | | | 1.982 | | | |  | |
| B4FHX7 | | Endo-1,3;1,4-beta-D-glucanase | | K01061|carboxymethylenebutenolidase [EC:3.1.1.45] | 3.156 | |  | | | 0.8 | |  | 0.268 | | |  | | | 1.4 | |  | | 4.39 | |  | | | 3.045 | | | |  | |
| B4FUM4 | | Aquaporin PIP1-3/PIP1-4 | | K09872 | 2.336 | |  | | | 1.41 | |  | 0.749 | | |  | | | 1 | |  | | 1.015 | |  | | | 0.974 | | | |  | |
| C0HEE9 | | Transcription factor BTF3 homolog 4 | | K01527|nascent polypeptide-associated complex subunit beta | 0.628 | |  | | | 1.62 | |  | 2.061 | | |  | | | 1.1 | |  | | 0.742 | |  | | | 0.679 | | | |  | |
| B6TNN9 | | Kynurenine formamidase | | - | 1.485 | |  | | | 1.25 | |  | 0.79 | | |  | | | 1 | |  | | 1.261 | |  | | | 1.585 | | | |  | |
| B4F8T3 | | Spartin | | - | 1.414 | |  | | | 0.97 | |  | 0.758 | | |  | | | 1.1 | |  | | 0.798 | |  | | | 0.826 | | | |  | |
| Q43264 | | Alcohol dehydrogenase 1 | | K00001|[EC:1.1.1.1] | 1.343 | |  | | | 2.05 | |  | 1.239 | | |  | | | 1.2 | |  | | 1.346 | |  | | | 1.545 | | | |  | |
| C4J0S0 | | Probable mannose-1-phosphate guanylyltransferase 3 | | K00966|[EC:2.7.7.13] | 1.295 | |  | | | 1.44 | |  | 1.243 | | |  | | | 0.8 | |  | | 0.637 | |  | | | 0.672 | | | |  | |
| B8A3G8 | | Glycine-rich RNA-binding protein 1 (Fragment) | | K13195|cold-inducible RNA-binding protein!  K02965|2small subunit ribosomal protein S19!  K11294|nucleolin!  K12741|heterogeneous nuclear ribonucleoprotein A1/A3 | 0.623 | |  | | | 0.87 | |  | 1.341 | | |  | | | 1.2 | |  | | 0.915 | |  | | | 0.777 | | | |  | |
| C4JBB8 | | Heat shock 70 kDa protein, mitochondrial | | K04043|molecular chaperone DnaK | 1.303 | |  | | | 1.14 | |  | 0.873 | | |  | | | 0.8 | |  | | 0.902 | |  | | | 1.068 | | | |  | |
| B6TCK3 | | NADH-cytochrome b5 reductase-like protein | | K00326|[EC:1.6.2.2] | 0.991 | |  | | | 1.28 | |  | 1.207 | | |  | | | 1.4 | |  | | 1.99 | |  | | | 1.447 | | | |  | |
| C6JSH7 | | Germin-like protein 8-7 | | - | 0.685 | |  | | | 0.6 | |  | 0.866 | | |  | | | 0.6 | |  | | 3.722 | |  | | | 6.201 | | | |  | |
| Q4TZJ2 | | Pyrroline-5-carboxylate reductase | | K00286|[EC:1.5.1.2] | 1.769 | |  | | | 0.9 | |  | 0.521 | | |  | | | 0.9 | |  | | 2.076 | |  | | | 2.243 | | | |  | |
| B4F9J5 | | Glutamate dehydrogenase | | K00261|[EC:1.4.1.3] | 1.431 | |  | | | 0.66 | |  | 0.38 | | |  | | | 0.9 | |  | | 0.957 | |  | | | 0.91 | | | |  | |
| B6SUD1 | | Alcohol dehydrogenase 2 | | K00001|[EC:1.1.1.1] | 1.419 | |  | | | 0.7 | |  | 0.615 | | |  | | | 1 | |  | | 0.918 | |  | | | 0.947 | | | |  | |
| D4P940 | | Transcription factor BTF3 homolog 4 | | K01527|nascent polypeptide-associated complex subunit beta | 0.558 | |  | | | 1.37 | |  | 2.507 | | |  | | | 1.7 | |  | | 1.169 | |  | | | 0.611 | | | |  | |
| B6U0V6 | | Endoplasmin homolog | | K09487|heat shock protein 90kDa beta | 1.402 | |  | | | 0.71 | |  | 0.579 | | |  | | | 0.8 | |  | | 0.452 | |  | | | 0.58 | | | |  | |
| B6TM55 | | L-ascorbate peroxidase 1, cytosolic | | K00434|[EC:1.11.1.11] | 0.862 | |  | | | 0.69 | |  | 0.731 | | |  | | | 0.9 | |  | | 0.944 | |  | | | 1.123 | | | |  | |
| B6THU9 | | Peroxidase 30 | | K00430|[EC:1.11.1.7] | 1.421 | |  | | | 2.07 | |  | 1.484 | | |  | | | 0.7 | |  | | 1.287 | |  | | | 2.012 | | | |  | |
| C0PHD8 | | Aldehyde dehydrogenase family 7 member A1 | | K14085|[EC:1.2.1.31 1.2.1.8 1.2.1.3] | 2.796 | |  | | | 1.19 | |  | 0.371 | | |  | | | 1 | |  | | 1.86 | |  | | | 1.991 | | | |  | |
| B4FA22 | | Aquaporin PIP2-1 | | K09872 | 1.894 | |  | | | 1.52 | |  | 0.776 | | |  | | | 0.9 | |  | | 0.719 | |  | | | 0.751 | | | |  | |
| B4FCF6 | | Late embryogenesis abundant protein Lea14-A | | - | 2.801 | |  | | | 1.18 | |  | 0.43 | | |  | | | 0.6 | |  | | 1.709 | |  | | | 2.691 | | | |  | |
| B6SKV1 | | Delta-1-pyrroline-5-carboxylate synthase | | K12657|[EC:2.7.2.11 1.2.1.41] | 1.874 | |  | | | 1.43 | |  | 0.663 | | |  | | | 1 | |  | | 2.508 | |  | | | 1.778 | | | |  | |
| B4F9K0 | | Late embryogenesis abundant protein Lea14-A | | - | 1.728 | |  | | | 1.43 | |  | 0.898 | | |  | | | 0.8 | |  | | 1.743 | |  | | | 2.076 | | | |  | |
| B4FUA7 | | Aldehyde dehydrogenase family 3 member I1, chloroplastic | | K00128| [EC:1.2.1.3] | 1.405 | |  | | | 0.93 | |  | 0.631 | | |  | | | 0.9 | |  | | 0.883 | |  | | | 1.057 | | | |  | |
| B4FX40 | | Cysteine proteinase 1 | | K01376|[EC:3.4.22.41] | 0.588 | |  | | | 1.14 | |  | 1.414 | | |  | | | 1.2 | |  | | 0.57 | |  | | | 0.553 | | | |  | |
| Q6RW09 | | Allene oxide cyclase 4, chloroplastic | | K10525|[EC:5.3.99.6] | 2.037 | |  | | | 1.72 | |  | 0.844 | | |  | | | 1.1 | |  | | 0.938 | |  | | | 0.857 | | | |  | |
| **Cold** | |  | |  |  | |  | | |  | |  |  | | |  | | |  | |  | |  | |  | | |  | | | |  | |
| B6THU9 | | Peroxidase 30 | | K00430|[EC:1.11.1.7] | 1.421 | |  | | | 2.07 | |  | 1.484 | | |  | | | 0.7 | |  | | 1.287 | |  | | | 2.012 | | | |  | |
| B4FJM9 | | Fructose-1,6-bisphosphatase, chloroplastic | | K03841|[EC:3.1.3.11] | 0.981 | |  | | | 0.62 | |  | 0.574 | | |  | | | 1 | |  | | 0.767 | |  | | | 0.846 | | | |  | |
| C0P530 | | RuBisCO large subunit-binding protein subunit beta, chloroplastic (Fragment) | | K04077|chaperonin GroEL | 0.956 | |  | | | 0.96 | |  | 0.999 | | |  | | | 1 | |  | | 1.418 | |  | | | 1.576 | | | |  | |
| B6T4U8 | | Probable calcium-binding protein CML7 | | K13448|calcium-binding protein CML | 0.705 | |  | | | 1.35 | |  | 1.998 | | |  | | | 1.1 | |  | | 0.76 | |  | | | 1.236 | | | |  | |
| C0P5X6 | | Ribulose bisphosphate carboxylase/oxygenase activase, chloroplastic | | K03064|26S proteasome regulatory subunit T4 | 0.734 | |  | | | 0.9 | |  | 1.047 | | |  | | | 0.3 | |  | | 0.854 | |  | | | 3.115 | | | |  | |
| B4FM07 | | 2-Cys peroxiredoxin BAS1, chloroplastic | | K03386|[EC:1.11.1.15] | 0.801 | |  | | | 0.6 | |  | 0.756 | | |  | | | 1 | |  | | 1.229 | |  | | | 1.22 | | | |  | |
| Q43863 | | Annexin D7 | | - | 1.354 | |  | | | 1.4 | |  | 0.83 | | |  | | | 0.9 | |  | | 1.361 | |  | | | 1.175 | | | |  | |
| C4J9M7 | | 2-Cys peroxiredoxin BAS1, chloroplastic | | K03386|[EC:1.11.1.15] | 0.831 | |  | | | 0.64 | |  | 0.738 | | |  | | | 1 | |  | | 1.766 | |  | | | 1.74 | | | |  | |
| B4FVN0 | | Probable galactinol--sucrose galactosyltransferase 6 | | K06617|raffinose synthase [EC:2.4.1.82] | 2.288 | |  | | | 0.87 | |  | 0.374 | | |  | | | 1 | |  | | 0.582 | |  | | | 0.617 | | | |  | |
| C0HHU2 | | 2,3-bisphosphoglycerate-independent phosphoglycerate mutase | | K15633|[EC:5.4.2.1] | 1.654 | |  | | | 1.17 | |  | 0.694 | | |  | | | 0.8 | |  | | 0.865 | |  | | | 1.111 | | | |  | |
| C0PDB0 | | Phosphoglycerate kinase, chloroplastic | | K00927|[EC:2.7.2.3] | 0.789 | |  | | | 0.67 | |  | 0.907 | | |  | | | 0.9 | |  | | 0.803 | |  | | | 0.917 | | | |  | |
| C0PC75 | | Thioredoxin M2, chloroplastic | | K03671 | 0.365 | |  | | | 0.68 | |  | 2.056 | | |  | | | 1.2 | |  | | 1.164 | |  | | | 1.079 | | | |  | |
| B4F8L7 | | Glyceraldehyde-3-phosphate dehydrogenase B, chloroplastic (Fragment) | | K05298|[EC:1.2.1.13] | 0.796 | |  | | | 0.76 | |  | 0.938 | | |  | | | 1.1 | |  | | 0.696 | |  | | | 0.635 | | | |  | |
| B6U0S1 | | Elongation factor 2 | | K03234 | 1.603 | |  | | | 1.29 | |  | 0.784 | | |  | | | 0.9 | |  | | 0.821 | |  | | | 0.989 | | | |  | |
| B6TWC3 | | Germin-like protein 5-1 | | - | 1.038 | |  | | | 1.73 | |  | 1.862 | | |  | | | 0.6 | |  | | 0.822 | |  | | | 1.52 | | | |  | |
| B4FQ59 | | Phosphoribulokinase, chloroplastic | | K00855|[EC:2.7.1.19] | 0.639 | |  | | | 0.86 | |  | 1.282 | | |  | | | 0.8 | |  | | 1.057 | |  | | | 1.279 | | | |  | |
| B4FRP8 | | Probable plastid-lipid-associated protein 2, chloroplastic | | - | 0.89 | |  | | | 0.7 | |  | 0.743 | | |  | | | 0.8 | |  | | 0.623 | |  | | | 0.842 | | | |  | |
| B6T391 | | Lichenase-2 (Fragment) | | - | 0.45 | |  | | | 0.84 | |  | 1.829 | | |  | | | 0.7 | |  | | 0.833 | |  | | | 1.152 | | | |  | |
| B7ZYT6 | | Ribulose bisphosphate carboxylase small chain, chloroplastic | | K01602|[EC:4.1.1.39] | 1.021 | |  | | | 0.8 | |  | 0.874 | | |  | | | 0.9 | |  | | 0.543 | |  | | | 0.682 | | | |  | |
| Q9XGW8 | | V-type proton ATPase subunit E | | K02150|V-type H+-transporting ATPase subunit E [EC:3.6.3.14] | 0.966 | |  | | | 0.83 | |  | 0.834 | | |  | | | 0.9 | |  | | 1.044 | |  | | | 1.194 | | | |  | |
| B4FNM4 | | 60S acidic ribosomal protein P0 | | K02941|1|2e-154|543|zma:100273601|large subunit ribosomal protein LP0 | 1.17 | |  | | | 1.43 | |  | 1.173 | | |  | | | 1.2 | |  | | 2.625 | |  | | | 2.538 | | | |  | |
| C0PDB0 | | Phosphoglycerate kinase, chloroplastic | | K00927|[EC:2.7.2.3] | 0.908 | |  | | | 0.72 | |  | 0.734 | | |  | | | 1 | |  | | 0.788 | |  | | | 0.848 | | | |  | |
| B6U1D7 | | Sucrose synthase 1 | | K00695|[EC:2.4.1.13] | 2.804 | |  | | | 1.44 | |  | 0.474 | | |  | | | 0.5 | |  | | 1.335 | |  | | | 2.668 | | | |  | |
| B4FFV3 | | Malate dehydrogenase, chloroplastic | | K00026|[EC:1.1.1.37] | 1.716 | |  | | | 0.76 | |  | 0.418 | | |  | | | 1 | |  | | 1.064 | |  | | | 1.208 | | | |  | |
| O81229 | | 60S ribosomal protein L23a | | K02893 | 0.385 | |  | | | 1.38 | |  | 4.001 | | |  | | | 2.2 | |  | | 1.625 | |  | | | 0.843 | | | |  | |
| C5XZH7 | | 50S ribosomal protein L21, chloroplastic | | K02888 | 0.744 | |  | | | 0.91 | |  | 1.297 | | |  | | | 1.3 | |  | | 1.227 | |  | | | 0.896 | | | |  | |
| C0P2M2 | | Phosphoribulokinase, chloroplastic | | K00855|[EC:2.7.1.19] | 0.732 | |  | | | 0.76 | |  | 1.103 | | |  | | | 1.1 | |  | | 0.505 | |  | | | 0.44 | | | |  | |
| B4F848 | | 20 kDa chaperonin, chloroplastic | | K04078|chaperonin GroES | 0.891 | |  | | | 1.27 | |  | 1.286 | | |  | | | 1 | |  | | 1.615 | |  | | | 1.602 | | | |  | |
| C4J030 | | Aspartate aminotransferase P2, mitochondrial (Fragment) | | K00811|[EC:2.6.1.1] | 0.723 | |  | | | 0.62 | |  | 0.924 | | |  | | | 0.9 | |  | | 1.185 | |  | | | 1.347 | | | |  | |
| B4FRH1 | | Thioredoxin M2, chloroplastic | | K03671 | 0.476 | |  | | | 0.75 | |  | 1.555 | | |  | | | 1.6 | |  | | 1.218 | |  | | | 0.794 | | | |  | |
| B4FZU8 | | Malate dehydrogenase, mitochondrial | | K00026|[EC:1.1.1.37] | 0.804 | |  | | | 1.46 | |  | 1.824 | | |  | | | 0.9 | |  | | 0.798 | |  | | | 1.058 | | | |  | |
| B4FEH8 | | Chloroplast stem-loop binding protein of 41 kDa b, chloroplastic | | K01710|dTDP-glucose 4,6-dehydratase [EC:4.2.1.46] | 1.165 | |  | | | 0.95 | |  | 0.851 | | |  | | | 0.6 | |  | | 0.975 | |  | | | 1.66 | | | |  | |
| C5YU58 | | Stromal 70 kDa heat shock-related protein, chloroplastic (Fragment) | | K03283|heat shock 70kDa protein 1/8 | 0.508 | |  | | | 1.2 | |  | 2.031 | | |  | | | 0.6 | |  | | 0.632 | |  | | | 1.028 | | | |  | |
| C0PES7 | | 3'(2'),5'-bisphosphate nucleotidase | | K15422|[EC:3.1.3.7 3.1.3.57] | 1.033 | |  | | | 1.02 | |  | 0.888 | | |  | | | 1.4 | |  | | 1.631 | |  | | | 1.184 | | | |  | |
| C0PEC4 | | 30S ribosomal protein S5, chloroplastic | | K02988 | 0.444 | |  | | | 0.81 | |  | 1.719 | | |  | | | 0.9 | |  | | 1.574 | |  | | | 1.831 | | | |  | |
| Q38JE3 | | Outer membrane lipoprotein blc | | K03098 | 1.528 | |  | | | 2.49 | |  | 1.602 | | |  | | | 0.6 | |  | | 0.839 | |  | | | 1.386 | | | |  | |
| B6TNS8 | | Lichenase-2 (Fragment) | | - | 0.967 | |  | | | 1.27 | |  | 1.255 | | |  | | | 0.8 | |  | | 0.579 | |  | | | 0.776 | | | |  | |
| C5Z0R1 | | Sulfite reductase [ferredoxin], chloroplastic | | K00392|[EC:1.8.7.1] | 1.521 | |  | | | 0.74 | |  | 0.477 | | |  | | | 1.1 | |  | | 0.752 | |  | | | 0.702 | | | |  | |
| Q6RW09 | | Allene oxide cyclase 4, chloroplastic | | K10525|[EC:5.3.99.6] | 2.037 | |  | | | 1.72 | |  | 0.844 | | |  | | | 1.1 | |  | | 0.938 | |  | | | 0.857 | | | |  | |
| B6TVA3 | | Bifunctional chitinase/lysozyme (Fragment) | | K01183|chitinase [EC:3.2.1.14] | 0.937 | |  | | | 0.67 | |  | 0.967 | | |  | | | 0.7 | |  | | 0.483 | |  | | | 0.781 | | | |  | |
| B6TMF0 | | Cystatin-1 | | - | 0.726 | |  | | | 1.24 | |  | 1.626 | | |  | | | 0.8 | |  | | 1.291 | |  | | | 1.689 | | | |  | |
| B8A068 | | S-adenosylmethionine synthase 1 | | K00789|[EC:2.5.1.6] | 1.111 | |  | | | 0.84 | |  | 0.743 | | |  | | | 1 | |  | | 0.727 | |  | | | 0.703 | | | |  | |
| B8A306 | | 2,3-bisphosphoglycerate-independent phosphoglycerate mutase | | K15633|[EC:5.4.2.1] | 1.305 | |  | | | 1.08 | |  | 0.781 | | |  | | | 0.8 | |  | | 0.87 | |  | | | 1.17 | | | |  | |
| B4FH62 | | Uncharacterized protein At2g37660, chloroplastic | | - | 0.539 | |  | | | 0.86 | |  | 1.556 | | |  | | | 0.8 | |  | | 0.944 | |  | | | 1.178 | | | |  | |
| B6TZL0 | | Acyl-CoA-binding protein 2 (Fragment) | | K08762 | 0.175 | |  | | | 1.07 | |  | 6.822 | | |  | | | 2.1 | |  | | 1.811 | |  | | | 0.883 | | | |  | |
| B4FUC4 | | Salt stress root protein RS1 | | - | 0.306 | |  | | | 0.94 | |  | 3.095 | | |  | | | 1.1 | |  | | 1.994 | |  | | | 1.604 | | | |  | |
| B4FHT1 | | Annexin D7 | | - | 1.529 | |  | | | 1.16 | |  | 0.744 | | |  | | | 1.2 | |  | | 2.162 | |  | | | 2.035 | | | |  | |
| C0P5X1 | | Ribulose bisphosphate carboxylase/oxygenase activase, chloroplastic | | - | 0.904 | |  | | | 0.84 | |  | 0.89 | | |  | | | 0.8 | |  | | 1.299 | |  | | | 1.617 | | | |  | |
| B4FRV4 | | Putative lactoylglutathione lyase | | K01759|[EC:4.4.1.5] | 0.811 | |  | | | 0.61 | |  | 0.702 | | |  | | | 0.9 | |  | | 1.097 | |  | | | 1.311 | | | |  | |
| B4FRQ1 | | Ribulose-phosphate 3-epimerase, chloroplastic | | K01783|[EC:5.1.3.1] | 1.481 | |  | | | 0.92 | |  | 0.558 | | |  | | | 0.8 | |  | | 1.078 | |  | | | 1.268 | | | |  | |
| B4FVD5 | | V-type proton ATPase subunit E | | K02150|V-type H+-transporting ATPase subunit E [EC:3.6.3.14] | 1.08 | |  | | | 1.01 | |  | 0.911 | | |  | | | 0.9 | |  | | 0.709 | |  | | | 0.713 | | | |  | |
| B4FT01 | | U2 small nuclear ribonucleoprotein A' | | K11092 | 0.738 | |  | | | 1.06 | |  | 1.082 | | |  | | | 1.1 | |  | | 1.528 | |  | | | 1.426 | | | |  | |
| B6UHU1 | | Catalase isozyme 1 | | K03781|[EC:1.11.1.6] | 1.535 | |  | | | 1.19 | |  | 0.639 | | |  | | | 0.9 | |  | | 0.788 | |  | | | 0.895 | | | |  | |
| B4G1T3 | | Bifunctional chitinase/lysozyme (Fragment) | | K01183|chitinase [EC:3.2.1.14] | 0.689 | |  | | | 0.53 | |  | 0.904 | | |  | | | 1.1 | |  | | 0.27 | |  | | | 0.186 | | | |  | |
| C0HFT8 | | Alpha-glucan water dikinase, chloroplastic | | K08244|[EC:2.7.9.4] | 1.01 | |  | | | 0.99 | |  | 1.08 | | |  | | | 0.8 | |  | | 0.516 | |  | | | 0.625 | | | |  | |
| E1AFV5 | | Glucan endo-1,3-beta-glucosidase GII | | - | 3.144 | |  | | | 2.28 | |  | 0.666 | | |  | | | 0.6 | |  | | 1.674 | |  | | | 2.56 | | | |  | |
| B6TXY3 | | Quinone oxidoreductase-like protein At1g23740, chloroplastic | | K07119|[EC:1.6.5.5] | 0.803 | |  | | | 0.88 | |  | 1.02 | | |  | | | 0.9 | |  | | 1.038 | |  | | | 1.177 | | | |  | |
| B6TGF0 | | Proteasome subunit alpha type-3 | | K02727|20S proteasome subunit alpha 7 [EC:3.4.25.1] | 1.34 | |  | | | 1.37 | |  | 1.013 | | |  | | | 1.2 | |  | | 0.726 | |  | | | 0.733 | | | |  | |
| B4F8T3 | | Spartin | | - | 1.414 | |  | | | 0.97 | |  | 0.758 | | |  | | | 1.1 | |  | | 0.798 | |  | | | 0.826 | | | |  | |
| B7ZXN1 | | Ferritin-1, chloroplastic | | K00522|[EC:1.16.3.1] | 1.801 | |  | | | 0.78 | |  | 0.495 | | |  | | | 1.1 | |  | | 0.855 | |  | | | 0.807 | | | |  | |
| B6TXN0 | | DNA-damage-repair/toleration protein DRT102 | | - | 1.514 | |  | | | 1.11 | |  | 0.842 | | |  | | | 1 | |  | | 1.135 | |  | | | 1.132 | | | |  | |
| B8A3G8 | | Glycine-rich RNA-binding protein 1 (Fragment) | | K13195|cold-inducible RNA-binding protein!  K02965|small subunit ribosomal protein S19!  K11294|nucleolin!  K12741|heterogeneous nuclear ribonucleoprotein A1/A3 | 0.623 | |  | | | 0.87 | |  | 1.341 | | |  | | | 1.2 | |  | | 0.915 | |  | | | 0.777 | | | |  | |
| B4F836 | | Probable lactoylglutathione lyase, chloroplast | | K01759|[EC:4.4.1.5] | 1.11 | |  | | | 0.78 | |  | 0.631 | | |  | | | 1.1 | |  | | 1.353 | |  | | | 1.282 | | | |  | |
| C5XR33 | | Phosphoglucomutase, chloroplastic | | K01835|[EC:5.4.2.2] | 1.446 | |  | | | 0.91 | |  | 0.567 | | |  | | | 1.1 | |  | | 1.032 | |  | | | 0.97 | | | |  | |
| B4FSJ3 | | Ribulose bisphosphate carboxylase small chain, chloroplastic | | K01602|[EC:4.1.1.39] | 1.084 | |  | | | 0.72 | |  | 0.659 | | |  | | | 1.2 | |  | | 1.224 | |  | | | 1.12 | | | |  | |
| B6TP60 | | Glycine-rich protein 2 | | K09250|cellular nucleic acid-binding protein | 1.264 | |  | | | 1.15 | |  | 0.925 | | |  | | | 1.4 | |  | | 1.211 | |  | | | 1.067 | | | |  | |
| B3SHC9 | | ATP synthase subunit beta, chloroplastic O | | K02112|F-type H+-transporting ATPase subunit beta [EC:3.6.3.14] | 0.793 | |  | | | 1.21 | |  | 1.244 | | |  | | | 0.9 | |  | | 0.868 | |  | | | 0.969 | | | |  | |
| B6U0V6 | | Endoplasmin homolog | | K09487heat shock protein 90kDa beta | 1.402 | |  | | | 0.71 | |  | 0.579 | | |  | | | 0.8 | |  | | 0.452 | |  | | | 0.58 | | | |  | |
| C4J6X8 | | Stromal 70 kDa heat shock-related protein, chloroplastic (Fragment) | | K03283heat shock 70kDa protein 1/8 | 0.508 | |  | | | 0.96 | |  | 1.709 | | |  | | | 0.9 | |  | | 1.11 | |  | | | 1.159 | | | |  | |
| C0HFW7 | | Unknown protein DS12 from 2D-PAGE of leaf, chloroplastic | | - | 0.835 | |  | | | 0.7 | |  | 0.907 | | |  | | | 1.3 | |  | | 2.036 | |  | | | 1.835 | | | |  | |
| **Cadmium** | |  | |  |  | |  | | |  | |  |  | | |  | | |  | |  | |  | |  | | |  | | | |  | |
| B6TNC0 | | Ankyrin repeat domain-containing protein 2 | | K06694|26S proteasome non-ATPase regulatory subunit 10!  K12489|Arf-GAP with coiled-coil, ANK repeat and PH domain-containing protein | 0.343 | |  | | | 1.16 | |  | 4.291 | | |  | | | 1.2 | |  | | 0.577 | |  | | | 0.641 | | | |  | |
| C4JA47 | | Elongation factor 1-alpha | | K03231 | 0.798 | |  | | | 1.13 | |  | 1.548 | | |  | | | 0.9 | |  | | 0.767 | |  | | | 0.816 | | | |  | |
| B4G0L9 | | Putative uncharacterized protein | | K00134|glyceraldehyde 3-phosphate dehydrogenase [EC:1.2.1.12] | 2.079 | |  | | | 2.08 | |  | 0.85 | | |  | | | 0.7 | |  | | 0.969 | |  | | | 1.111 | | | |  | |
| B4FK49 | | Nucleoside diphosphate kinase 1 | | K00940|[EC:2.7.4.6] | 1.302 | |  | | | 0.9 | |  | 0.775 | | |  | | | 0.8 | |  | | 1.064 | |  | | | 1.351 | | | |  | |
| C5X6E9 | | 26S protease regulatory subunit 4 homolog | | K03062 | 1.506 | |  | | | 1.11 | |  | 0.682 | | |  | | | 1 | |  | | 0.957 | |  | | | 1.102 | | | |  | |
| B4FZZ2 | | Peptidyl-prolyl cis-trans isomerase | | K01802|[EC:5.2.1.8] | 1.483 | |  | | | 2.72 | |  | 1.8 | | |  | | | 0.8 | |  | | 1.067 | |  | | | 1.228 | | | |  | |
| B4FL17 | | Translationally-controlled tumor protein homolog | | - | 1.584 | |  | | | 1.02 | |  | 0.643 | | |  | | | 1.2 | |  | | 1.451 | |  | | | 1.299 | | | |  | |
| B4FVJ9 | | Probable glutathione S-transferase GSTU1 | | K00799|[EC:2.5.1.18] | 1.999 | |  | | | 0.55 | |  | 0.273 | | |  | | | 1.4 | |  | | 2.173 | |  | | | 1.549 | | | |  | |
| B8A2P6 | | Tudor domain-containing protein 1 | | K15979|staphylococcal nuclease domain-containing protein 1 | 1.564 | |  | | | 1.22 | |  | 0.705 | | |  | | | 0.8 | |  | | 0.538 | |  | | | 1.005 | | | |  | |
| Q43274 | | Aldehyde dehydrogenase family 2 member B7, mitochondrial | | K00128|[EC:1.2.1.3] | 1.159 | |  | | | 0.73 | |  | 0.619 | | |  | | | 1 | |  | | 0.791 | |  | | | 0.799 | | | |  | |
| B4FW57 | | Oligopeptidase A | | K01414|[EC:3.4.24.70] | 1.694 | |  | | | 1.04 | |  | 0.698 | | |  | | | 1 | |  | | 1.06 | |  | | | 1.206 | | | |  | |
| C0P732 | | Heat shock protein STI | | K09553|stress-induced-phosphoprotein 1 | 0.347 | |  | | | 1 | |  | 2.617 | | |  | | | 0.8 | |  | | 1.234 | |  | | | 1.51 | | | |  | |
| B6TPI3 | | Thioredoxin reductase NTRA | | K00384|[EC:1.8.1.9] | 0.622 | |  | | | 0.76 | |  | 1.37 | | |  | | | 0.8 | |  | | 0.806 | |  | | | 0.625 | | | |  | |
| B4FI86 | | Proteasome subunit beta type-5-A | | K02737|[EC:3.4.25.1] | 1.509 | |  | | | 1.15 | |  | 0.668 | | |  | | | 0.9 | |  | | 0.882 | |  | | | 1.054 | | | |  | |
| C4J6Q3 | | Citrate synthase, mitochondrial | | K01647|[EC:2.3.3.1] | 2.133 | |  | | | 1 | |  | 0.396 | | |  | | | 1 | |  | | 0.508 | |  | | | 0.557 | | | |  | |
| B4FNW1 | | Triosephosphate isomerase, cytosolic | | K01803|[EC:5.3.1.1] | 1.937 | |  | | | 1.45 | |  | 0.762 | | |  | | | 0.9 | |  | | 1.636 | |  | | | 1.692 | | | |  | |
| B4FRH5 | | Succinyl-CoA ligase [ADP-forming] subunit beta, mitochondrial | | K01900|[EC:6.2.1.4 6.2.1.5] | 1.788 | |  | | | 1.1 | |  | 0.662 | | |  | | | 0.9 | |  | | 1.341 | |  | | | 1.571 | | | |  | |
| C5WSS5 | | Probable methionine--tRNA ligase | | K01874|[EC:6.1.1.10] | 1.367 | |  | | | 1.39 | |  | 1.099 | | |  | | | 1.2 | |  | | 1.558 | |  | | | 1.321 | | | |  | |
| B4FG03 | | Alanine aminotransferase 2 | | K00814|[EC:2.6.1.2] | 0.683 | |  | | | 0.58 | |  | 0.75 | | |  | | | 0.9 | |  | | 1.412 | |  | | | 1.48 | | | |  | |
| C0HHU2 | | 2,3-bisphosphoglycerate-independent phosphoglycerate mutase | | K15633|[EC:5.4.2.1] | 1.654 | |  | | | 1.17 | |  | 0.694 | | |  | | | 0.8 | |  | | 0.865 | |  | | | 1.111 | | | |  | |
| C0PDB0 | | Phosphoglycerate kinase, chloroplastic | | K00927|[EC:2.7.2.3] | 0.789 | |  | | | 0.67 | |  | 0.907 | | |  | | | 0.9 | |  | | 0.803 | |  | | | 0.917 | | | |  | |
| C5X2E3 | | Alanine aminotransferase 2 | | K00814|[EC:2.6.1.2] | 1.263 | |  | | | 0.84 | |  | 0.571 | | |  | | | 0.9 | |  | | 0.79 | |  | | | 0.942 | | | |  | |
| B6TBZ8 | | Alanine aminotransferase 2 | | K00814|[EC:2.6.1.2] | 1.269 | |  | | | 0.72 | |  | 0.548 | | |  | | | 1 | |  | | 1.187 | |  | | | 1.217 | | | |  | |
| B4FQM2 | | Pyrophosphate--fructose 6-phosphate 1-phosphotransferase subunit beta | | K00895|[EC:2.7.1.90] | 3.061 | |  | | | 1.88 | |  | 0.604 | | |  | | | 1.1 | |  | | 2.023 | |  | | | 1.839 | | | |  | |
| C0P3L1 | | Phosphoenolpyruvate carboxykinase [ATP] | | K01610|[EC:4.1.1.49] | 1.815 | |  | | | 0.95 | |  | 0.583 | | |  | | | 0.8 | |  | | 0.665 | |  | | | 0.802 | | | |  | |
| B4F8L7 | | Glyceraldehyde-3-phosphate dehydrogenase B, chloroplastic (Fragment) | | K05298|[EC:1.2.1.13] | 0.796 | |  | | | 0.76 | |  | 0.938 | | |  | | | 1.1 | |  | | 0.696 | |  | | | 0.635 | | | |  | |
| B4FTW9 | | Adenylosuccinate synthetase, chloroplastic | | K01939|[EC:6.3.4.4] | 1.377 | |  | | | 1.09 | |  | 0.771 | | |  | | | 0.9 | |  | | 1.28 | |  | | | 1.299 | | | |  | |
| B8A021 | | Phospholipase D alpha 1 | | K01115|[EC:3.1.4.4] | 1.743 | |  | | | 1.13 | |  | 0.572 | | |  | | | 1.1 | |  | | 0.892 | |  | | | 0.775 | | | |  | |
| B4FID4 | | Aldo-keto reductase family 4 member C9 | | K00002|alcohol dehydrogenase (NADP+) [EC:1.1.1.2]!  K00011|aldehyde reductase [EC:1.1.1.21]!  K00085|[EC:1.1.1.200]!  K08243|6'-deoxychalcone synthase [EC:2.3.1.170] | 0.752 | |  | | | 1.06 | |  | 2.032 | | |  | | | 0.9 | |  | | 1.179 | |  | | | 1.077 | | | |  | |
| B4FPA1 | | Ubiquitin-conjugating enzyme E2 36 | | K10580|[EC:6.3.2.19] | 1.009 | |  | | | 1.23 | |  | 0.954 | | |  | | | 0.9 | |  | | 1.208 | |  | | | 1.582 | | | |  | |
| B5AMJ8 | | Alpha-glucan phosphorylase, H isozyme | | K00688|starch phosphorylase [EC:2.4.1.1] | 2.287 | |  | | | 1.44 | |  | 0.607 | | |  | | | 1.3 | |  | | 0.885 | |  | | | 0.788 | | | |  | |
| C4J1J9 | | Elongation factor Ts | | K02357 | 0.621 | |  | | | 1.14 | |  | 1.903 | | |  | | | 0.9 | |  | | 1.614 | |  | | | 1.529 | | | |  | |
| C4J1W2 | | NADP-dependent malic enzyme, chloroplastic | | K00029|[EC:1.1.1.40] | 0.979 | |  | | | 1.27 | |  | 1.275 | | |  | | | 0.6 | |  | | 0.489 | |  | | | 0.798 | | | |  | |
| B4FQT3 | | Heat shock protein STI | | K09553|stress-induced-phosphoprotein 1 | 0.524 | |  | | | 1.36 | |  | 2.965 | | |  | | | 1.1 | |  | | 0.924 | |  | | | 0.969 | | | |  | |
| B4FW90 | | Luminal-binding protein 2 | | K09490|heat shock 70kDa protein 5 | 1.396 | |  | | | 1.25 | |  | 0.819 | | |  | | | 0.7 | |  | | 0.753 | |  | | | 1.094 | | | |  | |
| C0P723 | | Probable succinyl-CoA ligase [ADP-forming] subunit alpha, mitochondrial | | K01899|[EC:6.2.1.4 6.2.1.5] | 1.506 | |  | | | 1.23 | |  | 0.926 | | |  | | | 0.7 | |  | | 1.178 | |  | | | 1.727 | | | |  | |
| Q5EUD7 | | Protein disulfide isomerase-like 2-2 | | K01829|[EC:5.3.4.1]!  K09584|protein disulfide-isomerase A6 [EC:5.3.4.1] | 1.114 | |  | | | 1.54 | |  | 1.232 | | |  | | | 1 | |  | | 1.526 | |  | | | 1.644 | | | |  | |
| B4FR08 | | Cysteine synthase, chloroplastic/chromoplastic | | K01738|[EC:2.5.1.47] | 0.83 | |  | | | 1.13 | |  | 1.387 | | |  | | | 0.8 | |  | | 0.92 | |  | | | 1.13 | | | |  | |
| C5Y6F3 | | Proteasome subunit alpha type-5 | | K02729|20S proteasome subunit alpha 5 [EC:3.4.25.1] | 1.364 | |  | | | 1.21 | |  | 0.994 | | |  | | | 1.1 | |  | | 1.326 | |  | | | 1.092 | | | |  | |
| B4G177 | | Transketolase, chloroplastic | | K00615|[EC:2.2.1.1] | 1.021 | |  | | | 1.17 | |  | 1.098 | | |  | | | 0.9 | |  | | 1.032 | |  | | | 1.219 | | | |  | |
| B7ZZJ4 | | Heat shock 70 kDa protein 14 | | K09489 | 1.433 | |  | | | 1.58 | |  | 1.056 | | |  | | | 1 | |  | | 0.961 | |  | | | 1.089 | | | |  | |
| C0PHE3 | | Eukaryotic initiation factor 4A-3 | | K03257|translation initiation factor 4A | 2.5 | |  | | | 0.85 | |  | 0.396 | | |  | | | 1.2 | |  | | 0.836 | |  | | | 0.73 | | | |  | |
| B4FNM4 | | 60S acidic ribosomal protein P0 | | K02941|large subunit ribosomal protein LP0 | 1.17 | |  | | | 1.43 | |  | 1.173 | | |  | | | 1.2 | |  | | 2.625 | |  | | | 2.538 | | | |  | |
| Q8W4W3 | | Glutamate--cysteine ligase B, chloroplastic | | K01919|[EC:6.3.2.2] | 1.586 | |  | | | 0.9 | |  | 0.53 | | |  | | | 1.1 | |  | | 1.362 | |  | | | 1.087 | | | |  | |
| C0PDB0 | | Phosphoglycerate kinase, chloroplastic | | K00927|[EC:2.7.2.3] | 0.908 | |  | | | 0.72 | |  | 0.734 | | |  | | | 1 | |  | | 0.788 | |  | | | 0.848 | | | |  | |
| B6SIL7 | | Eukaryotic translation initiation factor 5A | | K03263 | 0.765 | |  | | | 0.93 | |  | 1.165 | | |  | | | 1.1 | |  | | 1.638 | |  | | | 1.413 | | | |  | |
| B6TPH0 | | Lactoylglutathione lyase | | K01759|[EC:4.4.1.5] | 1.4 | |  | | | 1.06 | |  | 0.719 | | |  | | | 0.9 | |  | | 1.668 | |  | | | 1.897 | | | |  | |
| B6U1D7 | | Sucrose synthase 1 | | K00695|[EC:2.4.1.13] | 2.804 | |  | | | 1.44 | |  | 0.474 | | |  | | | 0.5 | |  | | 1.335 | |  | | | 2.668 | | | |  | |
| O82443 | | Alanine aminotransferase 2 | | K00814|[EC:2.6.1.2] | 1.423 | |  | | | 0.98 | |  | 0.708 | | |  | | | 0.8 | |  | | 0.826 | |  | | | 1.041 | | | |  | |
| B4FUE0 | | Obg-like ATPase 1 | | K06942 | 1.544 | |  | | | 1.05 | |  | 0.654 | | |  | | | 0.9 | |  | | 0.925 | |  | | | 1.112 | | | |  | |
| B6TQ06 | | Aminomethyltransferase, mitochondrial | | K00605|[EC:2.1.2.10] | 1.256 | |  | | | 1.03 | |  | 0.601 | | |  | | | 1 | |  | | 0.872 | |  | | | 1.054 | | | |  | |
| B4G0S0 | | Uridine 5'-monophosphate synthase (Fragment) | | K13421|[EC:2.4.2.10 4.1.1.23] | 2.647 | |  | | | 1.64 | |  | 0.848 | | |  | | | 0.9 | |  | | 0.95 | |  | | | 1.291 | | | |  | |
| C0PB80 | | Chaperonin CPN60-1, mitochondrial | | K04077|chaperonin GroEL | 0.982 | |  | | | 0.8 | |  | 0.846 | | |  | | | 0.9 | |  | | 1.804 | |  | | | 1.934 | | | |  | |
| C5YTZ0 | | Alanine--tRNA ligase | | K01872|[EC:6.1.1.7] | 1.401 | |  | | | 0.91 | |  | 0.737 | | |  | | | 0.8 | |  | | 0.966 | |  | | | 1.016 | | | |  | |
| C5YS41 | | Glycine dehydrogenase [decarboxylating], mitochondrial | | K00281|[EC:1.4.4.2] | 1.186 | |  | | | 0.88 | |  | 0.698 | | |  | | | 0.8 | |  | | 0.745 | |  | | | 0.988 | | | |  | |
| B4FRC9 | | Transaldolase 1 | | K00616|[EC:2.2.1.2] | 1.839 | |  | | | 0.78 | |  | 0.415 | | |  | | | 1.5 | |  | | 1.109 | |  | | | 0.783 | | | |  | |
| B4FS03 | | Adenine phosphoribosyltransferase 1 | | K00759|[EC:2.4.2.7] | 2.063 | |  | | | 0.82 | |  | 0.564 | | |  | | | 0.9 | |  | | 0.836 | |  | | | 0.921 | | | |  | |
| B4FD74 | | Isoflavone reductase homolog IRL | | K00224| [EC:1.3.1.-]!  K13081|leucoanthocyanidin reductase [EC:1.17.1.3] | 2.333 | |  | | | 3.46 | |  | 1.474 | | |  | | | 1.1 | |  | | 0.789 | |  | | | 0.751 | | | |  | |
| C0HHT0 | | Phospholipase D alpha 1 | | K01115|[EC:3.1.4.4] | 1.766 | |  | | | 1 | |  | 0.488 | | |  | | | 1.3 | |  | | 1.114 | |  | | | 0.95 | | | |  | |
| B4F8P6 | | NADP-dependent malic enzyme, chloroplastic | | K00029|malate dehydrogenase (oxaloacetate-decarboxylating)(NADP+) [EC:1.1.1.40] | 1.131 | |  | | | 0.66 | |  | 0.597 | | |  | | | 1 | |  | | 0.701 | |  | | | 0.703 | | | |  | |
| Q8L8G5 | | Nucleosome assembly protein 1-like 1 | | K11279 | 0.387 | |  | | | 1.1 | |  | 2.357 | | |  | | | 1.2 | |  | | 1.484 | |  | | | 1.287 | | | |  | |
| B7ZWY9 | | Citrate synthase 4, mitochondrial | | K01647|[EC:2.3.3.1] | 1.478 | |  | | | 0.73 | |  | 0.461 | | |  | | | 0.8 | |  | | 0.563 | |  | | | 0.697 | | | |  | |
| C0PCC7 | | Glutamate decarboxylase 1 | | K01580|[EC:4.1.1.15] | 3.82 | |  | | | 1.56 | |  | 0.444 | | |  | | | 0.9 | |  | | 0.928 | |  | | | 1.057 | | | |  | |
| B4F848 | | 20 kDa chaperonin, chloroplastic | | K04078|chaperonin GroES | 0.891 | |  | | | 1.27 | |  | 1.286 | | |  | | | 1 | |  | | 1.615 | |  | | | 1.602 | | | |  | |
| B6SVU1 | | Asparagine--tRNA ligase, cytoplasmic 3 | | K01893|[EC:6.1.1.22] | 1.509 | |  | | | 0.9 | |  | 0.554 | | |  | | | 0.8 | |  | | 0.829 | |  | | | 1.003 | | | |  | |
| B6T2Y5 | | S-formylglutathione hydrolase | | K01070|[EC:3.1.2.12] | 1.542 | |  | | | 1.17 | |  | 0.752 | | |  | | | 0.9 | |  | | 0.943 | |  | | | 1.078 | | | |  | |
| B4FN24 | | Peroxiredoxin-2C | | K03386| [EC:1.11.1.15]!  K11187|peroxiredoxin 5, atypical 2-Cys peroxiredoxin [EC:1.11.1.15] | 0.961 | |  | | | 0.76 | |  | 0.849 | | |  | | | 1 | |  | | 1.252 | |  | | | 1.276 | | | |  | |
| B4FS18 | | L-ascorbate peroxidase 1, cytosolic | | K00434|[EC:1.11.1.11] | 1.354 | |  | | | 3.06 | |  | 2.304 | | |  | | | 0.8 | |  | | 0.933 | |  | | | 1.227 | | | |  | |
| B8A2P6 | | Tudor domain-containing protein 1 | | K15979|staphylococcal nuclease domain-containing protein 1 | 1.177 | |  | | | 1.02 | |  | 0.927 | | |  | | | 0.8 | |  | | 0.645 | |  | | | 0.86 | | | |  | |
| B4FAL9 | | Fructose-bisphosphate aldolase, cytoplasmic isozyme | | K01623| [EC:4.1.2.13] | 1.746 | |  | | | 1.72 | |  | 1.011 | | |  | | | 0.8 | |  | | 1.047 | |  | | | 1.26 | | | |  | |
| B4FLN7 | | Transcription factor Pur-alpha 1 | | - | 0.889 | |  | | | 1.36 | |  | 1.502 | | |  | | | 0.8 | |  | | 1.058 | |  | | | 1.132 | | | |  | |
| B4FBF4 | | Serine hydroxymethyltransferase 1 | | K00600|glycine hydroxymethyltransferase [EC:2.1.2.1] | 1.771 | |  | | | 1.19 | |  | 0.603 | | |  | | | 1.2 | |  | | 0.871 | |  | | | 0.711 | | | |  | |
| B6SGV9 | | Lactoylglutathione lyase | | K01759|[EC:4.4.1.5] | 1.081 | |  | | | 0.96 | |  | 0.833 | | |  | | | 1.2 | |  | | 1.375 | |  | | | 1.1 | | | |  | |
| B6SVV9 | | ATP synthase subunit beta, mitochondrial | | K02133|F-type H+-transporting ATPase subunit beta [EC:3.6.3.14] | 1.546 | |  | | | 0.77 | |  | 0.521 | | |  | | | 1 | |  | | 1.251 | |  | | | 1.227 | | | |  | |
| C4J030 | | Aspartate aminotransferase P2, mitochondrial (Fragment) | | K00811|[EC:2.6.1.1] | 0.723 | |  | | | 0.62 | |  | 0.924 | | |  | | | 0.9 | |  | | 1.185 | |  | | | 1.347 | | | |  | |
| B6TLA2 | | Thioredoxin reductase NTRB | | K00384|[EC:1.8.1.9] | 0.917 | |  | | | 0.82 | |  | 0.933 | | |  | | | 0.8 | |  | | 0.993 | |  | | | 1.254 | | | |  | |
| C5YHN2 | | Glycine--tRNA ligase 1, mitochondrial | | K01880|[EC:6.1.1.14] | 1.389 | |  | | | 1.11 | |  | 0.847 | | |  | | | 0.9 | |  | | 0.969 | |  | | | 0.995 | | | |  | |
| B6TVJ4 | | Thioredoxin H-type | | K03671 | 1.373 | |  | | | 1.23 | |  | 0.784 | | |  | | | 1.1 | |  | | 3.309 | |  | | | 3.406 | | | |  | |
| B4F8B4 | | Putative monooxygenase Rv1533 | | - | 1.642 | |  | | | 0.81 | |  | 0.514 | | |  | | | 0.9 | |  | | 0.987 | |  | | | 1.17 | | | |  | |
| B4FZU8 | | Malate dehydrogenase, mitochondrial | | K00026|[EC:1.1.1.37] | 0.804 | |  | | | 1.46 | |  | 1.824 | | |  | | | 0.9 | |  | | 0.798 | |  | | | 1.058 | | | |  | |
| C4J0N7 | | Oryzain alpha chain | | K01376| [EC:3.4.22.-]!  K01365|cathepsin L [EC:3.4.22.15] | 0.863 | |  | | | 0.98 | |  | 1.113 | | |  | | | 0.8 | |  | | 0.505 | |  | | | 0.663 | | | |  | |
| B4FFH8 | | Adenosine kinase 1 | | K00856|[EC:2.7.1.20] | 2.387 | |  | | | 1.76 | |  | 0.738 | | |  | | | 1.1 | |  | | 1.027 | |  | | | 0.916 | | | |  | |
| B6T7G7 | | Elongation factor 1-gamma 3 | | K03233 | 1.371 | |  | | | 1.06 | |  | 0.81 | | |  | | | 0.8 | |  | | 0.959 | |  | | | 1.238 | | | |  | |
| A5JVZ6 | | Photosystem II stability/assembly factor HCF136, chloroplastic | | - | 1.034 | |  | | | 0.88 | |  | 0.851 | | |  | | | 0.9 | |  | | 0.856 | |  | | | 0.976 | | | |  | |
| B4FV43 | | RutC family protein jhp_0879 | | - | 1.042 | |  | | | 1.43 | |  | 1.28 | | |  | | | 1.2 | |  | | 1.548 | |  | | | 1.133 | | | |  | |
| B4FVC7 | | Nitrilase homolog 1 | | K01506| [EC:3.5.-.-]!  K13566|omega-amidase [EC:3.5.1.3] | 1.319 | |  | | | 0.9 | |  | 0.685 | | |  | | | 1.2 | |  | | 1.134 | |  | | | 0.971 | | | |  | |
| C0HF19 | | Nucleosome assembly protein 1-like 1 | | K11279 | 0.978 | |  | | | 1.1 | |  | 1.272 | | |  | | | 1.9 | |  | | 1.933 | |  | | | 1.138 | | | |  | |
| B6TB10 | | Protein IN2-1 homolog B | | K00799|glutathione S-transferase [EC:2.5.1.18] | 1.314 | |  | | | 0.67 | |  | 0.533 | | |  | | | 1 | |  | | 1.749 | |  | | | 1.776 | | | |  | |
| B4FSJ2 | | Elongation factor Tu, mitochondrial | | K02358 | 1.355 | |  | | | 0.86 | |  | 0.625 | | |  | | | 0.9 | |  | | 1.301 | |  | | | 1.301 | | | |  | |
| C5YU58 | | Stromal 70 kDa heat shock-related protein, chloroplastic (Fragment) | | K03283|heat shock 70kDa protein 1/8 | 0.508 | |  | | | 1.2 | |  | 2.031 | | |  | | | 0.6 | |  | | 0.632 | |  | | | 1.028 | | | |  | |
| B4FWP0 | | Fructose-bisphosphate aldolase, cytoplasmic isozyme | | K01623|[EC:4.1.2.13] | 2.043 | |  | | | 1.56 | |  | 0.677 | | |  | | | 0.9 | |  | | 1.237 | |  | | | 1.496 | | | |  | |
| B6T7D4 | | Fructose-bisphosphate aldolase, chloroplastic | | K01623|[EC:4.1.2.13] | 0.927 | |  | | | 0.72 | |  | 0.804 | | |  | | | 1.2 | |  | | 0.726 | |  | | | 0.588 | | | |  | |
| C7E3V7 | | Peptidyl-prolyl cis-trans isomerase | | K01802|[EC:5.2.1.8] | 0.904 | |  | | | 1.26 | |  | 1.433 | | |  | | | 0.5 | |  | | 0.734 | |  | | | 1.37 | | | |  | |
| C0PEC4 | | 30S ribosomal protein S5, chloroplastic | | K02988 | 0.444 | |  | | | 0.81 | |  | 1.719 | | |  | | | 0.9 | |  | | 1.574 | |  | | | 1.831 | | | |  | |
| C5X255 | | Formate--tetrahydrofolate ligase | | K01938|[EC:6.3.4.3] | 1.388 | |  | | | 1.08 | |  | 0.82 | | |  | | | 0.9 | |  | | 0.923 | |  | | | 0.974 | | | |  | |
| C0PC62 | | Ketol-acid reductoisomerase, chloroplastic | | K00053|[EC:1.1.1.86] | 2.917 | |  | | | 1.83 | |  | 0.602 | | |  | | | 1.1 | |  | | 1.014 | |  | | | 1.055 | | | |  | |
| C5X0G5 | | Cell division control protein 48 homolog E | | K13525|transitional endoplasmic reticulum ATPase | 0.887 | |  | | | 0.91 | |  | 0.805 | | |  | | | 1 | |  | | 0.876 | |  | | | 0.901 | | | |  | |
| C0PM74 | | Adenylate kinase B | | K00939|[EC:2.7.4.3] | 0.72 | |  | | | 1.22 | |  | 1.66 | | |  | | | 1.5 | |  | | 1.381 | |  | | | 0.976 | | | |  | |
| B6TRQ7 | | U-box domain-containing protein 72 | | K10599|pre-mRNA-processing factor 19 [EC:6.3.2.19] | 1.375 | |  | | | 0.79 | |  | 0.566 | | |  | | | 0.8 | |  | | 1.107 | |  | | | 1.34 | | | |  | |
| B6TX10 | | 6-phosphogluconate dehydrogenase, decarboxylating | | K00033|[EC:1.1.1.44] | 1.282 | |  | | | 0.96 | |  | 0.636 | | |  | | | 1.1 | |  | | 0.477 | |  | | | 0.526 | | | |  | |
| B6TFS9 | | 14-3-3-like protein GF14-A | | K06630|tyrosine 3-monooxygenase/tryptophan 5-monooxygenase activation protein | 1.542 | |  | | | 0.98 | |  | 0.719 | | |  | | | 1.2 | |  | | 0.949 | |  | | | 0.737 | | | |  | |
| B6SKA7 | | Elongation factor 1-alpha | | K03231 | 0.654 | |  | | | 1.28 | |  | 2.099 | | |  | | | 1 | |  | | 0.815 | |  | | | 0.821 | | | |  | |
| C0PKG2 | | UDP-sugar pyrophosphorylase | | K12447|[EC:2.7.7.64] | 0.852 | |  | | | 0.94 | |  | 0.859 | | |  | | | 0.9 | |  | | 1.127 | |  | | | 1.312 | | | |  | |
| C0P4T5 | | Aspartate--tRNA ligase, cytoplasmic | | K01876|[EC:6.1.1.12] | 1.477 | |  | | | 1.11 | |  | 0.868 | | |  | | | 1.1 | |  | | 0.776 | |  | | | 0.744 | | | |  | |
| B4FHH4 | | Triosephosphate isomerase, cytosolic | | K01803|[EC:5.3.1.1] | 1.103 | |  | | | 1.01 | |  | 0.894 | | |  | | | 1 | |  | | 1.933 | |  | | | 1.992 | | | |  | |
| C5XSS8 | | Presequence protease 2, chloroplastic/mitochondrial | | K06972 | 1.213 | |  | | | 0.7 | |  | 0.55 | | |  | | | 1.2 | |  | | 1.437 | |  | | | 1.409 | | | |  | |
| O24449 | | Eukaryotic initiation factor 4A-3 | | K03257 | 1.395 | |  | | | 0.84 | |  | 0.565 | | |  | | | 1.2 | |  | | 0.918 | |  | | | 0.834 | | | |  | |
| B8A306 | | 2,3-bisphosphoglycerate-independent phosphoglycerate mutase | | K15633|[EC:5.4.2.1] | 1.305 | |  | | | 1.08 | |  | 0.781 | | |  | | | 0.8 | |  | | 0.87 | |  | | | 1.17 | | | |  | |
| B8A161 | | Nucleosome assembly protein 1-like 2 | | K11279 | 0.55 | |  | | | 0.98 | |  | 1.626 | | |  | | | 1.2 | |  | | 1.303 | |  | | | 1.04 | | | |  | |
| B4FAD9 | | UTP--glucose-1-phosphate uridylyltransferase | | K00963|[EC:2.7.7.9] | 1.663 | |  | | | 0.92 | |  | 0.575 | | |  | | | 1 | |  | | 1.466 | |  | | | 1.58 | | | |  | |
| B4FUN3 | | Protein IN2-1 homolog B | | K00799|glutathione S-transferase [EC:2.5.1.18] | 1.297 | |  | | | 1.25 | |  | 1.045 | | |  | | | 2 | |  | | 1.673 | |  | | | 0.815 | | | |  | |
| B6TMX0 | | Pyruvate kinase, cytosolic isozyme | | K00873|[EC:2.7.1.40] | 2.601 | |  | | | 1.49 | |  | 0.831 | | |  | | | 1 | |  | | 0.808 | |  | | | 0.919 | | | |  | |
| B6TVG1 | | NADP-dependent malic enzyme, chloroplastic | | K00029|malate dehydrogenase (oxaloacetate-decarboxylating)(NADP+) [EC:1.1.1.40] | 2.811 | |  | | | 1.09 | |  | 0.472 | | |  | | | 1.1 | |  | | 1.686 | |  | | | 1.54 | | | |  | |
| B4FCY0 | | Elongation factor 1-gamma 1 | | K03233 | 1.41 | |  | | | 1.03 | |  | 0.58 | | |  | | | 1 | |  | | 1.184 | |  | | | 1.231 | | | |  | |
| Q5GAU1 | | Alanine aminotransferase 2 | | K00814|[EC:2.6.1.2] | 1.165 | |  | | | 1.04 | |  | 0.78 | | |  | | | 0.7 | |  | | 0.659 | |  | | | 0.873 | | | |  | |
| B4FRR1 | | Soluble inorganic pyrophosphatase 1, chloroplastic | | K01507|inorganic pyrophosphatase [EC:3.6.1.1] | 0.208 | |  | | | 1.1 | |  | 5.285 | | |  | | | 0.8 | |  | | 1.591 | |  | | | 1.956 | | | |  | |
| B4FUC4 | | Salt stress root protein RS1 | | - | 0.306 | |  | | | 0.94 | |  | 3.095 | | |  | | | 1.1 | |  | | 1.994 | |  | | | 1.604 | | | |  | |
| B6SHX8 | | Copper transport protein ATOX1 | | K07213 | 0.434 | |  | | | 1.78 | |  | 3.793 | | |  | | | 0.9 | |  | | 0.692 | |  | | | 0.948 | | | |  | |
| O50018 | | Elongation factor 1-alpha | | K03231 | 1.014 | |  | | | 1.36 | |  | 1.305 | | |  | | | 1.1 | |  | | 0.997 | |  | | | 0.885 | | | |  | |
| B4FRR1 | | Soluble inorganic pyrophosphatase 1, chloroplastic | | K01507|inorganic pyrophosphatase [EC:3.6.1.1] | 0.338 | |  | | | 0.83 | |  | 2.725 | | |  | | | 1 | |  | | 1.441 | |  | | | 1.418 | | | |  | |
| C4J4W3 | | Heat shock protein STI | | K09553|stress-induced-phosphoprotein 1 | 0.389 | |  | | | 1.1 | |  | 2.419 | | |  | | | 1.2 | |  | | 1.403 | |  | | | 1.317 | | | |  | |
| B4FIC0 | | Citrate synthase 4, mitochondrial | | K01647|1|0.0|941|zma:100194338|citrate synthase [EC:2.3.3.1] | 2.149 | |  | | | 1.21 | |  | 0.608 | | |  | | | 0.9 | |  | | 0.967 | |  | | | 1.183 | | | |  | |
| B4FLU9 | | Heat shock 70 kDa protein 14 | | K09489 | 1.45 | |  | | | 1.11 | |  | 0.831 | | |  | | | 0.9 | |  | | 0.836 | |  | | | 0.914 | | | |  | |
| C0P5Y3 | | 5-methyltetrahydropteroyltriglutamate--homocysteine methyltransferase | | K00549|[EC:2.1.1.14] | 1.787 | |  | | | 1.09 | |  | 0.576 | | |  | | | 1.2 | |  | | 0.955 | |  | | | 0.713 | | | |  | |
| C4J5G3 | | Dihydrolipoyl dehydrogenase 1, mitochondrial | | K00382|[EC:1.8.1.4] | 1.374 | |  | | | 1.06 | |  | 0.783 | | |  | | | 0.8 | |  | | 0.779 | |  | | | 1.094 | | | |  | |
| B6TVM7 | | 4-methyl-5-thiazole monophosphate biosynthesis protein | | K03152 | 1.348 | |  | | | 1.14 | |  | 0.866 | | |  | | | 1 | |  | | 1.006 | |  | | | 1.142 | | | |  | |
| C0PD27 | | Isocitrate dehydrogenase [NADP] | | K00031|[EC:1.1.1.42] | 2.354 | |  | | | 1.45 | |  | 0.545 | | |  | | | 1.2 | |  | | 1.103 | |  | | | 0.94 | | | |  | |
| B6TK27 | | Probable glutathione S-transferase GSTU6 | | K00799|[EC:2.5.1.18] | 2.873 | |  | | | 0.71 | |  | 0.228 | | |  | | | 1.4 | |  | | 1.454 | |  | | | 1.283 | | | |  | |
| C0HHC4 | | Nucleoside diphosphate kinase 1 | | K00940|[EC:2.7.4.6] | 0.802 | |  | | | 0.74 | |  | 0.978 | | |  | | | 0.7 | |  | | 1.137 | |  | | | 1.83 | | | |  | |
| B7ZX17 | | Chaperonin CPN60-2, mitochondrial | | K04077|chaperonin GroEL | 1 | |  | | | 1.02 | |  | 0.9 | | |  | | | 0.9 | |  | | 1.706 | |  | | | 1.777 | | | |  | |
| B4FVB2 | | Elongation factor Tu, mitochondrial | | K02358|elongation factor Tu | 1.347 | |  | | | 1.27 | |  | 0.959 | | |  | | | 0.8 | |  | | 1.09 | |  | | | 1.563 | | | |  | |
| B7ZYI0 | | 14-3-3-like protein GF14-6 | | K06630|tyrosine 3-monooxygenase/tryptophan 5-monooxygenase activation protein | 1.269 | |  | | | 0.78 | |  | 0.518 | | |  | | | 1.2 | |  | | 0.747 | |  | | | 0.638 | | | |  | |
| B4FE06 | | Ras-related protein RABA1f | | K07976|Rab family, other | 1.199 | |  | | | 1.29 | |  | 1.056 | | |  | | | 0.7 | |  | | 0.876 | |  | | | 1.303 | | | |  | |
| B6UHU1 | | Catalase isozyme 1 | | K03781|1|0.0|1032|zma:100857004|catalase [EC:1.11.1.6] | 1.535 | |  | | | 1.19 | |  | 0.639 | | |  | | | 0.9 | |  | | 0.788 | |  | | | 0.895 | | | |  | |
| C5XSS8 | | Presequence protease 1, chloroplastic/mitochondrial | | K06972 | 1.461 | |  | | | 0.84 | |  | 0.513 | | |  | | | 1.2 | |  | | 1.602 | |  | | | 1.37 | | | |  | |
| B8A2Q4 | | Aminopeptidase N | | K01256| [EC:3.4.11.2] | 1.668 | |  | | | 0.68 | |  | 0.444 | | |  | | | 1.3 | |  | | 1.335 | |  | | | 1.014 | | | |  | |
| B6SJ21 | | Guanine nucleotide-binding protein subunit beta-like protein A | | K14753 | 1.817 | |  | | | 1.48 | |  | 0.8 | | |  | | | 0.8 | |  | | 0.781 | |  | | | 0.94 | | | |  | |
| B4FWI0 | | 60S acidic ribosomal protein P0 | | K02941 | 2.11 | |  | | | 1.75 | |  | 1.043 | | |  | | | 1.1 | |  | | 1.199 | |  | | | 1.261 | | | |  | |
| B6UDG4 | | NADP-dependent malic enzyme | | K00029||malate dehydrogenase (oxaloacetate-decarboxylating)(NADP+) [EC:1.1.1.40] | 1.656 | |  | | | 0.98 | |  | 0.532 | | |  | | | 1.2 | |  | | 1.08 | |  | | | 0.882 | | | |  | |
| B6TU74 | | Putative uncharacterized protein | | K03152|4-methyl-5(b-hydroxyethyl)-thiazole monophosphate biosynthesis | 1.606 | |  | | | 0.9 | |  | 0.562 | | |  | | | 1.1 | |  | | 1.39 | |  | | | 1.261 | | | |  | |
| B8A349 | | Serine/threonine-protein phosphatase 2A 65 kDa regulatory subunit A beta isoform | | K03456 | 1.976 | |  | | | 1.41 | |  | 0.694 | | |  | | | 1.2 | |  | | 0.969 | |  | | | 0.816 | | | |  | |
| B4FRI1 | | Phosphoserine aminotransferase, chloroplastic | | K00831|[EC:2.6.1.52] | 1.437 | |  | | | 1.07 | |  | 0.73 | | |  | | | 0.9 | |  | | 0.861 | |  | | | 0.955 | | | |  | |
| Q9ZR86 | | Probable nucleoredoxin 1-1 | | - | 1.506 | |  | | | 0.92 | |  | 0.579 | | |  | | | 0.9 | |  | | 0.909 | |  | | | 1.002 | | | |  | |
| B6UHJ4 | | Elongation factor 1-alpha | | K03231 | 0.96 | |  | | | 1.25 | |  | 1.364 | | |  | | | 0.9 | |  | | 0.862 | |  | | | 1.053 | | | |  | |
| C0HF32 | | ATP synthase subunit beta, mitochondrial | | K02133|F-type H+-transporting ATPase subunit beta [EC:3.6.3.14] | 0.642 | |  | | | 0.98 | |  | 1.652 | | |  | | | 0.6 | |  | | 1.883 | |  | | | 3.088 | | | |  | |
| B4FRG1 | | 14-3-3-like protein (Fragment) | | K06630|tyrosine 3-monooxygenase/tryptophan 5-monooxygenase activation protein | 1.43 | |  | | | 1.06 | |  | 0.724 | | |  | | | 1.1 | |  | | 1.025 | |  | | | 1.045 | | | |  | |
| B6TGF0 | | Proteasome subunit alpha type-3 | | K02727|20S proteasome subunit alpha 7 [EC:3.4.25.1] | 1.34 | |  | | | 1.37 | |  | 1.013 | | |  | | | 1.2 | |  | | 0.726 | |  | | | 0.733 | | | |  | |
| Q5GAQ3 | | Alanine aminotransferase 2 | | K00814|1|2e-84|310|osa:4348524|alanine transaminase [EC:2.6.1.2] | 1.251 | |  | | | 1.01 | |  | 0.785 | | |  | | | 0.9 | |  | | 0.626 | |  | | | 0.894 | | | |  | |
| B4F9G8 | | Pyruvate kinase, cytosolic isozyme | | K00873|[EC:2.7.1.40] | 1.953 | |  | | | 1.1 | |  | 0.523 | | |  | | | 0.9 | |  | | 0.889 | |  | | | 1.019 | | | |  | |
| C5XIY9 | | Dihydrolipoyl dehydrogenase 1, mitochondrial | | K00382|[EC:1.8.1.4] | 1.492 | |  | | | 1.11 | |  | 0.823 | | |  | | | 0.9 | |  | | 1.104 | |  | | | 1.335 | | | |  | |
| B8A367 | | Cysteine synthase, chloroplastic/chromoplastic | | K01738|[EC:2.5.1.47] | 0.826 | |  | | | 1.12 | |  | 1.13 | | |  | | | 1 | |  | | 0.646 | |  | | | 0.693 | | | |  | |
| B4FFZ2 | | Ketol-acid reductoisomerase, chloroplastic | | K00053|1|0.0|1039|zma:100193695|ketol-acid reductoisomerase [EC:1.1.1.86] | 2.498 | |  | | | 1.4 | |  | 0.483 | | |  | | | 0.9 | |  | | 1.682 | |  | | | 2.039 | | | |  | |
| Q43264 | | Alcohol dehydrogenase 1 | | K00001|[EC:1.1.1.1] | 1.343 | |  | | | 2.05 | |  | 1.239 | | |  | | | 1.2 | |  | | 1.346 | |  | | | 1.545 | | | |  | |
| B6TY76 | | Elongation factor 1-gamma 1 | | K03233 | 1.413 | |  | | | 1.09 | |  | 0.794 | | |  | | | 0.7 | |  | | 0.827 | |  | | | 1.026 | | | |  | |
| B4F8X3 | | Peroxisomal acyl-coenzyme A oxidase 1 | | K00232|[EC:1.3.3.6] | 1.632 | |  | | | 1.02 | |  | 0.615 | | |  | | | 1.1 | |  | | 0.763 | |  | | | 0.716 | | | |  | |
| B8A3G8 | | Glycine-rich RNA-binding protein 1 (Fragment) | | K13195|cold-inducible RNA-binding protein!  K02965|small subunit ribosomal protein S19!  K11294|nucleolin!  K12741|heterogeneous nuclear ribonucleoprotein A1/A3 | 0.623 | |  | | | 0.87 | |  | 1.341 | | |  | | | 1.2 | |  | | 0.915 | |  | | | 0.777 | | | |  | |
| C4JBB8 | | Heat shock 70 kDa protein, mitochondrial | | K04043|molecular chaperone DnaK | 1.303 | |  | | | 1.14 | |  | 0.873 | | |  | | | 0.8 | |  | | 0.902 | |  | | | 1.068 | | | |  | |
| B6UHI4 | | V-type proton ATPase subunit B 1 | | K02147|[EC:3.6.3.14] | 0.678 | |  | | | 0.92 | |  | 1.342 | | |  | | | 0.7 | |  | | 0.585 | |  | | | 0.875 | | | |  | |
| B4FDE1 | | Eukaryotic translation initiation factor 5A-3 | | K03263 | 0.771 | |  | | | 0.66 | |  | 0.957 | | |  | | | 1.7 | |  | | 2.693 | |  | | | 1.731 | | | |  | |
| B6TSB3 | | Glucose-6-phosphate 1-dehydrogenase, cytoplasmic isoform | | K00036|[EC:1.1.1.49] | 1.958 | |  | | | 1.09 | |  | 0.509 | | |  | | | 0.9 | |  | | 0.816 | |  | | | 0.738 | | | |  | |
| C4J6W6 | | Alpha-xylosidase | | K15925|[EC:3.2.1.177] | 1.709 | |  | | | 1.6 | |  | 0.858 | | |  | | | 1 | |  | | 1.656 | |  | | | 1.813 | | | |  | |
| B4FNZ1 | | Sucrose-phosphatase 1 | | - | 1.575 | |  | | | 1.09 | |  | 0.647 | | |  | | | 1 | |  | | 0.8 | |  | | | 0.767 | | | |  | |
| C5Z7S0 | | Tubulin beta-7 chain | | K07375 | 1.942 | |  | | | 2.12 | |  | 0.923 | | |  | | | 1.4 | |  | | 1.097 | |  | | | 1.101 | | | |  | |
| B4F9J5 | | Glutamate dehydrogenase | | K00261|[EC:1.4.1.3] | 1.431 | |  | | | 0.66 | |  | 0.38 | | |  | | | 0.9 | |  | | 0.957 | |  | | | 0.91 | | | |  | |
| B6SIM7 | | Proteasome subunit beta type-2 | | K02734|[EC:3.4.25.1] | 1.828 | |  | | | 1.82 | |  | 0.981 | | |  | | | 0.8 | |  | | 1.391 | |  | | | 1.49 | | | |  | |
| C0P2V1 | | Leucine aminopeptidase 2, chloroplastic | | K01255|[EC:3.4.11.1] | 1.621 | |  | | | 0.79 | |  | 0.51 | | |  | | | 0.8 | |  | | 1.898 | |  | | | 2.418 | | | |  | |
| B6SUD1 | | Alcohol dehydrogenase 2 | | K00001|[EC:1.1.1.1] | 1.419 | |  | | | 0.7 | |  | 0.615 | | |  | | | 1 | |  | | 0.918 | |  | | | 0.947 | | | |  | |
| B6TD84 | | IN2-2 protein | | K05275|pyridoxine 4-dehydrogenase [EC:1.1.1.65]!  K00064|D-threo-aldose 1-dehydrogenase [EC:1.1.1.122] | 1.633 | |  | | | 1.42 | |  | 0.828 | | |  | | | 1 | |  | | 1.067 | |  | | | 1.185 | | | |  | |
| B7ZXK5 | | Selenium-binding protein 2 | | - | 1.452 | |  | | | 1.23 | |  | 0.85 | | |  | | | 1 | |  | | 0.983 | |  | | | 0.963 | | | |  | |
| C0HI51 | | Probable aspartyl aminopeptidase | | K01267|[EC:3.4.11.21] | 1.686 | |  | | | 1.04 | |  | 0.526 | | |  | | | 0.9 | |  | | 0.756 | |  | | | 0.721 | | | |  | |
| B3SHC9 | | ATP synthase subunit beta, chloroplastic | | K02112|F-type H+-transporting ATPase subunit beta [EC:3.6.3.14] | 0.793 | |  | | | 1.21 | |  | 1.244 | | |  | | | 0.9 | |  | | 0.868 | |  | | | 0.969 | | | |  | |
| B4FSM5 | | Peroxiredoxin-2F, mitochondrial | | K11187|peroxiredoxin 5, atypical 2-Cys peroxiredoxin [EC:1.11.1.15]!  K03386|peroxiredoxin (alkyl hydroperoxide reductase subunit C) [EC:1.11.1.15] | 1.043 | |  | | | 0.96 | |  | 0.933 | | |  | | | 0.8 | |  | | 1.235 | |  | | | 1.589 | | | |  | |
| B4F8H9 | | Probable 1-aminocyclopropane-1-carboxylate deaminase | | K01505|[EC:3.5.99.7] | 1.428 | |  | | | 1.04 | |  | 0.622 | | |  | | | 0.8 | |  | | 0.655 | |  | | | 0.876 | | | |  | |
| C0P3W9 | | Phosphoenolpyruvate carboxykinase [ATP] | | K01610|[EC:4.1.1.49] | 1.226 | |  | | | 0.8 | |  | 0.653 | | |  | | | 1.2 | |  | | 0.608 | |  | | | 0.481 | | | |  | |
| B6TBN6 | | Pyruvate kinase, cytosolic isozyme | | K00873|[EC:2.7.1.40] | 1.45 | |  | | | 0.86 | |  | 0.505 | | |  | | | 1.6 | |  | | 0.886 | |  | | | 0.576 | | | |  | |
| C0P848 | | Formate dehydrogenase 1, mitochondrial | | K00122|[EC:1.2.1.2] | 1.859 | |  | | | 1.02 | |  | 0.515 | | |  | | | 1 | |  | | 1.296 | |  | | | 1.232 | | | |  | |
| B6U0V6 | | Endoplasmin homolog | | K09487|heat shock protein 90kDa beta | 1.402 | |  | | | 0.71 | |  | 0.579 | | |  | | | 0.8 | |  | | 0.452 | |  | | | 0.58 | | | |  | |
| B6TM55 | | L-ascorbate peroxidase 1, cytosolic | | K00434|[EC:1.11.1.11] | 0.862 | |  | | | 0.69 | |  | 0.731 | | |  | | | 0.9 | |  | | 0.944 | |  | | | 1.123 | | | |  | |
| C4J093 | | Adenylate kinase B | | K00939|[EC:2.7.4.3] | 0.764 | |  | | | 0.71 | |  | 0.914 | | |  | | | 0.9 | |  | | 0.71 | |  | | | 0.81 | | | |  | |
| C4J6X8 | | Stromal 70 kDa heat shock-related protein, chloroplastic (Fragment) | | K03283|heat shock 70kDa protein 1/8 | 0.508 | |  | | | 0.96 | |  | 1.709 | | |  | | | 0.9 | |  | | 1.11 | |  | | | 1.159 | | | |  | |
| B4FBP0 | | T-complex protein 1 subunit eta | | K09499 | 1.334 | |  | | | 1.05 | |  | 0.93 | | |  | | | 1.1 | |  | | 1.063 | |  | | | 0.943 | | | |  | |
| **Oxidative stress** | | | | | | | | | | | | | | | | | | | | | | | | | | | | | | | | |  |
| B6U016 | | Senescence-associated protein DIN1 | | - | 0.66 |  | | 0.67 | | |  | | | 0.982 | | |  | | | 1 | |  | | 1.061 | |  | | | | 0.978 | | | |
| B4FZ35 | | Apolipoprotein D | | K03098|outer membrane lipoprotein Blc | 0.647 |  | | 0.76 | | |  | | | 1.204 | | |  | | | 0.8 | |  | | 0.871 | |  | | | | 1.063 | | | |
| B4FTR5 | | Uncharacterized protein At1g32220, chloroplastic | | - | 0.852 |  | | 0.71 | | |  | | | 0.998 | | |  | | | 0.8 | |  | | 0.791 | |  | | | | 1.033 | | | |
| O81229 | | 60S ribosomal protein L23a O | | K02893 | 0.385 |  | | 1.38 | | |  | | | 4.001 | | |  | | | 2.2 | |  | | 1.625 | |  | | | | 0.843 | | | |
| B6SSX2 | | Gamma-glutamyltranspeptidase 1 | | K00681| [EC:2.3.2.2] | 0.705 |  | | 1.3 | | |  | | | 1.605 | | |  | | | 0.8 | |  | | 0.915 | |  | | | | 1.158 | | | |
| B6TRW8 | | Dihydrolipoyllysine-residue succinyltransferase component of 2-oxoglutarate dehydrogenase complex, mitochondrial (Fragments) | | K00658| [EC:2.3.1.61] | 0.925 |  | | 0.81 | | |  | | | 0.78 | | |  | | | 0.8 | |  | | 1.277 | |  | | | | 1.477 | | | |
| C0PDA6 | | Fumarate hydratase 1, mitochondrial | | K01679| [EC:4.2.1.2] | 1.335 |  | | 1.07 | | |  | | | 0.78 | | |  | | | 0.8 | |  | | 1.225 | |  | | | | 1.359 | | | |
| B4FVJ9 | | Probable glutathione S-transferase GSTU1 | | K00799| [EC:2.5.1.18] | 1.999 |  | | 0.55 | | |  | | | 0.273 | | |  | | | 1.4 | |  | | 2.173 | |  | | | | 1.549 | | | |
| C0PBY7 | | Nucleoside diphosphate kinase IV, chloroplastic/mitochondrial | | K00940| [EC:2.7.4.6] | 1.228 |  | | 0.74 | | |  | | | 0.594 | | |  | | | 0.8 | |  | | 1.054 | |  | | | | 1.455 | | | |
| C0PK64 | | Nucleoside diphosphate kinase IV, chloroplastic/mitochondrial | | K00940| [EC:2.7.4.6] | 1.268 |  | | 0.75 | | |  | | | 0.582 | | |  | | | 0.8 | |  | | 0.982 | |  | | | | 1.256 | | | |
| B4FSM5 | | Peroxiredoxin-2F, mitochondrial | | K11187| [EC:1.11.1.15]!  K03386| [EC:1.11.1.15] | 1.043 |  | | 0.96 | | |  | | | 0.933 | | |  | | | 0.8 | |  | | 1.235 | |  | | | | 1.589 | | | |
| Q6JAH6 | | Probable phospholipid hydroperoxide glutathione peroxidase 6, mitochondrial O | | K00432| [EC:1.11.1.9] | 0.983 |  | | 0.75 | | |  | | | 0.713 | | |  | | | 1.2 | |  | | 1.269 | |  | | | | 1.178 | | | |
| C0P3R8 | | Phospholipid hydroperoxide glutathione peroxidase, chloroplastic | | K00432| [EC:1.11.1.9] | 0.597 |  | | 0.62 | | |  | | | 1.082 | | |  | | | 0.9 | |  | | 1.152 | |  | | | | 1.504 | | | |
| B4G066 | | Methylmalonate-semialdehyde dehydrogenase [acylating], mitochondrial | | K00140| [EC:1.2.1.18 1.2.1.27] | 1.129 |  | | 1.09 | | |  | | | 0.768 | | |  | | | 0.9 | |  | | 0.954 | |  | | | | 1.155 | | | |
| B4G066 | | Methylmalonate-semialdehyde dehydrogenase [acylating], mitochondrial | | K00140| [EC:1.2.1.18 1.2.1.27] | 1.153 |  | | 1.06 | | |  | | | 0.854 | | |  | | | 1 | |  | | 1.445 | |  | | | | 1.389 | | | |
| C0PC75 | | Thioredoxin M2, chloroplastic | | K03671 | 0.365 |  | | 0.68 | | |  | | | 2.056 | | |  | | | 1.2 | |  | | 1.164 | |  | | | | 1.079 | | | |
| B4FRH1 | | Thioredoxin M2, chloroplastic | | K03671 | 0.476 |  | | 0.75 | | |  | | | 1.555 | | |  | | | 1.6 | |  | | 1.218 | |  | | | | 0.794 | | | |
| B6U8S9 | | Peptide methionine sulfoxide reductase A4, chloroplastic | | K07304| [EC:1.8.4.11] | 0.445 |  | | 0.87 | | |  | | | 1.86 | | |  | | | 1.7 | |  | | 1.356 | |  | | | | 0.913 | | | |
| B6TNT5 | | Peptide methionine sulfoxide reductase A2-1 | | K07304| [EC:1.8.4.11] | 0.852 |  | | 1.09 | | |  | | | 1.179 | | |  | | | 1.1 | |  | | 0.853 | |  | | | | 0.84 | | | |
| B6T2N3 | | Peptide methionine sulfoxide reductase B1, chloroplastic | | K07305| [EC:1.8.4.12] | 0.418 |  | | 0.91 | | |  | | | 2.266 | | |  | | | 1.3 | |  | | 1.952 | |  | | | | 1.512 | | | |
| B4FD74 | | Isoflavone reductase homolog IRL | | K00224| [EC:1.3.1.-]!  K13081|leucoanthocyanidin reductase [EC:1.17.1.3] | 2.333 |  | | 3.46 | | |  | | | 1.474 | | |  | | | 1.1 | |  | | 0.789 | |  | | | | 0.751 | | | |
| B4FS17 | | Thylakoid lumenal 29 kDa protein, chloroplastic | | K00434|L-ascorbate peroxidase [EC:1.11.1.11] | 0.365 |  | | 0.73 | | |  | | | 1.905 | | |  | | | 0.9 | |  | | 0.916 | |  | | | | 1.11 | | | |
| C0PBT8 | | Probable L-ascorbate peroxidase 7, chloroplastic | | K00434| [EC:1.11.1.11] | 0.264 |  | | 0.9 | | |  | | | 4.09 | | |  | | | 1.5 | |  | | 1.245 | |  | | | | 0.839 | | | |
| H9BG22 | | Fatty acid alpha-dioxygenase | | K10529|alpha-dioxygenase [EC:1.-.-.-] | 4.011 |  | | 1.99 | | |  | | | 0.429 | | |  | | | 1.2 | |  | | 1.212 | |  | | | | 1.308 | | | |
| B6TEY2 | | Probable L-ascorbate peroxidase 8, chloroplastic | | K00434| [EC:1.11.1.11] | 0.395 |  | | 0.84 | | |  | | | 2.257 | | |  | | | 1.1 | |  | | 1.393 | |  | | | | 1.069 | | | |
| B4FA06 | | Probable L-ascorbate peroxidase 4 | | K00434| [EC:1.11.1.11] | 1.25 |  | | 0.94 | | |  | | | 0.711 | | |  | | | 0.9 | |  | | 1.4 | |  | | | | 1.41 | | | |
| B6SI04 | | Peroxidase 65 | | K00430| [EC:1.11.1.7] | 1.312 |  | | 1.82 | | |  | | | 1.097 | | |  | | | 1.3 | |  | | 2.822 | |  | | | | 2.06 | | | |
| B6SMR2 | | Peroxidase 52 | | K00430| [EC:1.11.1.7] | 0.622 |  | | 0.98 | | |  | | | 1.439 | | |  | | | 0.6 | |  | | 0.341 | |  | | | | 0.529 | | | |
| B6SU07 | | Peroxidase 45 | | K00430| [EC:1.11.1.7] | 1.32 |  | | 0.8 | | |  | | | 0.553 | | |  | | | 0.7 | |  | | 0.99 | |  | | | | 1.344 | | | |
| B6U6W0 | | Peroxidase 4 | | K00430| [EC:1.11.1.7] | 0.606 |  | | 0.6 | | |  | | | 1.1 | | |  | | | 0.7 | |  | | 0.501 | |  | | | | 0.973 | | | |
| B4FH35 | | Peroxidase 2 | | K00430| [EC:1.11.1.7] | 0.549 |  | | 1.32 | | |  | | | 2.151 | | |  | | | 0.7 | |  | | 0.606 | |  | | | | 0.752 | | | |
| B4FU88 | | Peroxidase 1 | | K00430| [EC:1.11.1.7] | 0.876 |  | | 1.26 | | |  | | | 1.337 | | |  | | | 0.9 | |  | | 1.944 | |  | | | | 2.156 | | | |
| B6TMI9 | | Peroxidase 1 | | K00430| [EC:1.11.1.7] | 1.089 |  | | 1.11 | | |  | | | 0.947 | | |  | | | 0.5 | |  | | 0.773 | |  | | | | 1.612 | | | |
| B4FBH0 | | Peroxidase (Fragment) | | K00430| [EC:1.11.1.7] | 1.592 |  | | 2.23 | | |  | | | 1.353 | | |  | | | 1 | |  | | 1.988 | |  | | | | 1.899 | | | |
| C5XYY5 | | Peroxidase (Fragment) | | K00430| [EC:1.11.1.7] | 1.755 |  | | 1.89 | | |  | | | 1.18 | | |  | | | 1 | |  | | 1.216 | |  | | | | 1.159 | | | |
| C5WXV0 | | Peroxidase (Fragment) | | K00430| [EC:1.11.1.7] | 3.966 |  | | 7.37 | | |  | | | 1.905 | | |  | | | 0.3 | |  | | 0.919 | |  | | | | 2.941 | | | |
| B1PEY4 | | Superoxide dismutase [Cu-Zn], chloroplastic | | K04565| [EC:1.15.1.1] | 0.649 |  | | 1.02 | | |  | | | 1.544 | | |  | | | 1.2 | |  | | 1.134 | |  | | | | 1.005 | | | |
| C5XMX0 | | Peroxidase 72 | | K00430| [EC:1.11.1.7] | 0.803 |  | | 1.96 | | |  | | | 2.008 | | |  | | | 0.6 | |  | | 0.803 | |  | | | | 1.442 | | | |
| B6TU39 | | Peroxidase 70 | | K00430| [EC:1.11.1.7] | 2.981 |  | | 2.58 | | |  | | | 1.008 | | |  | | | 1.2 | |  | | 1.724 | |  | | | | 1.675 | | | |
| B4FQI9 | | Peroxidase 59 | | K00430| [EC:1.11.1.7] | 0.666 |  | | 1.06 | | |  | | | 1.964 | | |  | | | 0.4 | |  | | 1.137 | |  | | | | 2.956 | | | |
| B4FYD8 | | Peroxidase 51 | | K00430| [EC:1.11.1.7] | 1.319 |  | | 0.66 | | |  | | | 0.326 | | |  | | | 1.3 | |  | | 2.296 | |  | | | | 1.896 | | | |
| B4FVT1 | | Peroxidase 5 | | K00430| [EC:1.11.1.7] | 0.636 |  | | 1.2 | | |  | | | 1.821 | | |  | | | 0.9 | |  | | 2.162 | |  | | | | 2.721 | | | |
| C5WUE6 | | Peroxidase 5 | | K00430| [EC:1.11.1.7] | 0.657 |  | | 0.5 | | |  | | | 0.836 | | |  | | | 0.6 | |  | | 0.684 | |  | | | | 1.195 | | | |
| C5YLZ0 | | Peroxidase 47 | | K00430| [EC:1.11.1.7] | 0.843 |  | | 0.38 | | |  | | | 0.472 | | |  | | | 0.6 | |  | | 0.864 | |  | | | | 1.625 | | | |
| B6THU9 | | Peroxidase 30 | | K00430| [EC:1.11.1.7] | 1.421 |  | | 2.07 | | |  | | | 1.484 | | |  | | | 0.7 | |  | | 1.287 | |  | | | | 2.012 | | | |
| C0HEE6 | | Peroxidase 17 | | K00430| [EC:1.11.1.7] | 0.692 |  | | 0.94 | | |  | | | 1.274 | | |  | | | 0.5 | |  | | 1.974 | |  | | | | 4.018 | | | |
| B6UB27 | | Peroxidase 1 | | K00430| [EC:1.11.1.7] | 0.809 |  | | 0.51 | | |  | | | 0.617 | | |  | | | 1 | |  | | 0.076 | |  | | | | 0.079 | | | |
| C0PF45 | | Peroxidase (Fragment) | | K00430| [EC:1.11.1.7] | 0.413 |  | | 0.7 | | |  | | | 1.831 | | |  | | | 0.7 | |  | | 0.438 | |  | | | | 0.627 | | | |
| C0HF32 | | ATP synthase subunit beta, mitochondrial | | K02133|F-type H+-transporting ATPase subunit beta [EC:3.6.3.14] | 0.642 |  | | 0.98 | | |  | | | 1.652 | | |  | | | 0.6 | |  | | 1.883 | |  | | | | 3.088 | | | |
| B6SVV9 | | ATP synthase subunit beta, mitochondrial | | K02133|F-type H+-transporting ATPase subunit beta [EC:3.6.3.14] | 1.546 |  | | 0.77 | | |  | | | 0.521 | | |  | | | 1 | |  | | 1.251 | |  | | | | 1.227 | | | |
| C5Z483 | | Reticuline oxidase-like protein | | - | 1.361 |  | | 1.07 | | |  | | | 0.77 | | |  | | | 0.8 | |  | | 0.304 | |  | | | | 0.411 | | | |
| B4FXU2 | | NADH dehydrogenase [ubiquinone] iron-sulfur protein 1, mitochondrial | | K03934| [EC:1.6.5.3 1.6.99.3] | 1.876 |  | | 1.29 | | |  | | | 0.707 | | |  | | | 1.2 | |  | | 1.414 | |  | | | | 1.165 | | | |
| B8A3M9 | | NADH dehydrogenase [ubiquinone] iron-sulfur protein 1, mitochondrial | | K03934| [EC:1.6.5.3 1.6.99.3] | 0.869 |  | | 1.15 | | |  | | | 1.135 | | |  | | | 1 | |  | | 1.252 | |  | | | | 1.297 | | | |
| **Signaling** | |  | |  |  |  | |  | | |  | | |  | | |  | | |  | |  | |  | |  | | | |  | | | |
| B4G0R1 | | Nucleoside diphosphate kinase 2, chloroplastic | | K00940| [EC:2.7.4.6] | 0.89 |  | | 0.92 | | |  | | | 0.911 | | |  | | | 0.8 | |  | | 0.835 | |  | | | | 0.947 | | | |
| B4FUA8 | | Calreticulin-3 | | K08057 | 0.702 |  | | 0.42 | | |  | | | 0.584 | | |  | | | 0.8 | |  | | 0.289 | |  | | | | 0.385 | | | |
| B6TBX3 | | GTP-binding nuclear protein Ran-2 | | K07936 | 0.983 |  | | 1.12 | | |  | | | 1.096 | | |  | | | 1 | |  | | 0.897 | |  | | | | 0.854 | | | |
| B6TWG6 | | COP9 signalosome complex subunit 6a (Fragments) | | K12179 | 1.565 |  | | 0.97 | | |  | | | 0.609 | | |  | | | 0.8 | |  | | 0.667 | |  | | | | 0.878 | | | |
| B6SPL7 | | Copper transport protein ATOX1 | | K07213 | 0.887 |  | | 1.43 | | |  | | | 1.622 | | |  | | | 1.1 | |  | | 1.294 | |  | | | | 1.19 | | | |
| C4J0W6 | | Abscisic acid receptor PYL1 | | K14496|abscisic acid receptor PYR/PYL family | 1.121 |  | | 1.65 | | |  | | | 1.605 | | |  | | | 0.7 | |  | | 0.742 | |  | | | | 0.964 | | | |
| C4J9M7 | | 2-Cys peroxiredoxin BAS1, chloroplastic | | K03386| [EC:1.11.1.15] | 0.831 |  | | 0.64 | | |  | | | 0.738 | | |  | | | 1 | |  | | 1.766 | |  | | | | 1.74 | | | |
| B4G0H7 | | Heme-binding-like protein At3g10130, chloroplastic | | - | 0.371 |  | | 0.84 | | |  | | | 2.563 | | |  | | | 1 | |  | | 1.166 | |  | | | | 1.397 | | | |
| B4FAE1 | | Ras-related protein RIC1 | | K07874|Ras-related protein Rab-1A!  K07976|Rab family, other | 1.37 |  | | 1.03 | | |  | | | 0.701 | | |  | | | 1 | |  | | 1.028 | |  | | | | 0.912 | | | |
| F2CS21 | | Calmodulin-1 | | K02183 | 0.396 |  | | 1.2 | | |  | | | 2.892 | | |  | | | 1 | |  | | 1.305 | |  | | | | 1.154 | | | |
| B8A021 | | Phospholipase D alpha 1 | | K01115 [EC:3.1.4.4] | 1.743 |  | | 1.13 | | |  | | | 0.572 | | |  | | | 1.1 | |  | | 0.892 | |  | | | | 0.775 | | | |
| B4FBJ7 | | 26S protease regulatory subunit 6A homolog | | K03065 | 1.655 |  | | 1.51 | | |  | | | 0.946 | | |  | | | 1 | |  | | 1.337 | |  | | | | 1.327 | | | |
| C0P3S3 | | Calreticulin-3 | | K08057 | 1.455 |  | | 1.38 | | |  | | | 0.932 | | |  | | | 1.2 | |  | | 0.414 | |  | | | | 0.351 | | | |
| C0PDG8 | | Putative peptidase pfaP | | K04773|protease IV [EC:3.4.21.-] | 1.027 |  | | 0.84 | | |  | | | 0.959 | | |  | | | 0.9 | |  | | 0.715 | |  | | | | 0.76 | | | |
| B4FIX8 | | ADP-ribosylation factor 2 | | K07977|Arf/Sar family, other | 1.797 |  | | 0.95 | | |  | | | 0.472 | | |  | | | 1.1 | |  | | 1.18 | |  | | | | 1.092 | | | |
| B6U232 | | Ras-related protein Rab-2-B | | K07976|Rab family, other!  K07878|Ras-related protein Rab-2B!  K07877|Ras-related protein Rab-2A | 1.152 |  | | 1.65 | | |  | | | 1.263 | | |  | | | 0.7 | |  | | 0.796 | |  | | | | 0.994 | | | |
| B6SS20 | | Phototropin-2 | | K08282|non-specific serine/threonine protein kinase [EC:2.7.11.1] | 0.786 |  | | 0.93 | | |  | | | 1.311 | | |  | | | 1.1 | |  | | 1.62 | |  | | | | 1.775 | | | |
| B4FUX3 | | Putative uncharacterized protein | | - | 1.406 |  | | 1.27 | | |  | | | 0.782 | | |  | | | 1 | |  | | 1.108 | |  | | | | 1.366 | | | |
| B4FPT6 | | GTP-binding protein SAR1A | | K07953| [EC:3.6.5.-] | 1.65 |  | | 0.91 | | |  | | | 0.487 | | |  | | | 1.3 | |  | | 1.15 | |  | | | | 1.124 | | | |
| C0HHT0 | | Phospholipase D alpha 1 | | K01115| [EC:3.1.4.4] | 1.766 |  | | 1 | | |  | | | 0.488 | | |  | | | 1.3 | |  | | 1.114 | |  | | | | 0.95 | | | |
| C0PIU9 | | Hexokinase-5 | | K00844| [EC:2.7.1.1] | 2.502 |  | | 1.59 | | |  | | | 0.483 | | |  | | | 0.9 | |  | | 0.57 | |  | | | | 0.651 | | | |
| B4FEK7 | | Protein THYLAKOID FORMATION1, chloroplastic | | - | 1.23 |  | | 0.8 | | |  | | | 0.651 | | |  | | | 1.3 | |  | | 1.364 | |  | | | | 1.182 | | | |
| C5XJZ6 | | Hexokinase-6 | | K00844| [EC:2.7.1.1] | 2.568 |  | | 1.03 | | |  | | | 0.427 | | |  | | | 1 | |  | | 0.788 | |  | | | | 0.903 | | | |
| Q41761 | | Beta-glucosidase, chloroplastic | | K01188| [EC:3.2.1.21] | 2.105 |  | | 1.01 | | |  | | | 0.463 | | |  | | | 0.8 | |  | | 1.342 | |  | | | | 1.585 | | | |
| G1FMQ3 | | Auxin-binding protein 1 | | - | 1.804 |  | | 2.02 | | |  | | | 1.034 | | |  | | | 0.8 | |  | | 1.031 | |  | | | | 1.313 | | | |
| C5YP89 | | Auxin-binding protein 5 (Fragment) | | - | 1.234 |  | | 1.05 | | |  | | | 0.758 | | |  | | | 0.9 | |  | | 1.159 | |  | | | | 1.455 | | | |
| B6SHD8 | | Beta-glucosidase, chloroplastic | | K01188| [EC:3.2.1.21] | 1.846 |  | | 1.09 | | |  | | | 0.574 | | |  | | | 1 | |  | | 1.147 | |  | | | | 1.171 | | | |
| B4F8W9 | | Phosphoinositide phospholipase C 2 | | K05857| [EC:3.1.4.11] | 1.038 |  | | 0.76 | | |  | | | 0.656 | | |  | | | 1 | |  | | 0.816 | |  | | | | 0.816 | | | |
| B6TGS8 | | 6,7-dimethyl-8-ribityllumazine synthase, chloroplastic | | K00794| [EC:2.5.1.78] | 0.791 |  | | 0.65 | | |  | | | 0.807 | | |  | | | 0.9 | |  | | 1.311 | |  | | | | 1.509 | | | |
| C0PES7 | | 3'(2'),5'-bisphosphate nucleotidase | | K15422|3'(2'), 5'-bisphosphate nucleotidase / inositol polyphosphate 1-phosphatase [EC:3.1.3.7 3.1.3.57] | 1.033 |  | | 1.02 | | |  | | | 0.888 | | |  | | | 1.4 | |  | | 1.631 | |  | | | | 1.184 | | | |
| B4FQV3 | | Protein SGT1 homolog | | K12795|suppressor of G2 allele of SKP1 | 0.541 |  | | 0.77 | | |  | | | 1.392 | | |  | | | 0.9 | |  | | 0.575 | |  | | | | 0.621 | | | |
| D0EP01 | | Protein SGT1 homolog | | K12795|suppressor of G2 allele of SKP1 | 0.517 |  | | 1.28 | | |  | | | 2.428 | | |  | | | 0.9 | |  | | 0.679 | |  | | | | 0.802 | | | |
| B6TFS9 | | 14-3-3-like protein GF14-A | | K06630|tyrosine 3-monooxygenase/tryptophan 5-monooxygenase activation protein | 1.542 |  | | 0.98 | | |  | | | 0.719 | | |  | | | 1.2 | |  | | 0.949 | |  | | | | 0.737 | | | |
| B6U4K6 | | Hexokinase-6 | | K00844| [EC:2.7.1.1] | 1.955 |  | | 1.03 | | |  | | | 0.592 | | |  | | | 1.2 | |  | | 1.045 | |  | | | | 0.956 | | | |
| C0PN94 | | Ras-related protein RIC2 | | K07976|Rab family, other | 1.744 |  | | 1.31 | | |  | | | 0.781 | | |  | | | 0.7 | |  | | 0.829 | |  | | | | 1.107 | | | |
| C0PB44 | | Probable LL-diaminopimelate aminotransferase, chloroplastic | | K10206| [EC:2.6.1.83] | 1.136 |  | | 0.88 | | |  | | | 0.769 | | |  | | | 1.1 | |  | | 1.051 | |  | | | | 1.019 | | | |
| C5XVV1 | | Putative uncharacterized protein Sb04g037070 | | - | 0.65 |  | | 0.78 | | |  | | | 1.196 | | |  | | | 1 | |  | | 0.755 | |  | | | | 0.74 | | | |
| C0PK92 | | Abscisic acid receptor PYL2 | | K14496|abscisic acid receptor PYR/PYL family | 0.719 |  | | 1.48 | | |  | | | 1.862 | | |  | | | 0.5 | |  | | 0.73 | |  | | | | 1.606 | | | |
| B4FE06 | | Ras-related protein RABA1f | | K07976|Rab family, other | 1.199 |  | | 1.29 | | |  | | | 1.056 | | |  | | | 0.7 | |  | | 0.876 | |  | | | | 1.303 | | | |
| B6TYH8 | | Sphingosine kinase A | | K04718| [EC:2.7.1.91] | 1.993 |  | | 0.79 | | |  | | | 0.39 | | |  | | | 1 | |  | | 1.001 | |  | | | | 0.993 | | | |
| B4G1R3 | | Ras-related protein RABH1b | | K07893|Ras-related protein Rab-6A | 1.764 |  | | 1.27 | | |  | | | 0.709 | | |  | | | 0.8 | |  | | 0.945 | |  | | | | 1.124 | | | |
| B8A349 | | Serine/threonine-protein phosphatase 2A 65 kDa regulatory subunit A beta isoform | | K03456 | 1.976 |  | | 1.41 | | |  | | | 0.694 | | |  | | | 1.2 | |  | | 0.969 | |  | | | | 0.816 | | | |
| B4FQG7 | | PI-PLC X domain-containing protein At5g67130 | | - | 0.607 |  | | 1.05 | | |  | | | 1.91 | | |  | | | 0.7 | |  | | 1.705 | |  | | | | 2.282 | | | |
| B6UHI4 | | V-type proton ATPase subunit B 1 | | K02147|V-type H+-transporting ATPase subunit B [EC:3.6.3.14] | 0.678 |  | | 0.92 | | |  | | | 1.342 | | |  | | | 0.7 | |  | | 0.585 | |  | | | | 0.875 | | | |
| B6TVN4 | | PI-PLC X domain-containing protein At5g67130 | | - | 1.792 |  | | 1 | | |  | | | 0.621 | | |  | | | 0.5 | |  | | 1.397 | |  | | | | 3.329 | | | |
| **Temperature** | |  | |  |  |  | |  | | |  | | |  | | |  | | |  | |  | |  | |  | | | |  | | | |
| C0HHY8 | | Probable tocopherol cyclase, chloroplastic | | K09834 | 1.238 |  | | 1.328 | | |  | | | 1.008 | | |  | | | 0.7 | |  | | 0.666 | |  | | | | 0.927 | | | |
| Transcription factor | |  | |  |  |  | |  | | |  | | |  | | |  | | |  | |  | |  | |  | | | |  | | | |
| B7ZZR7 | | Transcription factor MYB1R1 | | - | 0.87 |  | | 1.78 | | |  | | | 2.01 | | |  | | | 1.2 | |  | | 0.84 | |  | | | | 0.723 | | | |
| B4FL49 | | Transcription factor BTF3 homolog 4 | | K01527|nascent polypeptide-associated complex subunit beta | 0.76 |  | | 1.42 | | |  | | | 2.05 | | |  | | | 1.5 | |  | | 0.985 | |  | | | | 0.728 | | | |
| B4FLN7 | | Transcription factor Pur-alpha 1 | | - | 0.889 |  | | 1.36 | | |  | | | 1.5 | | |  | | | 0.8 | |  | | 1.058 | |  | | | | 1.132 | | | |
| G3BGW2 | | Transcription factor TCP14 | | K01501|nitrilase [EC:3.5.5.1] | 0.41 |  | | 1.13 | | |  | | | 3.65 | | |  | | | 1.2 | |  | | 1.024 | |  | | | | 0.779 | | | |
| C0HEE9 | | Transcription factor BTF3 homolog 4 | | K01527|nascent polypeptide-associated complex subunit beta | 0.628 |  | | 1.62 | | |  | | | 2.06 | | |  | | | 1.1 | |  | | 0.742 | |  | | | | 0.679 | | | |
| D4P940 | | Transcription factor BTF3 homolog 4 | | K01527|nascent polypeptide-associated complex subunit beta | 0.558 |  | | 1.37 | | |  | | | 2.51 | | |  | | | 1.7 | |  | | 1.169 | |  | | | | 0.611 | | | |
| **Plasmodesma** | | | | | | | | | | | | | | | | | | | | | | | | | | | | | | | | |  |
| B4FQX1 | | Alpha-1,4-glucan-protein synthase [UDP-forming] | | K13379|reversibly glycosylated polypeptide / UDP-arabinopyranose mutase [EC:2.4.1.- 5.4.99.30] | 1.683 |  | | 0.677 | | |  | | | 0.396 | | |  | | | 1.4 | |  | | 0.514 | |  | | | | 0.381 | | | |
| B4FUC4 | | Salt stress root protein RS1 | | - | 0.306 |  | | 0.941 | | |  | | | 3.095 | | |  | | | 1.1 | |  | | 1.994 | |  | | | | 1.604 | | | |
| B4FRH8 | | Actin-7 | | K10355|actin, other eukaryote!K05692|actin beta/gamma 1 | 0.926 |  | | 1.117 | | |  | | | 1.218 | | |  | | | 0.782 | |  | | 0.349 | |  | | | | 0.433 | | | |
| **Heat shock protein/Hsp** | | | | | | | | | | | | | | | | | | | | | | | | | | | | | | | | |  |
| B4FRL9 | | Heat shock cognate 70 kDa protein 2 | | K03283|heat shock 70kDa protein 1/8 | 1.454 |  | | 0.79 | | |  | | | 0.53 | | |  | | | 0.8 | |  | | 1.373 | |  | | | | 1.577 | | | |
| B6TM52 | | 23.6 kDa heat shock protein, mitochondrial | | - | 1.259 |  | | 1.58 | | |  | | | 0.98 | | |  | | | 0.7 | |  | | 2.371 | |  | | | | 3.63 | | | |
| C5YWZ7 | | 16.0 kDa heat shock protein, peroxisomal | | K13993|HSP20 family protein | 0.66 |  | | 1.13 | | |  | | | 1.47 | | |  | | | 0.9 | |  | | 1.865 | |  | | | | 2.356 | | | |
| C0P732 | | Heat shock protein STI | | K09553|stress-induced-phosphoprotein 1 | 0.347 |  | | 1 | | |  | | | 2.62 | | |  | | | 0.8 | |  | | 1.234 | |  | | | | 1.51 | | | |
| B4FQT3 | | Heat shock protein STI | | K09553|stress-induced-phosphoprotein 1 | 0.524 |  | | 1.36 | | |  | | | 2.97 | | |  | | | 1.1 | |  | | 0.924 | |  | | | | 0.969 | | | |
| B4FES7 | | 25.3 kDa heat shock protein, chloroplastic | | K13993|HSP20 family protein | 1.264 |  | | 1.5 | | |  | | | 1.21 | | |  | | | 0.8 | |  | | 2.06 | |  | | | | 2.831 | | | |
| B7ZZJ4 | | Heat shock 70 kDa protein 14 | | K09489|heat shock 70kDa protein 4 | 1.433 |  | | 1.58 | | |  | | | 1.06 | | |  | | | 1 | |  | | 0.961 | |  | | | | 1.089 | | | |
| B8A3D0 | | Stromal 70 kDa heat shock-related protein, chloroplastic (Fragment) | | K03283|heat shock 70kDa protein 1/8 | 0.583 |  | | 0.9 | | |  | | | 1.7 | | |  | | | 0.9 | |  | | 1.419 | |  | | | | 1.628 | | | |
| O64960 | | 24.1 kDa heat shock protein, mitochondrial | | K13993|HSP20 family protein | 0.398 |  | | 1.47 | | |  | | | 3.15 | | |  | | | 0.3 | |  | | 0.658 | |  | | | | 2.394 | | | |
| B6U7Y1 | | Activator of 90 kDa heat shock protein ATPase homolog 2 | | - | 1.07 |  | | 1.09 | | |  | | | 0.94 | | |  | | | 0.7 | |  | | 0.775 | |  | | | | 1.273 | | | |
| C3UZ63 | | Heat shock protein 81-1 | | K04079|molecular chaperone HtpG | 1.188 |  | | 1.04 | | |  | | | 0.97 | | |  | | | 0.9 | |  | | 0.633 | |  | | | | 0.835 | | | |
| C5YU58 | | Stromal 70 kDa heat shock-related protein, chloroplastic (Fragment) | | K03283|heat shock 70kDa protein 1/8 | 0.508 |  | | 1.2 | | |  | | | 2.03 | | |  | | | 0.6 | |  | | 0.632 | |  | | | | 1.028 | | | |
| B7ZZ42 | | Heat shock cognate 70 kDa protein 2 | | K03283|heat shock 70kDa protein 1/8 | 0.684 |  | | 1.39 | | |  | | | 1.68 | | |  | | | 1.4 | |  | | 1.292 | |  | | | | 1.209 | | | |
| C5YPZ1 | | Heat shock cognate 70 kDa protein 2 | | K03283|heat shock 70kDa protein 1/8 | 0.665 |  | | 1.05 | | |  | | | 1.54 | | |  | | | 2.3 | |  | | 3.498 | |  | | | | 1.514 | | | |
| B6TVT8 | | 17.6 kDa class I heat shock protein | | K13993|HSP20 family protein | 0.752 |  | | 1.4 | | |  | | | 2.25 | | |  | | | 1.2 | |  | | 1.206 | |  | | | | 1.012 | | | |
| B4F9E8 | | 18.6 kDa class III heat shock protein | | K13993|HSP20 family protein | 0.745 |  | | 1.5 | | |  | | | 1.86 | | |  | | | 0.7 | |  | | 1.522 | |  | | | | 1.838 | | | |
| B6SZ69 | | Heat shock cognate 70 kDa protein | | K03283|heat shock 70kDa protein 1/8 | 0.715 |  | | 0.89 | | |  | | | 1.27 | | |  | | | 0.9 | |  | | 1.363 | |  | | | | 1.555 | | | |
| C4J410 | | Heat shock 70 kDa protein | | K03283|heat shock 70kDa protein 1/8 | 0.966 |  | | 1 | | |  | | | 0.91 | | |  | | | 0.8 | |  | | 0.985 | |  | | | | 1.222 | | | |
| C5X3T6 | | Heat shock protein 81-3 | | K04079|molecular chaperone HtpG | 2.086 |  | | 1.19 | | |  | | | 0.53 | | |  | | | 0.9 | |  | | 0.834 | |  | | | | 1.043 | | | |
| C4J4W3 | | Heat shock protein STI | | K09553|stress-induced-phosphoprotein 1 | 0.389 |  | | 1.1 | | |  | | | 2.42 | | |  | | | 1.2 | |  | | 1.403 | |  | | | | 1.317 | | | |
| B4FLU9 | | Heat shock 70 kDa protein 14 | | K09489|heat shock 70kDa protein 4 | 1.45 |  | | 1.11 | | |  | | | 0.83 | | |  | | | 0.9 | |  | | 0.836 | |  | | | | 0.914 | | | |
| C0PPG1 | | 23.6 kDa heat shock protein, mitochondrial | | K13993|1|2e-35|146|pop:POPTR_769024|HSP20 family protein | 0.333 |  | | 0.97 | | |  | | | 2.39 | | |  | | | 0.6 | |  | | 1.736 | |  | | | | 3.322 | | | |
| B6U4A3 | | Heat shock 70 kDa protein, mitochondrial | | K04043|molecular chaperone DnaK | 1.362 |  | | 1.08 | | |  | | | 0.79 | | |  | | | 0.8 | |  | | 1.177 | |  | | | | 1.506 | | | |
| C4JBB8 | | Heat shock 70 kDa protein, mitochondrial | | K04043|molecular chaperone DnaK | 1.303 |  | | 1.14 | | |  | | | 0.87 | | |  | | | 0.8 | |  | | 0.902 | |  | | | | 1.068 | | | |
| B8A0J2 | | Heat shock 70 kDa protein 17 | | K09486|hypoxia up-regulated 1 | 0.881 |  | | 0.91 | | |  | | | 1.06 | | |  | | | 0.7 | |  | | 0.917 | |  | | | | 1.253 | | | |
| C4J6X8 | | Stromal 70 kDa heat shock-related protein, chloroplastic (Fragment) | | K03283|heat shock 70kDa protein 1/8 | 0.508 |  | | 0.96 | | |  | | | 1.71 | | |  | | | 0.9 | |  | | 1.11 | |  | | | | 1.159 | | | |
| **Long-day** | |  | |  |  |  | |  | | |  | | |  | | |  | | |  | |  | |  | |  | | | |  | | | |
| B7ZZT1 | | Actin-related protein 4 | | K11652 | 1.577 |  | | 1.28 | | |  | | | 0.8 | | |  | | | 0.8 | |  | | 1.368 | |  | | | | 1.726 | | | |
| **Short-day** | |  | |  |  |  | |  | | |  | | |  | | |  | | |  | |  | |  | |  | | | |  | | | |
| A8WES5 | | Protein HEADING DATE 3B | | - | 1.116 |  | | 1.16 | | |  | | | 1.08 | | |  | | | 0.7 | |  | | 3.363 | |  | | | | 4.911 | | | |
