# Supplementary figures and images for: A photoperiod-responsive protein compendium and conceptual proteome roadmap outline in maize grown in growth chambers with controlled conditions
Source: PLoS One. 2017 Apr 11;12(4):e0174003. doi: 10.1371/journal.pone.0174003 (PMC5388471; doi:10.1371/journal.pone.0174003)

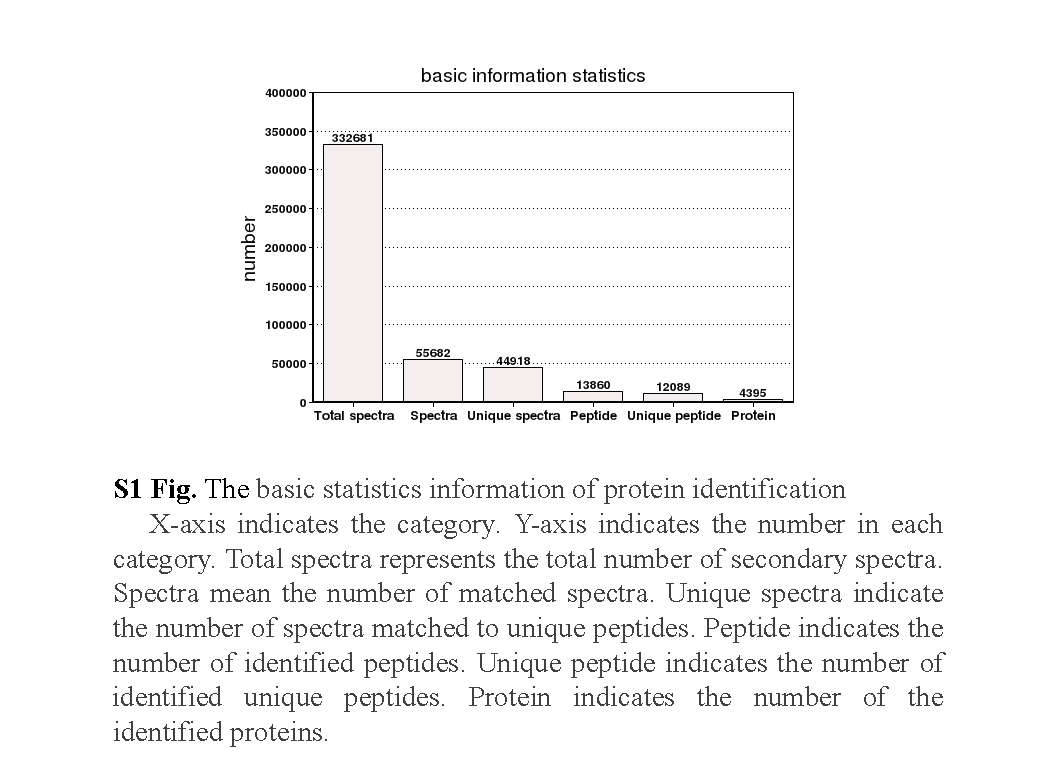

Supplement: S1 Fig — (TIFF) [file pone.0174003.s011.tiff]

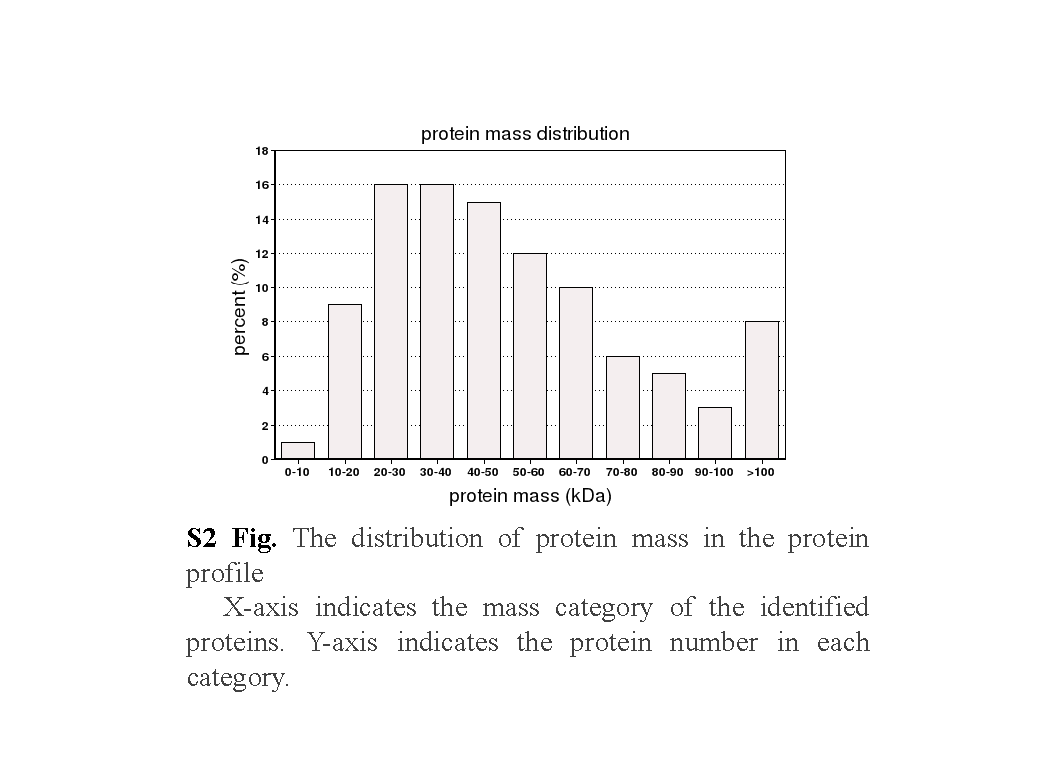

Supplement: S2 Fig — (TIFF) [file pone.0174003.s012.tiff]

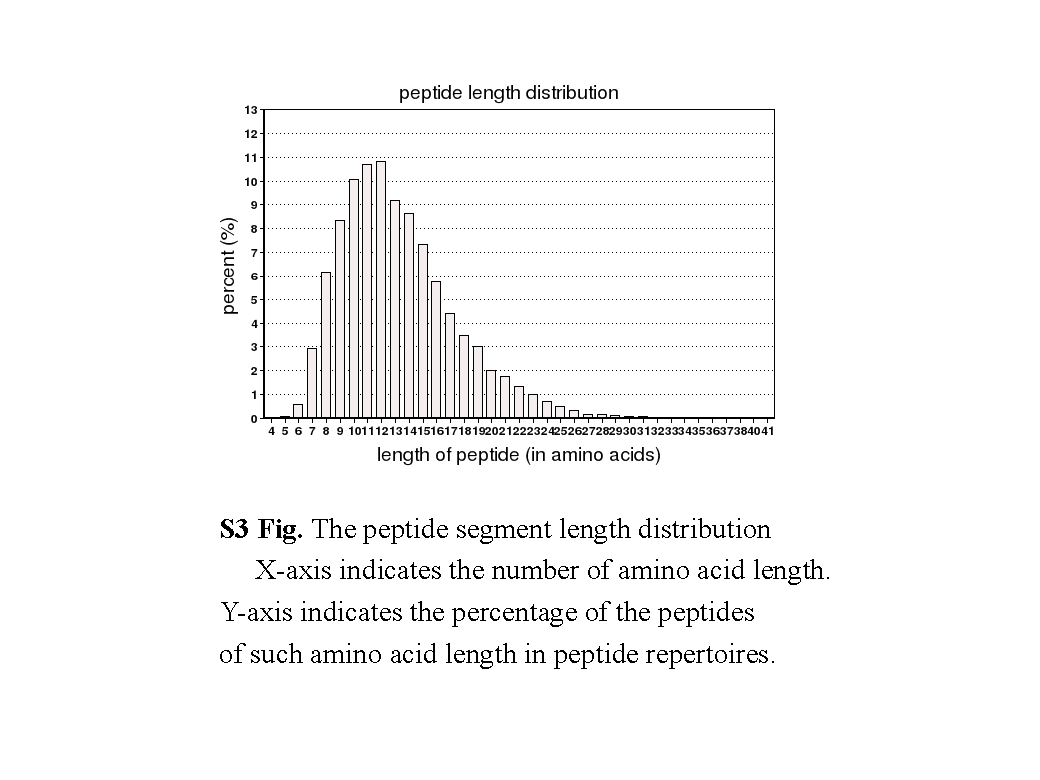

Supplement: S3 Fig — (TIFF) [file pone.0174003.s013.tiff]

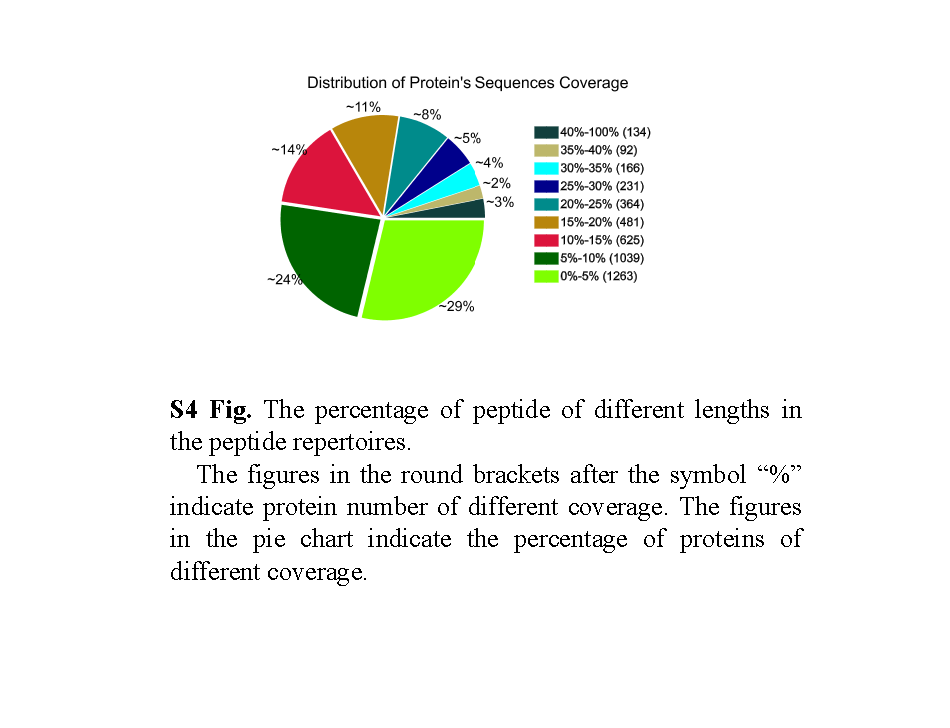

Supplement: S4 Fig — (TIFF) [file pone.0174003.s014.tiff]

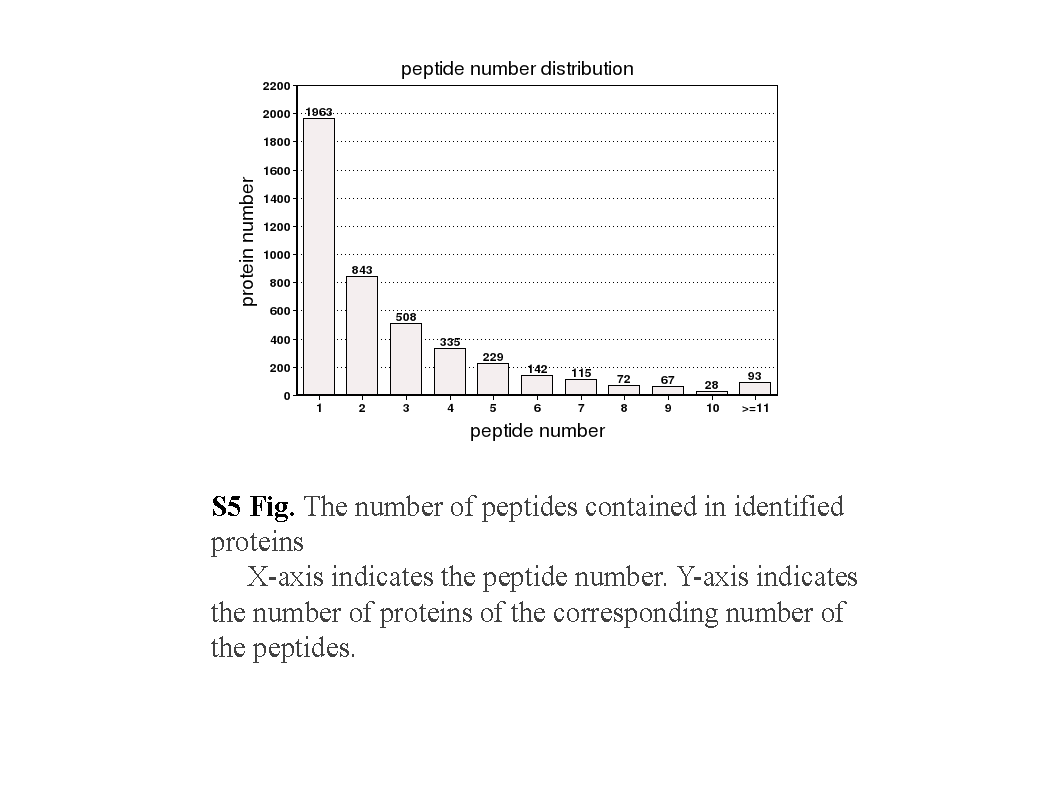

Supplement: S5 Fig — (TIFF) [file pone.0174003.s015.tiff]

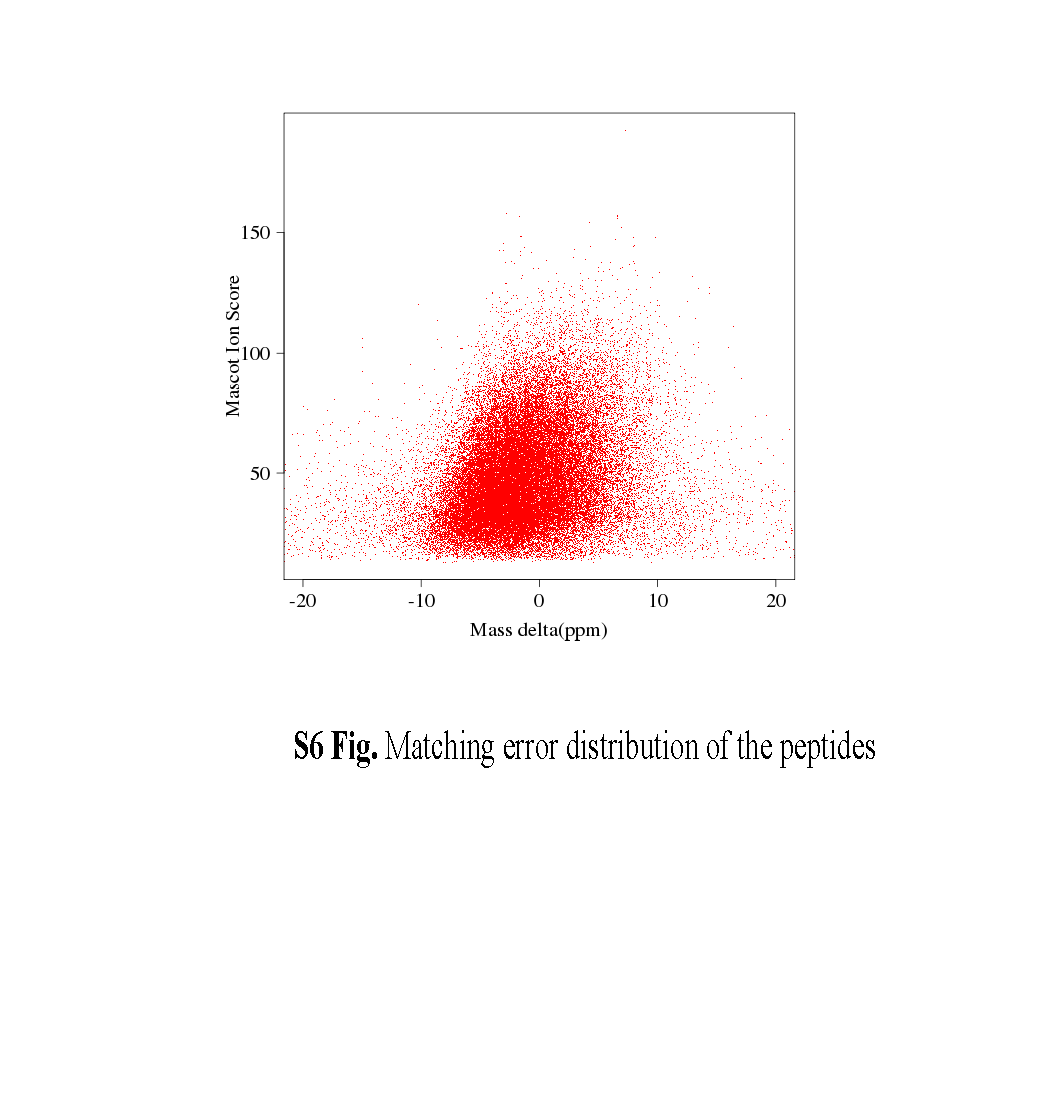

Supplement: S6 Fig — (TIFF) [file pone.0174003.s016.tiff]

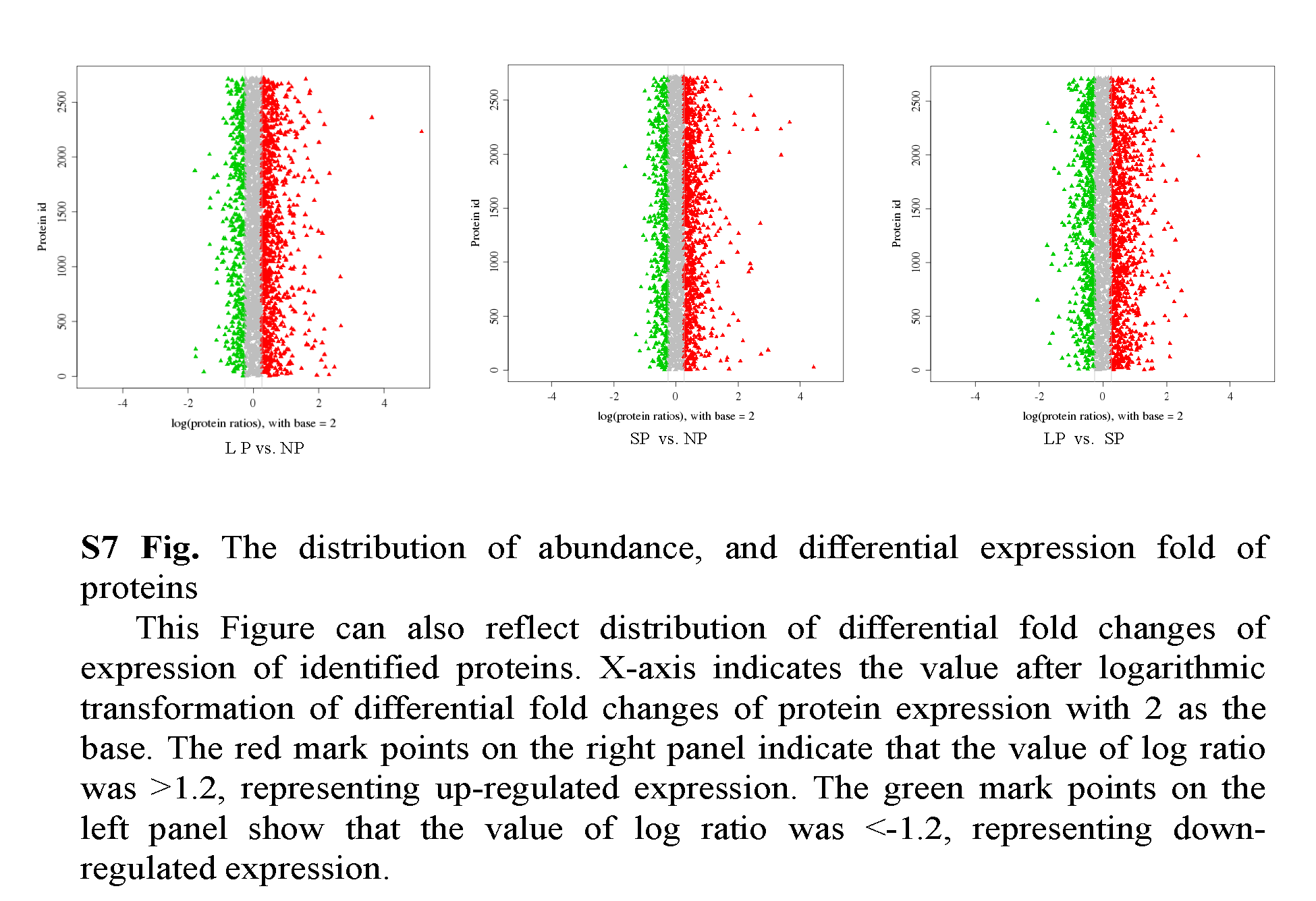

Supplement: S7 Fig — (TIFF) [file pone.0174003.s017.tiff]
